# Supplementary material for: Cost-effectiveness of broadly neutralizing antibodies for HIV prophylaxis for infants born in settings with high HIV burdens
Source: PLoS One. 2025 Mar 19;20(3):e0318940. doi: 10.1371/journal.pone.0318940 (PMC11922280; doi:10.1371/journal.pone.0318940)
Supplement: S1 File — (DOCX) [file pone.0318940.s001.docx]

**Cost-effectiveness of broadly neutralizing antibodies for HIV prophylaxis for infants born in settings with high HIV burdens**

**Appendix**

Contents

[**Supplementary Methods** 3](#_Toc118193091)

[Cohort weighting 3](#_Toc118193092)

[Additional modeled strategies 3](#_Toc118193093)

[Infant HIV prophylaxis model structure 4](#_Toc118193094)

[Oral infant prophylaxis regimens and efficacy 4](#_Toc118193095)

[BNAb efficacy and duration 5](#_Toc118193096)

[BNAb cost estimates 6](#_Toc118193097)

[Determining cost-effectiveness 6](#_Toc118193098)

[**Supplementary Figure 1.** CEPAC-P infant prophylaxis module flowchart 8](#_Toc118193099)

[**Supplementary Figure 2.** Schematic of modeled cohorts and bNAb strategies 9](#_Toc118193100)

[**Supplementary Figure 3.** Efficiency frontier of all bNAb strategies assessed, by country 10](#_Toc118193101)

[**Supplementary Figure 4.** Cumulative vertical transmission in standard-of-care and bNAb strategies, by country 11](#_Toc118193102)

[**Supplementary Figure 5.** Breakdown of intrauterine/intrapartum infections occurring in each exposure group 12](#_Toc118193103)

[**Supplementary Figure 6.** Cost-effective bNAb strategy across influential univariate sensitivity analyses, by country 13](#_Toc118193104)

[**Supplementary Figure 7.** Cost-effective bNAb strategy across non-influential univariate sensitivity analyses, by country 14](#_Toc118193105)

[**Supplementary Figure 8.** Cost-effectiveness results of a three-way sensitivity analysis assessing bNAb efficacy, cost, and cost-effectiveness threshold, by country 15](#_Toc118193106)

[**Supplementary Figure 9.** Cost-effectiveness results of a three-way sensitivity analysis assessing bNAb efficacy, cost, and efficacy duration, by country 16](#_Toc118193107)

[**Supplementary Table 1.** Consolidated Health Economic Evaluation Reporting Standards (CHEERS) checklist 17](#_Toc118193108)

[**Supplementary Table 2.** Extended model input parameters 18](#_Toc118193109)

[**Supplementary Table 3.** Justification of sensitivity analysis ranges 23](#_Toc118193110)

[**Supplementary Table 4.** Validation of CEPAC-P infant HIV vertical transmission projections 27](#_Toc118193111)

[**Supplementary Table 5.** Clinical and economic outcomes of all modeled bNAb infant prophylaxis programs, by country 28](#_Toc118193112)

[**Supplementary Table 6.** Scenario analysis: bNAbs do not reduce intrapartum transmission (base case: 70% reduction) 30](#_Toc118193113)

[**Supplementary Table 7.** Scenario analysis: bNAbs replace WHO-recommended standard-of-care oral infant prophylaxis 31](#_Toc118193114)

[**Supplementary Table 8.** Scenario analysis: maternal HIV prevalence and incidence 32](#_Toc118193115)

[**Supplementary Table 9.** One-way sensitivity analysis: bNAb efficacy against intrapartum and postnatal transmission (base case: 70% reduction) 34](#_Toc118193116)

[**Supplementary Table 10.** One-way sensitivity analysis: bNAb cost (base case: $20/dose) 36](#_Toc118193117)

[**Supplementary Table 11.** One-way sensitivity analysis: bNAb effect duration (base case: 3 months) 38](#_Toc118193118)

[**Supplementary Table 12.** One-way sensitivity analysis: bNAb uptake 40](#_Toc118193119)

[**Supplementary Table 13.** One-way sensitivity analysis: proportion of mothers on antiretroviral therapy during pregnancy 42](#_Toc118193120)

[**Supplementary Table 14.** One-way sensitivity analysis: breastfeeding duration 44](#_Toc118193121)

[**Supplementary Table 15.** One-way sensitivity analysis: maternal postpartum HIV incidence 46](#_Toc118193122)

[**Supplementary Table 16.** One-way sensitivity analysis: antiretroviral therapy treatment cost 48](#_Toc118193123)

[**Supplementary Table 17.** One-way sensitivity analysis: postpartum vertical transmission risk 50](#_Toc118193124)

[**Supplementary Table 18.** One-way sensitivity analysis: infant oral prophylaxis efficacy (base case: 69% intrapartum efficacy and 71% postpartum efficacy) 52](#_Toc118193125)

[**Supplementary Table 19.** One-way sensitivity analysis: additional cost of identifying high-risk infants 54](#_Toc118193126)

[**Supplementary Table 20.** One-way sensitivity analysis: maternal knowledge of acute HIV infection 56](#_Toc118193127)

[**Supplementary Table 21.** One-way sensitivity analysis: maternal knowledge of chronic HIV infection 58](#_Toc118193128)

[**Supplementary Table 22.** One-way sensitivity analysis: postpartum maternal retention in care 60](#_Toc118193129)

[**References** 62](#_Toc118193130)

# **Supplementary Methods**

These supplementary methods provide additional details regarding modeling methodology, input specification, and derivation of key parameters. The Cost-Effectiveness of Preventing AIDS Complications – Pediatric (CEPAC-P) model has previously been validated and additional information is available in previously published works.^1–11^ For further details regarding the mathematical formulas used in the model, model flowcharts, and opportunities for collaboration, we direct readers to the CEPAC website: <https://mpec.massgeneral.org/cepac-model/>.

## Cohort weighting

For each country, we simulated four sub-cohorts of infants with varying risks of HIV exposure assessed at the time of birth:

1. infants with known, high-risk HIV exposure;
2. infants with known, low-risk HIV exposure;
3. infants with unrecognized HIV exposure; and
4. infants initially unexposed to HIV (but who may become exposed postnatally).

We used World Health Organization (WHO) criteria to define high-risk (i.e., mother was not on antiretroviral therapy [ART] in pregnancy, mother was on ART in pregnancy but had a viral load [VL] >1000 copies/mL near delivery, or mother was on ART for <4 weeks prior to delivery) and low-risk (i.e., mother was on ART and had VL <1000 copies/mL near delivery) exposure.^12^

Together, these four sub-cohorts include all infants born in a country. To estimate the proportion of infants within each sub-cohort, we used published data on country-specific estimates of maternal HIV prevalence/incidence during pregnancy, knowledge of HIV status, ART uptake, and probability of virologic suppression to populate the following equations:


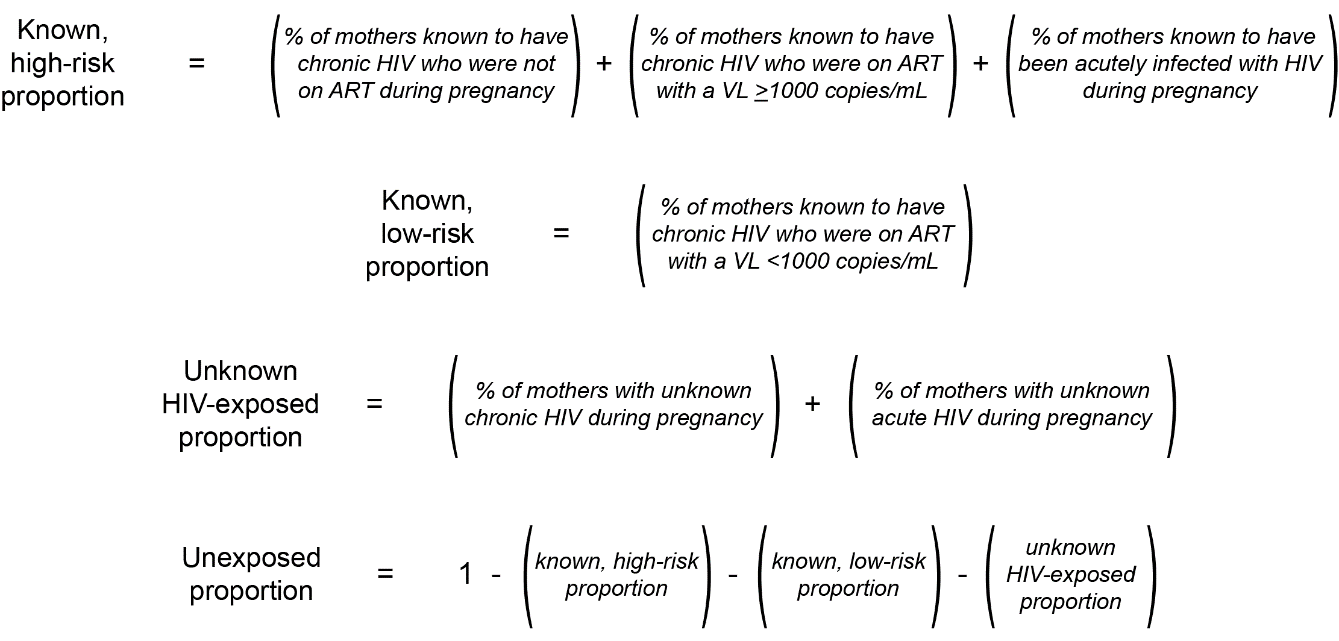


We used the proportion of infants within each of the four sub-cohorts to weight model outcomes and scale results representative of all infants born in a country.

## Additional modeled strategies

To determine the potential impact of broadly neutralizing antibody (bNAb) infant prophylaxis, we modeled the standard-of-care alone and various strategies offering bNAbs, in addition to the standard-of-care, to subsets of infants. A bNAb strategy targeted one of three nested populations of infants: 1) infants with known, high-risk HIV exposure at birth (*HR-HIVE* strategies); 2) infants with any known HIV exposure at birth (*HIVE* strategies); or 3) all infants regardless of known HIV exposure (*ALL* strategies). Infants in the target population were offered one of three dosing approaches: 1) one bNAbs dose at birth (*1-dose*), 2) one bNAbs dose at birth and one dose at three months (*2-doses*), or 3) one dose of bNAbs at birth and one bNAbs dose every three months thereafter for up to 18 months while breastfeeding (*Extended*). The combination of a target population and a dosing approach comprised a bNAb strategy. For example, *ALL–1-dose* would be the bNAb strategy in which all infants, regardless of known HIV exposure, are offered one dose of bNAbs at birth.

In the primary analysis, we mainly examined strategies in which all infants in the target population received the same dosing approach. However, we also examined hybrid strategies in which subsets of the target population received different dosing approaches. For example, *ALL–1-dose plus HIVE–Extended* would offer one dose of bNAbs to all infants at birth and an additional dose of bNAbs to infants with known HIV exposure every three months for up to 18 months while breastfeeding. With the exception of *ALL–1-dose plus HIVE–Extended* all other hybrid strategies did not hold comparable value to non-hybrid strategies and are, thus, only presented in the appendix (Supplementary Table 5).

## Infant HIV prophylaxis model structure

The infant HIV prophylaxis module (Supplemental Figure 2) is embedded within the CEPAC-P model and allows the user to simulate up to four concurrent and independent lines of HIV prophylaxis. The influence of infant HIV prophylaxis on reducing intrapartum (IP) transmission is reflected as a one-time multiplier on the IP component of the overall intrauterine (IU)/IP transmission risks at birth. For all months after birth, children who are breastfeeding, have not had a prior positive HIV test, and who do not have an active dose of prophylaxis from a prior month face optional user-specified prophylaxis eligibility criteria based on their age, maternal characteristics, and early infant diagnosis (EID) test results.

If the child meets eligibility criteria for that line of prophylaxis, they then face a probability of access and adherence to prophylaxis each month. If the child receives a dose of prophylaxis, an “efficacy multiplier” is applied to reduce postnatal transmission for a user-specified number of months during which the prophylaxis dose is active. Irrespective of efficacy, if a child receives prophylaxis in a given month, they also experience a probability of mild and/or severe drug toxicity in the month the dose was administered. Every toxicity event also carries a toxicity cost; when a severe toxicity is encountered, it also carries a probability of death in that month and triggers the prophylaxis regimen to be permanently stopped.

## Oral infant prophylaxis regimens and efficacy

In the standard-of-care and in all bNAb strategies, infants with known HIV exposure were eligible for oral infant prophylaxis per WHO recommendations (i.e., six weeks of daily nevirapine [NVP] for infants who are low-risk, HIV-exposed and 12 weeks of daily zidovudine [ZDV] plus nevirapine for infants who are high-risk, HIV-exposed).^12^ With conflicting data on the efficacy of one vs. multi-drug infant prophylaxis regimens, we modeled the same relative efficacy of both regimens.^13^

Modeled perinatal transmission risks among women on ART in pregnancy were largely derived from studies in which infants received short courses of antiretroviral prophylaxis after birth. Therefore, we did not model additional reductions in perinatal transmission among infants born to mothers on ART in pregnancy. However, among infants born to women who did not receive ART in pregnancy, we modeled a 69% relative reduction in the intrapartum component of perinatal transmission with oral infant prophylaxis based on data from the Post-Exposure Prophylaxis of Infants (PEPI) trial.^14^ In the PEPI study, the cumulative HIV acquisition rate at six weeks among breastfeeding infants who received a single NVP dose plus one week of ZDV was 5·10% compared to 1·58% among infants who received control plus extended dual prophylaxis (NVP + ZDV) (relative risk [RR]: 0·31).^14^ While transmission by six weeks may include some early postnatal transmission, we applied this risk reduction to the intrapartum component of vertical transmission risks for high-risk infants in the model, to be conservative with respect to the relative incremental benefits of bNAbs.

We also modeled a 71% decrease in postnatal transmission risk with oral infant prophylaxis based on an analysis of pooled individual data from five randomized trials of infant NVP prophylaxis demonstrating an adjusted hazard ratio (in time-varying analyses) of 0·29 (95% CI: 0·20-0·42) for vertical transmission while NVP was administered.^15^

## BNAb efficacy and duration

Anti-HIV bNAbs, such as VRC01, VRC01-LS, and VRC07-523LS, have demonstrated safety, tolerability, and favorable pharmacokinetics in infants.^16–18^ Current bNAbs under investigation for use as infant HIV prophylaxis include:

| **Study** | **BNAb(s) investigated** | **Target site** | **Trial type** | **Included populations** | **Trial status** |
| --- | --- | --- | --- | --- | --- |
| IMPAACT P1112 | VRC01  VRC01LS  VRC07-523LS | CD4-binding site  CD4-binding site  CD4-binding site | Phase 1 study on safety and pharamacokinetics | Infants with HIV exposure | Completed |
| PedMAB | VRC07-523LS  *plus*  CAP256V2LS | CD4-binding site  V1/V2 loop | Phase 1/2 study on safety, pharamacokinetics, and dose finding | Newborns with HIV exposure | Enrolling |
| SAMBULELO | VRC07-523LS | CD4-binding site | Phase 2 study on safety and pharmacokinetics | Newborns with HIV exposure, without HIV exposure, and with HIV | In development |
| EDCTP Neo bNAb Trial | VRC07-523LS | CD4-binding site | Proof-of-concept on effectiveness, efficacy, and operational feasibility | Newborns with HIV exposure | In development |
| IMPAACT 2037 | PGT121.414.LS *with* or *without* VRC07523-LS | V3 glycan  CD4-binding site | Phase 1 study on safety and pharmacokinetics | Newborns with HIV exposure | In development |

Table adapted from ^19^.

Since there are currently no published human infant HIV prophylaxis efficacy studies, base case efficacy was estimated using data from human adult, non-human primate, and *in vitro* studies. In the Antibody Mediated Prevention (AMP) trials, VRC01 did not prevent overall sexual acquisition of HIV among populations of cisgender men and transgender adults in the Americas and Europe, or among women in sub-Saharan Africa.^20^ However, acquisition of VRC01-sensitive isolates was 75·4% lower among individuals who received VRC01 compared to individuals who received placebo (IC_80_ <1 µg/mL).^20^ Other bNAbs under investigation, such as 3BNC117-LS, VRC07-523LS, 1-18, and 10-1074-LS are more potent and have greater breadth than VRC01.^21^ For example, VRC07-523LS neutralized 91% of primary African isolates in a multiclade panel with an IC_80_ of 4·4 µg/mL compared to VRC01 neutralizing 66% with an IC_80_ of 18·4 µg/mL.^21^

By applying the 75% efficacy against acquisition of sensitive isolates found in the AMP trial to the 91% neutralization coverage of VRC07-523LS found in the multiclade panel, we estimate VRC07-523LS could potentially have 68% overall efficacy. Similar to combination ART, a final bNAb infant prophylaxis product will likely be a combination of bNAbs, such as the VRC07-523LS+PGT121+PGDM1400 combination currently being studied as adult prophylaxis (NCT03205917, NCT03928821, NCT04212091, and NCT03721510), to achieve high breadth and potency. As such, we assumed a three-bNAb combination would have 70% efficacy in the base case, only slightly higher than the implied efficacy of VRC07-523LS alone to be conservative of the clinical impact a bNAb combination could have as prophylaxis. Given the uncertainty in final product characteristics, bNAb efficacy was varied widely in sensitivity analyses.

While the AMP studies investigated bNAb efficacy when delivered as pre-exposure prophylaxis, non-human primate studies have also observed efficacy of bNAbs as post-exposure prophylaxis if given within 24-30 hours of exposure.^22,23^ Based on these data, we assumed that a bNAb given at birth would reduce the risk of IP HIV transmission as post-exposure prophylaxis, in addition to acting as pre-exposure prophylaxis for postnatal transmission.

Pharmacokinetic data from HIV-exposed infants suggest that with an 80mg dose of VRC07-523LS delivered subcutaneously at birth, concentrations of the bNAb would remain sufficiently high to achieve protective efficacy through at least 12 weeks of life.^16^ In the base case, we assumed a three-month effect duration following each bNAb dose, and we also varied this assumption in sensitivity analyses.

## BNAb cost estimates

The base case average bNAb cost was modeled as $20 per dose, including the estimated costs of production (assuming a 100mg bNAb dose), as well as delivery (including training, personal protective equipment, and cold-chain), personnel, and facility/overhead needed to administer vaccines in low- and middle-income countries. The midpoint of the estimated cost range for all components was rounded to the nearest dollar and summed to reflect the total cost of bNAbs if they were produced, delivered, and administered at scale.

**Itemized costs included in modeled bNAb cost per dose**

| **Cost component** | **Cost estimate or range*** | **Rounded midpoint cost*** |
| --- | --- | --- |
| BNAb production costs per 100mg dose^24^ | $2·00 - $20·00 | $11 |
| Supply and delivery costs per dose, including: training, social mobilization, hand hygiene, personal protective equipment, waste management, and cold-chain costs^25^ | $1·66 | $2 |
| Service personnel costs^26^ | $0·68 - $1·45 | $1 |
| Facility overhead and capital costs^27^ | $1·92 - $4·21 | $3 |
| **Total estimated cost** | **$6**·**26 - $27**·**32** | **$17** |

*All costs are reported in 2020 USD.

To conservatively estimate the cost-effectiveness of bNAb infant prophylaxis, the total estimated cost was rounded up to the nearest ten ($20/dose). The bNAb cost was varied widely in sensitivity analyses ($5/dose - $100/dose) to capture the total estimated cost using the lower bound of each cost component and to account for scenarios in which there are additional unforeseen costs or if bNAbs are not produced at scale.

## Determining cost-effectiveness

We calculated incremental cost-effectiveness ratios (ICERs; USD/year of life saved [YLS]) by ordering strategies by increasing discounted life expectancy and dividing the difference in discounted costs by the difference in discounted life expectancy of consecutive, non-dominated strategies. Life expectancy and costs were discounted at 3%/year per standard cost-effectiveness practices.^28^ BNAb strategies were dominated if they resulted in shorter life expectancy than a less costly strategy or resulted in higher costs per YLS (i.e., higher ICER) than the next best-performing, non-dominated strategy. We considered a bNAb strategy cost-saving if it was non-dominated and resulted in the same or greater life expectancy than the standard-of-care at lower lifetime HIV-related costs.

While the WHO-CHOICE 100% GDP per capita-based cost-effectiveness threshold has been widely cited, there is growing concern that spending to this threshold may not offer good value, particularly in resource-limited settings.^29–35^ Due to budget constraints, investing in interventions at this GDP-based cost-effectiveness threshold may result in substantial opportunity costs (e.g., forgone health benefits) relative to other investments in health care that offer better value.^29–35^ Cost-effectiveness thresholds accounting for these additional considerations have been estimated in the published literature:

### **Cost-effectiveness thresholds for LMICs proposed in published literature**

| **Source** | **Cost-effectiveness threshold (in % GDP per capita)** | | | |
| --- | --- | --- | --- | --- |
|  | **Côte d’Ivoire** | **South Africa** | **Zimbabwe** |  |
| Woods B, Revill P, Sculpher M, Claxton K, 2016^29^ | 4-52% | 17-69% | 1-32% |  |
| Ochalek JM, Lomas J, Claxton KP, 2018^33^ | 15-19% | 43-58% | 22-30% |  |
| Jit M, 2021^34^ | 30-40% | 30-40% | 30-40% |  |
| Edoka EP, Stacey NK, 2020^35^ | - | 53% | - |  |
| Meyer-Rath G, Rensburg C, Larson B, Jamieson L, Rosen S, 2017^31^ | - | 10-17% | - |  |

Table adapted from ^36^.

As such, we used a more conservative cost-effectiveness threshold of 50% of GDP per capita in our base case analysis. Strategies that resulted in the greatest clinical benefit with an ICER <50% of a country’s GDP per capita (Côte d’Ivoire: $1163/YLS, South Africa: $2828/YLS, Zimbabwe: $607/YLS)^37^ were considered cost-effective. Given that a 50% GDP per capita cost-effectiveness threshold may still require donor-support, we also assessed the impact of using a 20% GDP per capita cost-effectiveness threshold in a sensitivity analysis. Since the cost-effective bNAb strategy did not change using the 20% GDP per capita cost-effectiveness threshold in any of the three settings modeled under base case bNAb cost and efficacy assumptions, we chose to only present the 50% GDP per capita cost-effectiveness threshold in the main manuscript.

# **Supplementary Figure 1.** CEPAC-P infant prophylaxis module flowchart


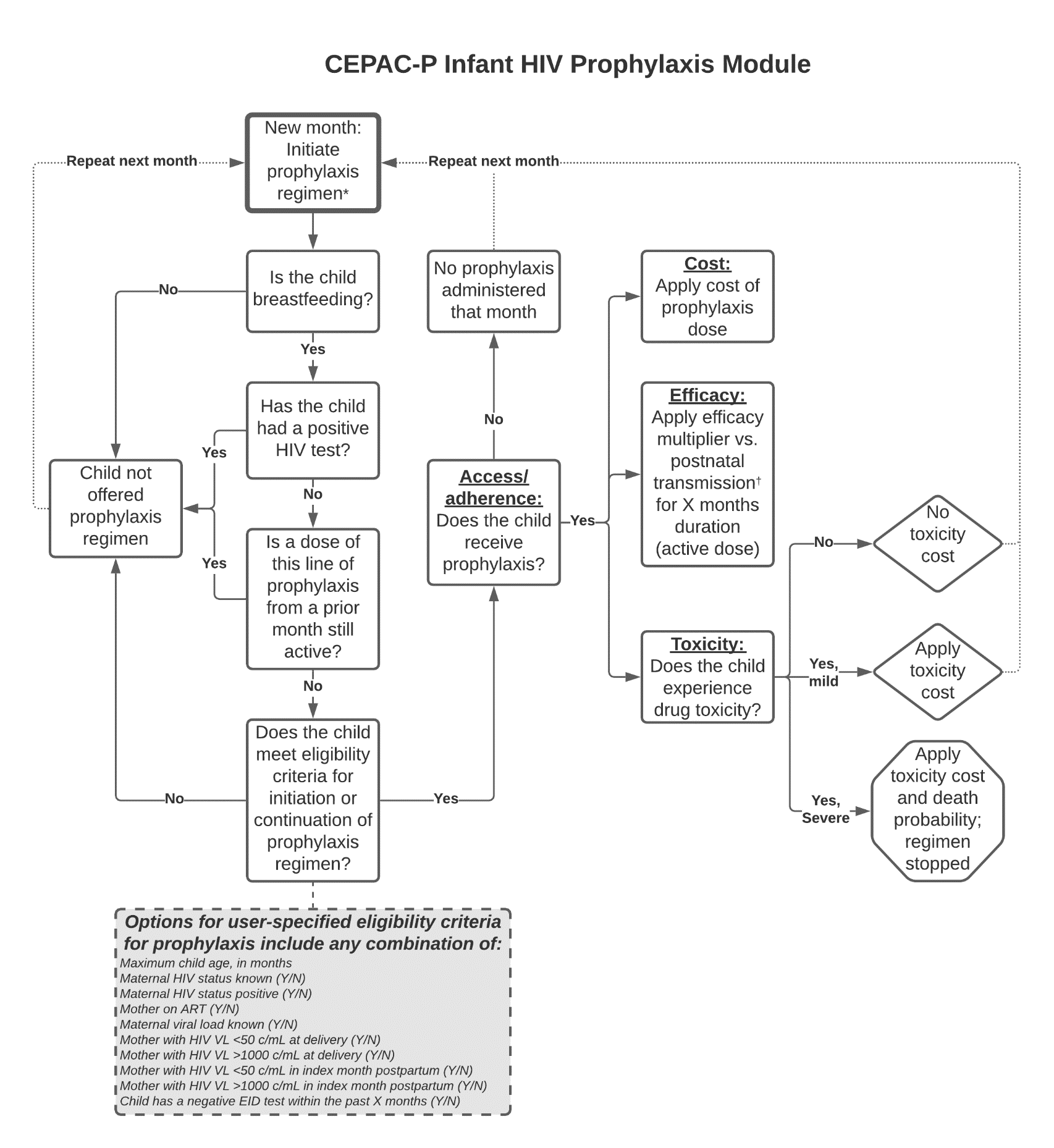
 CEPAC-P, Cost-Effectiveness of Preventing AIDS Complications–Pediatrics Model; Y, yes; N, no; ART, antiretroviral therapy; VL, viral load; EID, early infant diagnosis.

* This process is repeated separately for each line of prophylaxis each month.

† Efficacy of prophylaxis against intrapartum transmission is applied directly as a one-time multiplier against the intrapartum component of the intrauterine/intrapartum transmission risk.

# **Supplementary Figure 2.** Schematic of modeled cohorts and bNAb strategies


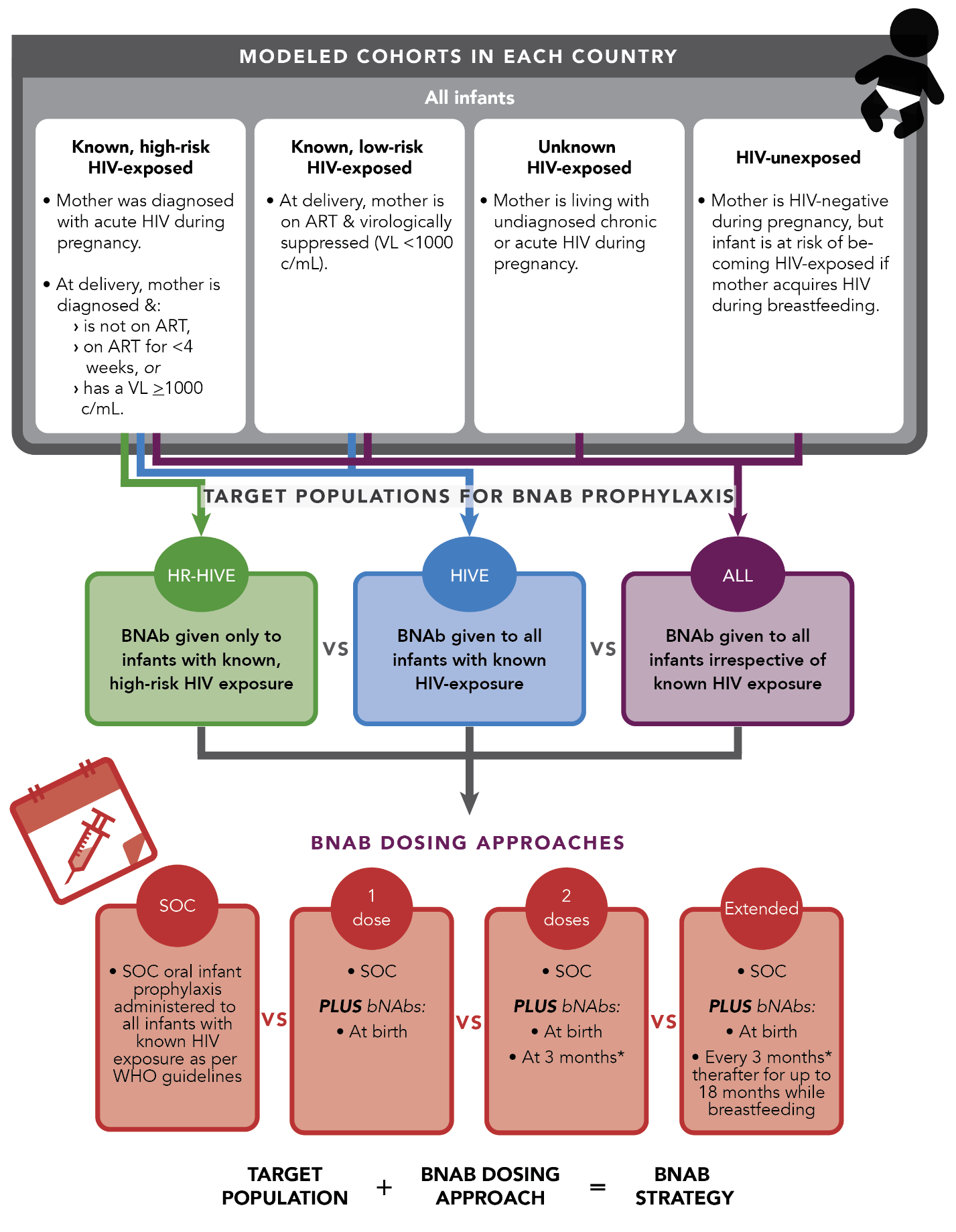


ART, antiretroviral therapy; VL, viral load; SOC, standard-of-care.

* The bNAb effect duration was varied during sensitivity analysis, and the frequency of administration was adjusted accordingly.

# **Supplementary Figure 3.** Efficiency frontier of all bNAb strategies assessed, by country


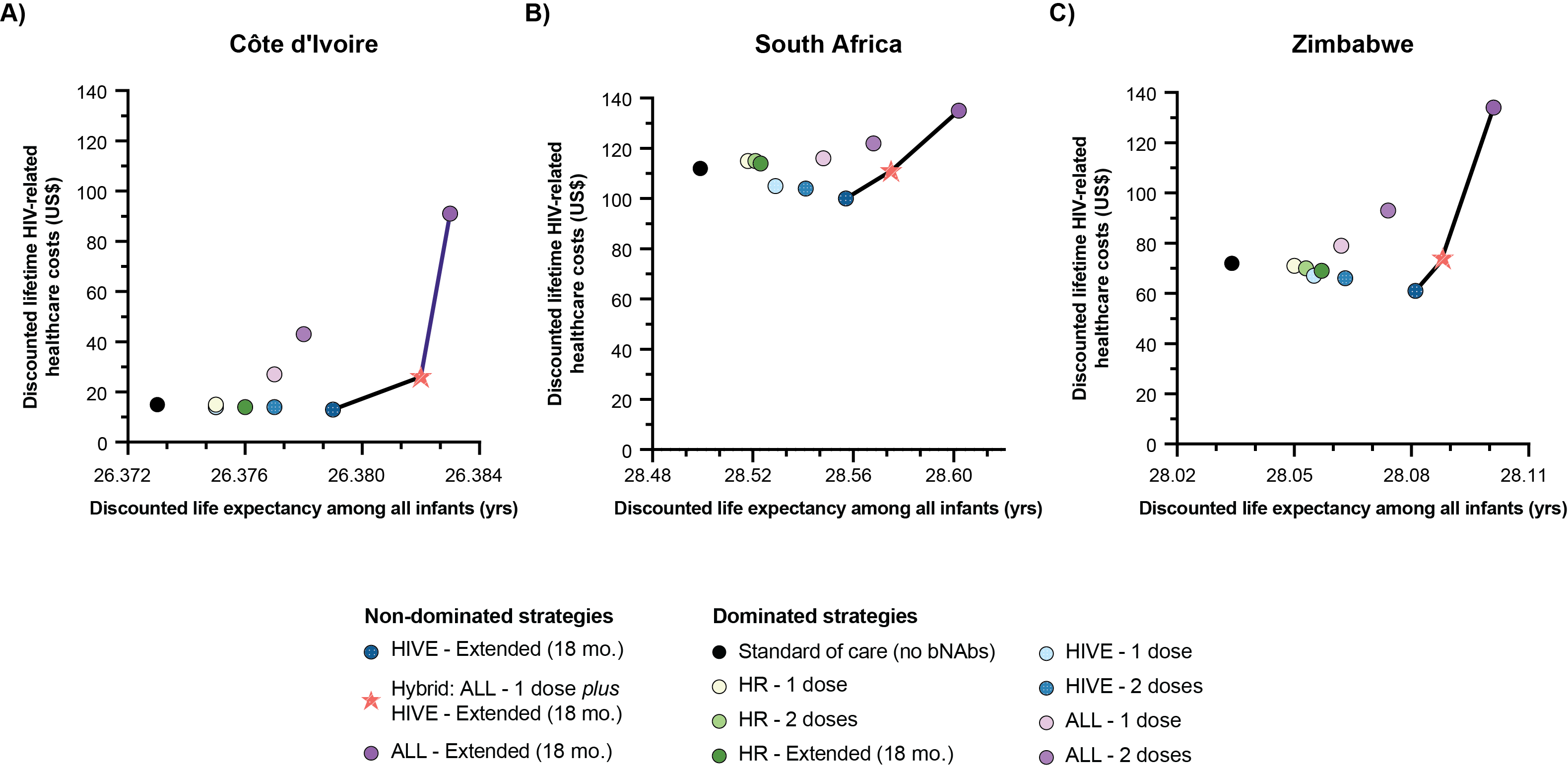


US$, United States dollar; yrs, years; HIVE, bNAb strategy offering bNAbs to infants with known HIV exposure at birth; ALL, bNAb strategy offering bNAbs to all infants regardless of HIV exposure; mo., month.

# **Supplementary Figure 4.** Cumulative vertical transmission in standard-of-care and bNAb strategies, by country


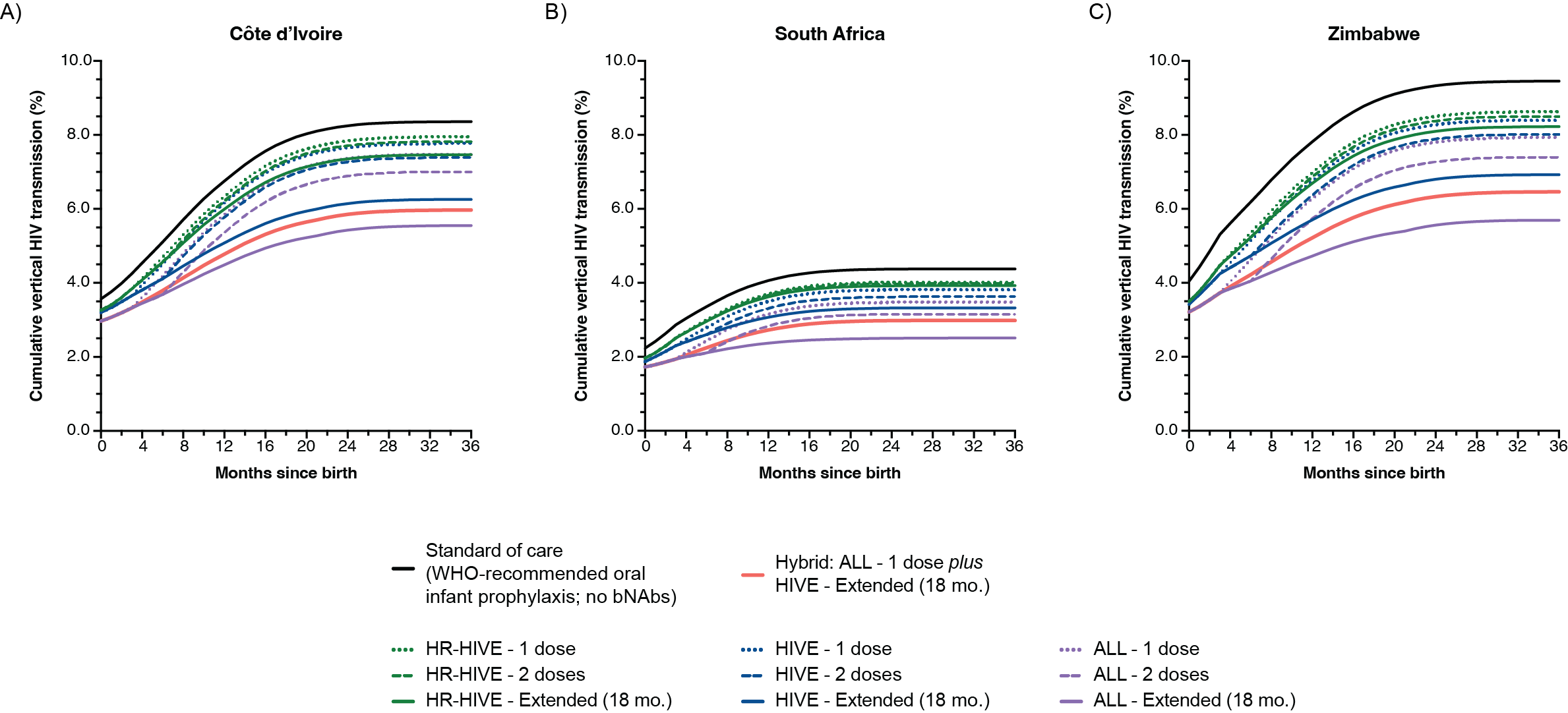


BNAb, broadly neutralizing antibody; WHO, World Health Organization; HR-HIVE, bNAb strategy targeting infants with known, high-risk HIV exposure at birth; HIVE, bNAb strategy targeting infants with known HIV exposure at birth; ALL, bNAb strategy targeting all infants regardless of known HIV exposure; mo., month.

# **Supplementary Figure 5.** Breakdown of intrauterine/intrapartum infections occurring in each exposure group


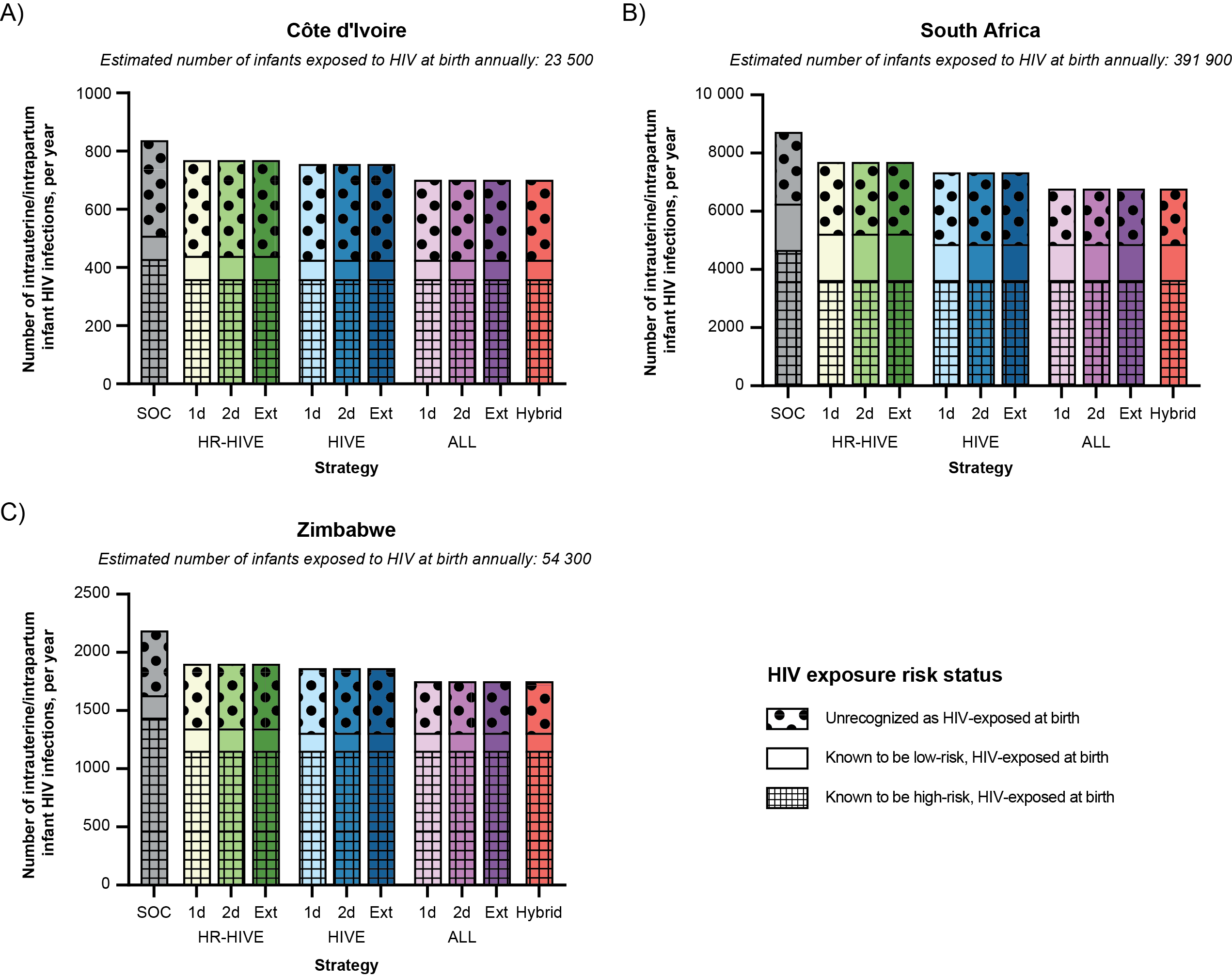


SOC, standard-of-care; 1d, one bNAb dose; 2d, two bNAb doses; Ext, extended bNAb dosing (one dose every three months through 18 months of life while breastfeeding); HR-HIVE, bNAb strategy targeting infants with known, high-risk HIV exposure at birth; HIVE, bNAb strategy targeting infants with known HIV exposure at birth; ALL, bNAb strategy targeting all infants regardless of known HIV exposure.

The estimated number of infants at risk of intrauterine/intrapartum HIV acquisition in each exposure group varies by country. In Côte d’Ivoire, an estimated 6900 infants have known, high-risk HIV exposure at birth; 14 800 infants have known, low-risk HIV exposure at birth; and 1400 infants have unrecognized HIV exposure at birth. In South Africa, an estimated 66 200 infants have known, high-risk HIV exposure at birth; 295 700 have known, low-risk HIV exposure at birth; and 11 600 infants have unrecognized HIV exposure at birth. In Zimbabwe, an estimated 12 600 infants have known, high-risk HIV exposure at birth; 35 500 infants have known, low-risk HIV exposure at birth; and 2600 infants have unrecognized HIV exposure at birth.

# **Supplementary Figure 6.** Cost-effective bNAb strategy across influential univariate sensitivity analyses, by country


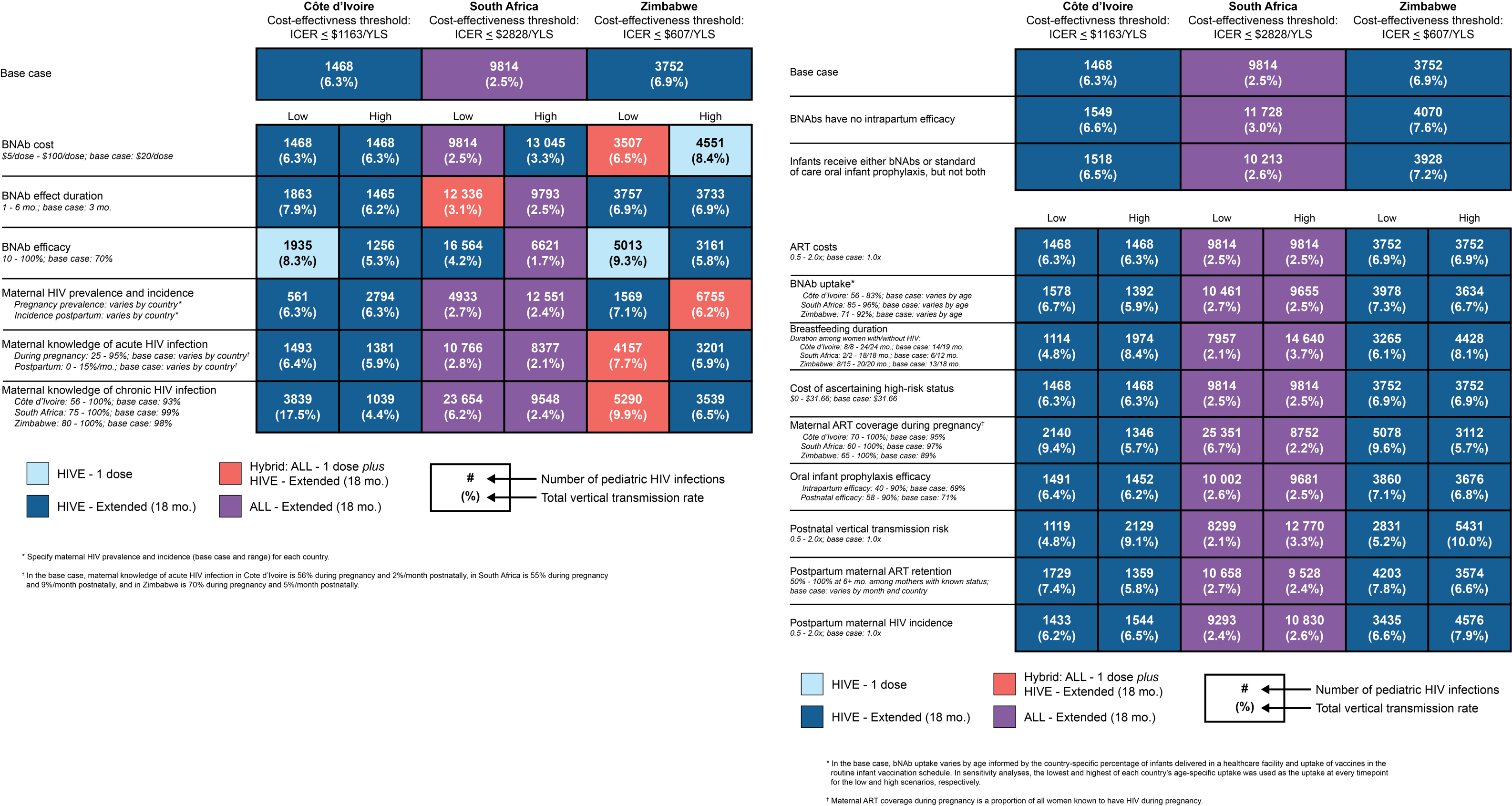


ICER, incremental cost-effectiveness ratio; YLS, year of life saved; bNAbs, broadly neutralizing antibodies; mo., month; HIVE, bNAb strategy targeting infants with known HIV exposure at birth; ALL, bNAb strategy targeting all infants regardless of known HIV exposure.

***** Maternal HIV prevalence at the time of delivery is inclusive of chronic HIV infection and incident infection during pregnancy. Maternal HIV prevalence was varied according to the lowest and highest subnational estimates in each country (Côte d’Ivoire: 1-5%, base case: 3%; South Africa: 15-45%, base case: 33%; and Zimbabwe: 5-25%, base case: 12%). Postpartum maternal HIV incidence was also decreased (Côte d’Ivoire: 0.001%/month, South Africa: 0.107%/month, Zimbabwe: 0.022%/month) or increased (Côte d’Ivoire: 0.005%/month, South Africa: 0.331%/month, Zimbabwe: 0.116%/month) proportional to the change in prevalence.

^†^ In the base case, maternal knowledge of acute HIV infection in Côte d’Ivoire is 56% for infection acquired during pregnancy and 2% per month postpartum, in South Africa is 55% during pregnancy and 9% per month postpartum, and in Zimbabwe is 70% during pregnancy and 5% per month postpartum.

# **Supplementary Figure 7.** Cost-effective bNAb strategy across non-influential univariate sensitivity analyses, by country


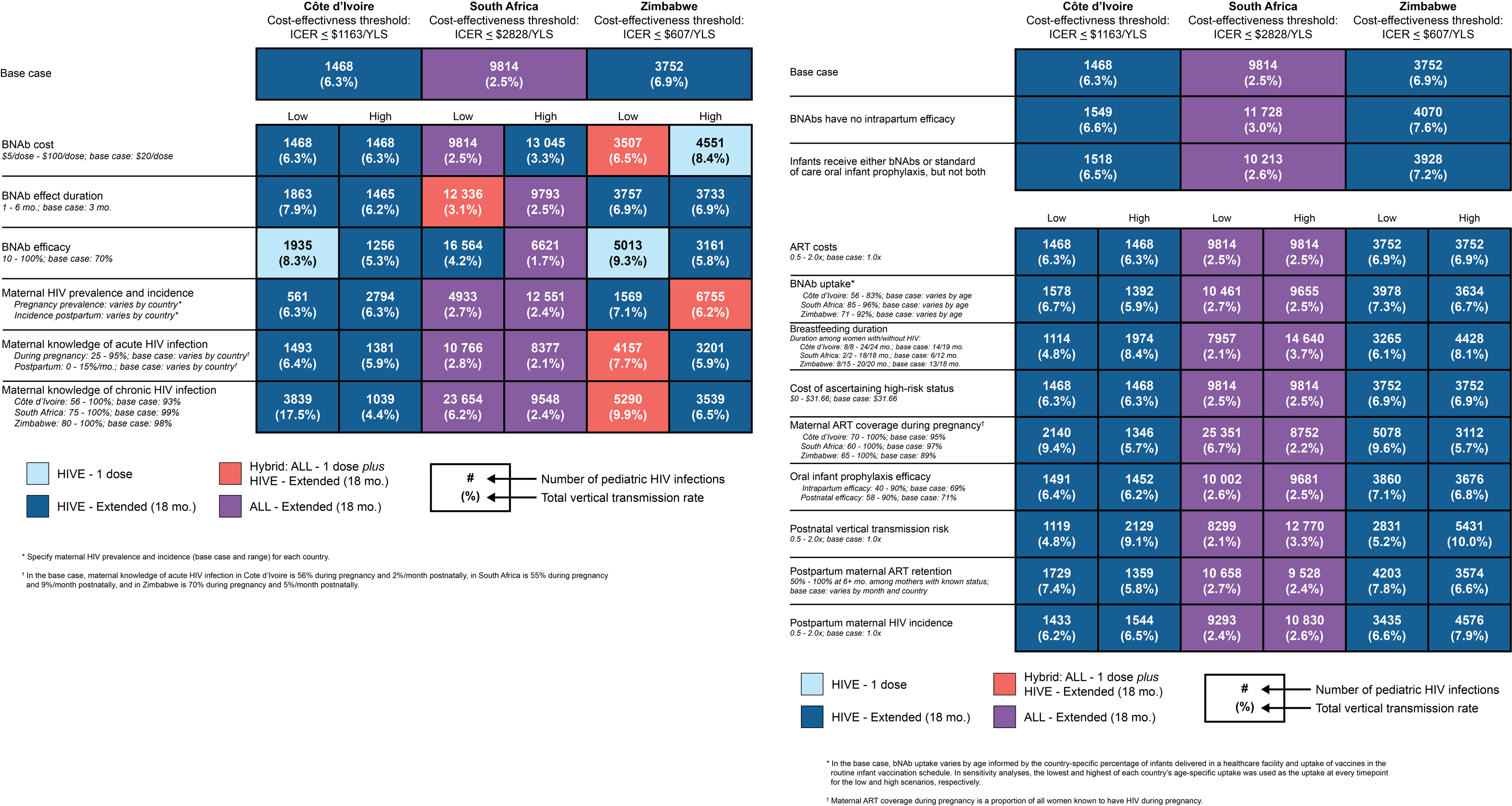


ICER, incremental cost-effectiveness ratio; YLS, year of life saved; bNAbs, broadly neutralizing antibodies; ART, antiretroviral therapy; mo., month; HIVE, bNAb strategy targeting infants with known HIV exposure at birth; ALL, bNAb strategy targeting all infants regardless of known HIV exposure.

* In the base case, bNAb uptake varies by age informed by the country-specific percentage of infants delivered in a healthcare facility and uptake of vaccines in the routine infant vaccination schedule. In sensitivity analyses, the lowest and highest of each country’s age-specific uptake was used as the uptake at every timepoint for the low and high scenarios, respectively.

† Maternal ART coverage during pregnancy is a proportion of all women known to have HIV during pregnancy.

# **Supplementary Figure 8.** Cost-effectiveness results of a three-way sensitivity analysis assessing bNAb efficacy, cost, and cost-effectiveness threshold, by country


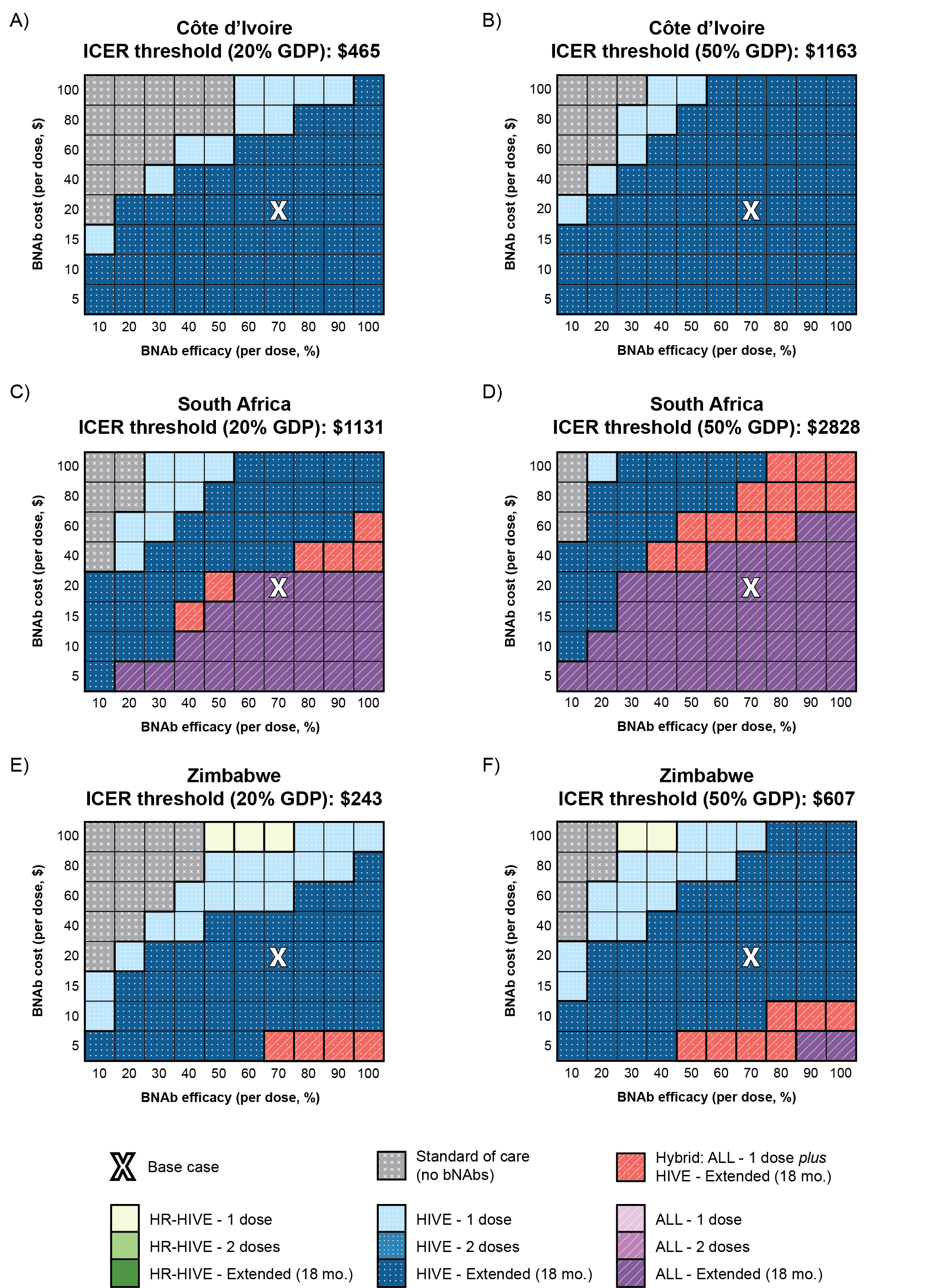


ICER, incremental cost-effectiveness ratio; GDP, gross domestic product; bNAb, broadly neutralizing antibody; HR-HIVE, bNAb strategy targeting infants with known, high-risk HIV exposure at birth; HIVE, bNAb strategy targeting infants with known HIV exposure at birth; ALL, bNAb strategy targeting all infants regardless of known HIV exposure; mo., month.

# **Supplementary Figure 9.** Cost-effectiveness results of a three-way sensitivity analysis assessing bNAb efficacy, cost, and efficacy duration, by country


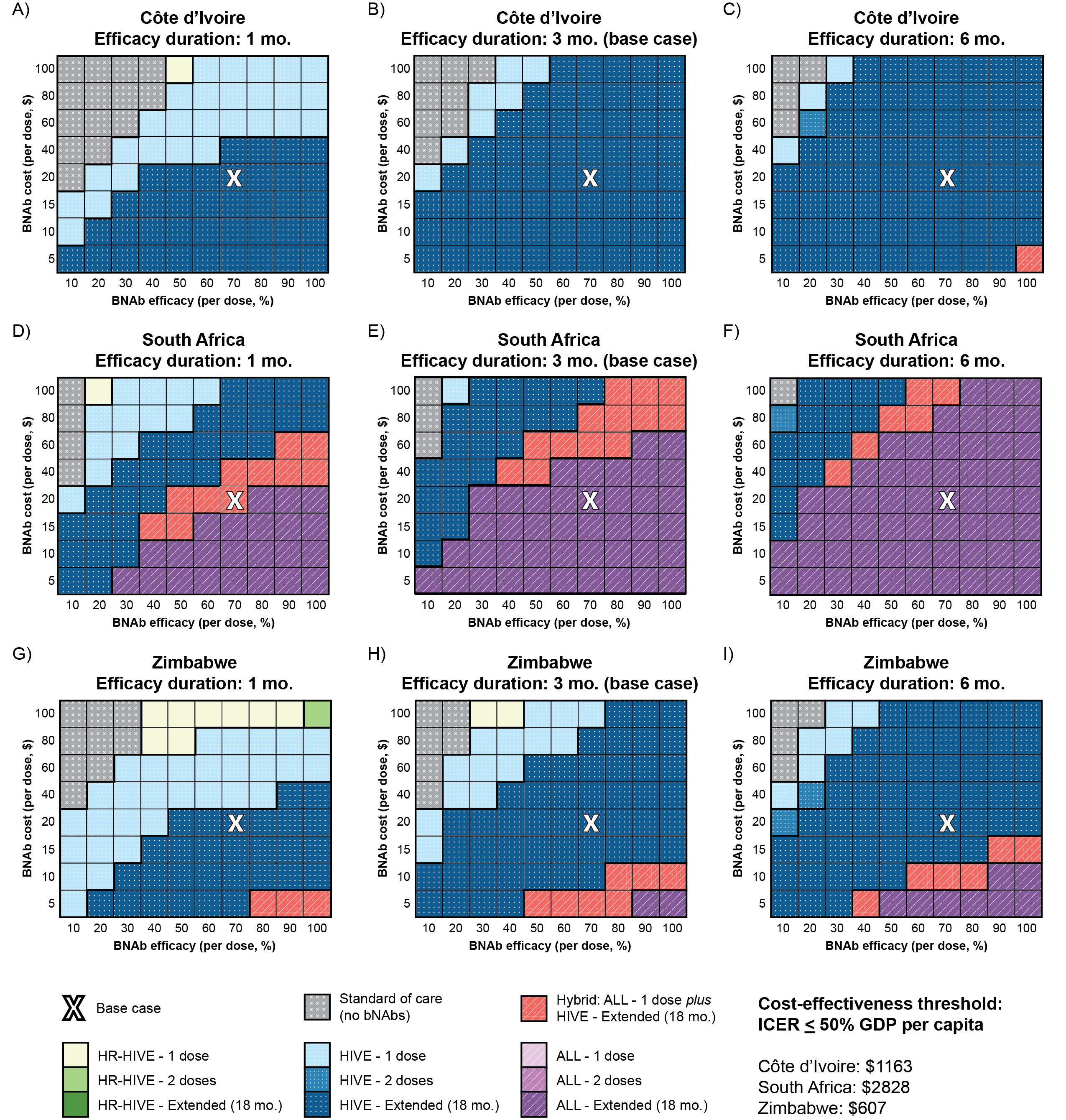


mo., month; bNAb, broadly neutralizing antibody; HR-HIVE, bNAb strategy targeting infants with known, high-risk HIV exposure at birth; HIVE, bNAb strategy targeting infants with known HIV exposure at birth; ALL, bNAb strategy targeting all infants regardless of known HIV exposure; ICER, incremental cost-effectiveness ratio; GDP, gross domestic product.

# **Supplementary Table 1.** Consolidated Health Economic Evaluation Reporting Standards (CHEERS) checklist

| **Topic** | **No.** | **Item** | **Location where item is reported** |
| --- | --- | --- | --- |
| **Title** |  |  |  |
| Title | 1 | Identify the study as an economic evaluation and specify the interventions being compared. | Title, Page 1 |
| **Abstract** |  |  |  |
| Abstract | 2 | Provide a structured summary that highlights context, key methods, results, and alternative analyses. | Abstract, Pages 4-5 |
| **Introduction** |  |  |  |
| Background and objectives | 3 | Give the context for the study, the study question, and its practical relevance for decision making in policy or practice. | Introduction, Pages 6-7 |
| **Methods** |  |  |  |
| Health economic analysis plan | 4 | Indicate whether a health economic analysis plan was developed and where available. | n/a |
| Study population | 5 | Describe characteristics of the study population (such as age range, demographics, socioeconomic, or clinical characteristics). | Methods, Third Paragraph |
| Setting and location | 6 | Provide relevant contextual information that may influence findings. | Methods, First Paragraph |
| Comparators | 7 | Describe the interventions or strategies being compared and why chosen. | Methods, Fourth Paragraph |
| Perspective | 8 | State the perspective(s) adopted by the study and why chosen. | Methods, Tenth Paragraph |
| Time horizon | 9 | State the time horizon for the study and why appropriate. | Methods, Tenth Paragraph |
| Discount rate | 10 | Report the discount rate(s) and reason chosen. | Methods, Tenth Paragraph |
| Selection of outcomes | 11 | Describe what outcomes were used as the measure(s) of benefit(s) and harm(s). | Methods, Tenth Paragraph |
| Measurement of outcomes | 12 | Describe how outcomes used to capture benefit(s) and harm(s) were measured. | n/a |
| Valuation of outcomes | 13 | Describe the population and methods used to measure and value outcomes. | n/a |
| Measurement and valuation of resources and costs | 14 | Describe how costs were valued. | Methods, Sixth and Ninth Paragraphs |
| Currency, price date, and conversion | 15 | Report the dates of the estimated resource quantities and unit costs, plus the currency and year of conversion. | Methods, Ninth Paragraph |
| Rationale and description of model | 16 | If modelling is used, describe in detail and why used. Report if the model is publicly available and where it can be accessed. | Methods, First paragraph; Appendix Supplementary Methods; Supplementary Figure 1 |
| Analytics and assumptions | 17 | Describe any methods for analysing or statistically transforming data, any extrapolation methods, and approaches for validating any model used. | Methods, Second Paragraph; Appendix Supplemental Methods; Supplementary Table 2 |
| Characterising heterogeneity | 18 | Describe any methods used for estimating how the results of the study vary for subgroups. | n/a |
| Characterising distributional effects | 19 | Describe how impacts are distributed across different individuals or adjustments made to reflect priority populations. | Methods , Third Paragraph |
| Characterising uncertainty | 20 | Describe methods to characterise any sources of uncertainty in the analysis. | Methods, Last Paragraph |
| Approach to engagement with patients and others affected by the study | 21 | Describe any approaches to engage patients or service recipients, the general public, communities, or stakeholders (such as clinicians or payers) in the design of the study. | n/a |
| **Results** |  |  |  |
| Study parameters | 22 | Report all analytic inputs (such as values, ranges, references) including uncertainty or distributional assumptions. | Table 1; Supplementary Table 2 |
| Summary of main results | 23 | Report the mean values for the main categories of costs and outcomes of interest and summarise them in the most appropriate overall measure. | Results, First and Second Paragraphs; Table 2 |
| Effect of uncertainty | 24 | Describe how uncertainty about analytic judgments, inputs, or projections affect findings. Report the effect of choice of discount rate and time horizon, if applicable. | Results, Third through Eighth Paragraphs; Figures 4-5; Supplementary Figures 5-7; Supplementary Tables 6-22 |
| Effect of engagement with patients and others affected by the study | 25 | Report on any difference patient/service recipient, general public, community, or stakeholder involvement made to the approach or findings of the study | n/a |
| **Discussion** |  |  |  |
| Study findings, limitations, generalisability, and current knowledge | 26 | Report key findings, limitations, ethical or equity considerations not captured, and how these could affect patients, policy, or practice. | Discussion, First through Ninth Paragraphs |
| **Other relevant information** |  |  |  |
| Source of funding | 27 | Describe how the study was funded and any role of the funder in the identification, design, conduct, and reporting of the analysis | Title, Pages 2-3 |
| Conflicts of interest | 28 | Report authors conflicts of interest according to journal or International Committee of Medical Journal Editors requirements. | Title, Page 3 |

# **Supplementary Table 2.** Extended model input parameters

| Parameter | Côte d’Ivoire | South Africa | Zimbabwe | Reference(s) |
| --- | --- | --- | --- | --- |
| Maternal characteristics |  |  |  |  |
| Proportion of mothers with chronic infection, % |  |  |  |  |
| High-risk cohort | 98 | 79 | 62 | Derived from ^38–55^ |
| Low-risk cohort | 100 | 100 | 100 | Assumption |
| Unrecognized exposed cohort | 94 | 12 | 27 | Derived from ^38–50,53–56^ |
| Unexposed cohort | 0 | 0 | 0 | Assumption |
| Probability of acute HIV infection in pregnancy, % | 0·03 | 3·30 | 1·94 | ^55,45–48^ |
| Probability of acute HIV infection postnatally, %/mo. | 0·003 | 0·240 | 0·056 | ^52,55,57–59^ |
| Probability of known HIV status in pregnancy, % |  |  |  | Derived from ^38–41,49,52,56,60,61^ |
| Among mothers with chronic infection | 93 | 99 | 98 | ^42,43,49,53,55,62^ |
| Among mothers with acute infection | 56 | 55 | 70 | ^38–44,56^ |
| Probability of learning HIV status postnatally, %/mo. | 2 | 9 | 5 | ^43,53,55^ |
| Probability of being on ART during pregnancy, % |  |  |  |  |
| High-risk cohort |  |  |  |  |
| Among those with chronic infection | 86 | 84 | 41 | Derived from ^42,43,49–55^ |
| Among those with acute infection | 95 | 97 | 89 | Derived from ^38–50,53–56,63^ |
| Low-risk cohort | 100 | 100 | 100 | Assumption |
| Unrecognized exposed and unexposed cohorts | 0 | 0 | 0 | Assumption |
| Viral load if on ART at delivery, % |  |  |  |  |
| Non-high-risk cohort |  |  |  | Derived from ^42,43,49–53,60,64^ |
| HIV RNA <50 c/mL | 76 | 76 | 76 |  |
| HIV RNA 50-1,000 c/mL | 24 | 24 | 24 |  |
| HIV RNA >1,000 c/mL | 0 | 0 | 0 |  |
| High-risk and unrecognized exposed cohorts |  |  |  | Assumption |
| HIV RNA >1,000 c/mL | 100 | 100 | 100 |  |
| Probability of delivering in a healthcare facility, % | 70 | 96 | 86 | ^42–44^ |
| HIV RNA <1,000 c/mL among postpartum women on ART, by month postpartum | 77-92 | 77-92 | 77-92 | ^65–74^ |
| Retention in HIV care, % |  |  |  | ^75^ |
| High-risk and low-risk cohorts |  |  |  |  |
| 6 mo. postpartum | 87 | 88 | 88 |  |
| 12 mo. postpartum | 87 | 87 | 87 |  |
| 24 mo. postpartum | 79 | 79 | 79 |  |
| Unrecognized exposed and unexposed cohorts |  |  |  |  |
| 6 mo. postpartum | 9 | 37 | 19 |  |
| 12 mo. postpartum | 17 | 57 | 33 |  |
| 24 mo. postpartum | 27 | 69 | 47 |  |
| Infant cohort characteristics and transmission risks |  |  |  |  |
| Total number of live births (thousands) | 907 | 1153 | 425 | ^76^ |
| Age, mean (SD), mo. | 0 (0) | 0 (0) | 0 (0) | Assumption |
| Proportion female, % | 52 | 52 | 52 | ^77^ |
| Initial CD4% at infection, mean (SD), % | 45 (9) | 45 (9) | 45 (9) | ^1^ |
| Breastfed infants, % |  |  |  | ^43,53,55,78^ |
| Unexposed or Unrecognized HIV-exposed infants | 99 | 84 | 99 |  |
| Infants with known HIV exposure | 99 | 66 | 94 |  |
| Breastfeeding duration, mean (SD) mo. |  |  |  | ^42–44,68,79^ |
| Unexposed or Unrecognized HIV-exposed infants | 19 (7) | 12 (6) | 18 (7) |  |
| Infants with known HIV exposure | 14 (7) | 6 (6) | 13 (7) |  |
| Intrauterine infections as a share of all perinatal infections, % | 67 | 67 | 67 | ^80^ |
| Perinatal transmission, one-time % |  |  |  |  |
| Chronic maternal HIV infection in pregnancy |  |  |  |  |
| On ART, HIV RNA <50 c/mL at delivery | 0·24 | 0·24 | 0·24 | ^81^ |
| On ART, HIV RNA 50-1,000 c/mL at delivery | 1·45 | 1·45 | 1·45 | ^81^ |
| On ART, HIV RNA >1,000 c/mL at delivery | 4·14 | 4·14 | 4·14 | ^81^ |
| Not on ART at delivery | 19·70 | 19·70 | 19·70 | ^82^ |
| Acute maternal HIV infection in pregnancy |  |  |  |  |
| On ART at delivery | 8·79 | 8·79 | 8·79 | ^81,82^ |
| Not on ART at delivery | 18·10 | 18·10 | 18·10 | ^82^ |
| Postnatal transmission rate, %/mo.^†, φ^ |  |  |  |  |
| On ART, HIV RNA <50 c/mL | 0·06 | 0·06 | 0·06 | ^81^ |
| On ART, HIV RNA 50-1,000 c/mL | 0·39 | 0·39 | 0·39 | ^81^ |
| On ART, HIV RNA >1,000 c/mL | 0·78 | 0·78 | 0·78 | ^81^ |
| Not on ART | 0·89 | 0·89 | 0·89 | ^82^ |
| Early infant diagnosis |  |  |  |  |
| Probability of uptake, % by age |  |  |  |  |
| Birth | 0 | 93 | 9 | ^83,84^ |
| 6-8 weeks | 69 | 64 | 54 | ^53,61,68,85–89^ |
| 6/9 months (country-specific timepoint) | 25 | 25 | 26 | ^53,90^ |
| 18 months | 22 | 22 | 22 | ^91^ |
| Nucleic acid amplification test characteristics |  |  |  |  |
| Sensitivity, 0 months after infection, % | 0 | 0 | 0 | Assumption |
| Sensitivity, >1 month after infection, % | 99·4 | 99·4 | 99·4 | ^92^ |
| Specificity, % | 99·6 | 99·6 | 99·6 | ^92^ |
| Probability of result return, % | 84 | 96 | 83 | ^61,93,94^ |
| Delay between primary test and result return, mean mo. (SD) | 2 (1) | 2 (1) | 2 (1) | ^61,93^ |
| Probability of linkage to care upon detection, % | 59·8 | 80·7 | 59·8 | ^95,96^ |
| Antibody test characteristics |  |  |  |  |
| Sensitivity, 0 months after infection, % | 0 | 0 | 0 | Assumption |
| Sensitivity, >1 month after infection, % | 100 | 100 | 100 | ^97,98^ |
| Specificity, % |  |  |  |  |
| Before seroconversion | 0·01 | 0·01 | 0·01 | Assumption |
| After seroconversion | 99 | 99 | 99 | ^98^ |
| Probability of result return, % | 99·6 | 99·5 | 99·7 | ^93,95^ |
| Delay between primary test and result return, mean mo. (SD) | 0 (0) | 0 (0) | 0 (0) | ^93^/Assumption |
| Probability of linkage to care upon detection, % | 92·6 | 92·6 | 92·6 | ^95^ |
| Probability of HIV detection after presenting to care with a severe OI, % | 100 | 100 | 100 | Assumption |
| Infant HIV prophylaxis |  |  |  |  |
| Probability of receiving scheduled prophylaxis, % |  |  |  |  |
| Standard-of-care oral infant prophylaxis (NVP +/- ZDV) ^‡^ | 86 | 86 | 86 | ^99^ |
| bNAb prophylaxis (varies by age)^§^ | 56-83 | 85-96 | 71-92 | ^42–44,100^ |
| Efficacy of standard-of-care oral infant prophylaxis (NVP +/- ZDV)^‡,¶^ % |  |  |  | ^14,15^ |
| Against intrapartum transmission | 69 | 69 | 69 |  |
| Against postnatal transmission | 71 | 71 | 71 |  |
| Efficacy of bNAb prophylaxis against intrapartum and postnatal transmission (in addition to efficacy of standard-of-care), % | 70 | 70 | 70 | Assumption based on ^20,21^ |
| Duration of bNAb effect, mo. | 3 | 3 | 3 | ^16,17^ |
| Probability of infant standard-of-care oral prophylaxis major toxicity, one-time % | 0 | 0 | 0 | Assumption based on ^101–103^ |
| Probability of infant bNAb prophylaxis minor toxicity, one-time % | 50 | 50 | 50 | Assumption |
| Probability of infant bNAb prophylaxis major toxicity, one-time % | 0 | 0 | 0 | Assumption |
| Natural history |  |  |  |  |
| Monthly CD4% or CD4 decline in absence of ART, by age |  |  |  |  |
| <5 years (range by age and time of transmission), % | 0·5 – 4·0 | 0·5 – 4·0 | 0·5 – 4·0 | ^1^ |
| ≥5 years, mean (SD) (range by viral load and CD4), cells/mm^3^ | 3·0 (0·3) – 6·4 (0·3) | 3·0 (0·3) – 6·4 (0·3) | 3·0 (0·3) – 6·4 (0·3) | ^1,104^ |
| Monthly risk of clinical events, by age, % |  |  |  |  |
| 0-5 years (range by age and CD4%) |  |  |  | ^77^ |
| WHO stage 3 event (except tuberculosis) | 3·3 – 11·6 | 3·3 – 11·6 | 3·3 – 11·6 |  |
| WHO stage 4 event (except tuberculosis) | 1·4 – 6·4 | 1·4 – 6·4 | 1·4 – 6·4 |  |
| Tuberculosis (any body site) | 0·5 – 3·8 | 0·5 – 3·8 | 0·5 – 3·8 |  |
| ≥5 years (range by CD4) |  |  |  | ^105,106^ |
| Mild fungal infection | 0·75 – 9·42 | 1·76 – 3·14 | 1·8 – 3·1 |  |
| Mild bacterial infection | 0·93 – 2·01 | 0 | 0 |  |
| Bacterial gastroenteritis | 0·18 – 1·33 | 0 | 0 |  |
| Malaria | 0·85 – 2·97 | 0 | 0 |  |
| Visceral bacterial infection | 0·15 – 1·70 | 0·04 – 0·71 | 0·04 – 0·71 |  |
| WHO stage 3 or 4 visceral disease | 0·03 – 2·92 | 0·03 – 1·43 | 0·03 – 1·43 |  |
| WHO stage 3 or 4 mucocutaneous disease | 0·01 – 2·74 | 0·03 – 2·26 | 0·03 – 2·26 |  |
| Other WHO stage 3 or 4 disease | 0·04 – 2·08 | 0·02 – 0·73 | 0·02 – 0·73 |  |
| Other severe disease | 0·75 – 3·79 | 0·19 – 1·67 | 0·19 – 1·67 |  |
| Other mild infection | 0·72 – 4·16 | 2·39 | 2·39 |  |
| Tuberculosis (any body site) | 0·06 – 0·66 | 0·03 – 1·74 | 0·03 – 1·74 |  |
| Risk of death within 30 days of clinical event, by age, % |  |  |  |  |
| 0-5 years |  |  |  | ^1,77^ |
| WHO stage 3 or 4 event | 13·5 | 13·5 | 13·5 |  |
| Tuberculosis | 11·1 | 11·1 | 11·1 |  |
| ≥5 years (range by CD4) |  |  |  | ^105,106^ |
| Mild fungal infection | 0 | 0 | 0 |  |
| Mild bacterial infection | 0 | ... | ... |  |
| Bacterial gastroenteritis | 0 | ... | ... |  |
| Malaria | 0 – 16·7 | ... | ... |  |
| Visceral bacterial infection | 0 – 16·7 | 0 | 0 |  |
| WHO stage 3 or 4 visceral disease | 6·7 – 12·3 | 0·5 | 0·5 |  |
| WHO stage 3 or 4 mucocutaneous disease | 0 | 0 | 0 |  |
| Other WHO stage 3 or 4 disease | 6·7 – 12·3 | 0 | 0 |  |
| Other severe disease | 0 – 16·7 | 0·4 | 0·4 |  |
| Other mild infection | 0 | 2·4 | 2·4 |  |
| Tuberculosis (any body site) | 6·5 – 50·0 | 9·2 | 9·2 |  |
| Monthly risk of HIV-related mortality, by age, % (range by CD4 and OI history) |  |  |  | ^1,77,105,106^ |
| 0-5 months | 5·3 – 40·8 | 5·3 – 40·8 | 5·3 – 40·8 |  |
| 6-11 months | 2·2 – 16·8 | 2·2 – 16·8 | 2·2 – 16·8 |  |
| 12-23 months | 0·9 – 7·2 | 0·9 – 7·2 | 0·9 – 7·2 |  |
| 24-35 months | 0·5 – 3·6 | 0·5 – 3·6 | 0·5 – 3·6 |  |
| 36-47 months | 0·4 – 2·9 | 0·4 – 2·9 | 0·4 – 2·9 |  |
| 48-59 months | 0·1 – 1·0 | 0·1 – 1·0 | 0·1 – 1·0 |  |
| 5-13 years | 0·1 – 1·0 | 0·1 – 1·0 | 0·1 – 1·0 |  |
| ≥13 years | 0·04 – 5·4 | 0·2 – 9·5 | 0·2 – 9·5 |  |
| Monthly risk of non-HIV related mortality, % |  |  |  |  |
| HIV-exposed, uninfected children, born to mothers not on ART, by age |  |  |  | ^107,108^ |
| 0-2 months | 1·01 | 1·01 | 1·01 |  |
| 3-5 months | 0·41 | 0·41 | 0·41 |  |
| 6-11 months | 0·28 | 0·28 | 0·28 |  |
| 12-17 months | 0·14 | 0·14 | 0·14 |  |
| 18-23 months | 0·07 | 0·07 | 0·07 |  |
| ≥24 months | See below | See below | See below |  |
| All others, by age |  |  |  | ^109,110^ |
| 0-11 months (range by age and sex) | 0·50 – 0·66 | 0·24 – 0·31 | 0·30 – 0·38 |  |
| 12-23 months (range by sex) | 0·05 – 0·06 | 0·02 | 0·03 |  |
| 24-59 months (range by sex) | 0·05 – 0·06 | 0·02 | 0·03 |  |
| 5-13 years (range by age and sex) | 0·02 – 0·03 | 0·003 – 0·006 | 0·01 – 0·02 |  |
| 13-99 years | 0·02 – 2·40 | 0·004 – 1·930 | 0·01 – 1·89 |  |
| 100 years | 100 | 100 | 100 |  |
| Treatment |  |  |  |  |
| Efficacy of adult OI prophylaxis (co-trimoxazole) (range by OI type), % | 18 – 88 | 18 – 50 | 18 – 88 | ^106,111^ |
| Probability of adult OI prophylaxis (co-trimoxazole) minor toxicity, one-time % | 17 | 17 | 17 | ^106,111^ |
| Probability of adult OI prophylaxis (co-trimoxazole) major toxicity, one-time % | 6 | 6 | 6 | ^106,111^ |
| Probability of virologic suppression (<1,000 c/mL for children, <50 c/mL for adults) at 48 weeks while on ART, one-time % |  |  |  |  |
| Pediatric 1^st^ line ART (LPV/r or EFV-based) | 76 | 76 | 76 | ^112–118^ |
| Pediatric 2^nd^ and subsequent lines of ART (DTG-based) | 87 | 87 | 87 | ^119^ |
| Adult 1^st^ line ART (DTG-based) | 90 | 90 | 90 | ^120,121^ |
| Adult 2^nd^ and subsequent lines of ART (PI-based) | 83 | 83 | 83 | ^122^ |
| Probability of virologic failure after initial suppression, %/mo. |  |  |  |  |
| Pediatric 1^st^ line ART | 0·52 | 0·52 | 0·52 | ^113,116^ |
| Pediatric 2^nd^ and subsequent lines of ART | 0·37 | 0·37 | 0·37 | ^119^ |
| Adult 1^st^ line ART | 0·60 | 0·60 | 0·60 | ^120,121,123,124^ |
| Adult 2^nd^ and subsequent lines of ART | 0·20 | 0·20 | 0·20 | ^125^ |
| Probability of resuppression when reinitiating ART, one-time % |  |  |  |  |
| Pediatric ART lines | 31 | 31 | 31 | ^126^ |
| Adult ART lines | 67 | 67 | 67 | ^127,128^ |
| Effect of ART on CD4 when suppressed, by age |  |  |  |  |
| 0-4 years, absolute CD4% gain/mo., mean (SD) |  |  |  |  |
| 0-6 months after ART initiation | 2·2 (0·6) | 2·2 (0·6) | 2·2 (0·6) | ^129,130^ |
| ≥6 months after ART initiation | 0·7 (0·2) | 0·7 (0·2) | 0·7 (0·2) | ^129,130^ |
| ≥5 years, absolute CD4 gain/mo., mean (SD) |  |  |  |  |
| 0-2 months after ART initiation, cells/mm^3^ | 83·2 (38·2) | 83·2 (38·2) | 83·2 (38·2) | ^131^ |
| ≥2 months after ART initiation, cells/mm^3^ | 4·2 (1·9) | 4·2 (1·9) | 4·2 (1·9) | ^131^ |
| Relative risk reduction of HIV-related mortality for patients on ART, by age, % |  |  |  |  |
| 0-5 years | 90 | 90 | 90 | ^1^ |
| 5-13 years | 90 | 90 | 90 | ^1^ |
| ≥13 years (range by viral load and CD4) | 55 – 96 | 55 – 96 | 55 – 96 | ^132^ |
| Relative risk reduction of clinical events for patients on ART, by age, % |  |  |  |  |
| 0-5 years | 85 | 85 | 85 | ^1^ |
| 5-13 years (range by OI type) | 0 – 85 | 0 – 85 | 0 – 85 | ^1^ |
| ≥13 years (range by OI type) | 0 – 32 | 0 – 32 | 0 – 32 | ^132^ |
| Loss to follow-up probability after ART initiation, by age, %/mo. |  |  |  |  |
| 0-13 years | 1·6 | 0·8 | 0·4 | ^133–142^ |
| ≥13 years | 1·0 | 2·1 | 1·1 | ^143–147^ |
| Return to care probability after 6 months of loss to follow-up, %/mo. |  |  |  |  |
| With presentation of a severe OI | 100 | 100 | 100 | Assumption |
| Without presentation of a severe OI | 1·3 | 1·3 | 1·3 | ^148^ |
| Frequency of CD4 monitoring while in care, test interval, mo. |  |  |  | ^80^ |
| First 12 months post-ART initiation | 6 | 6 | 6 |  |
| After 12 months post-ART initiation | 12 | 12 | 12 |  |
| CD4 threshold to stop CD4 monitoring, cells/mm^3^ | >350 | >350 | >350 |  |
| Frequency of viral load monitoring while in care, mo. |  |  |  | ^80^ |
| First 12 months post-ART initiation | 6 | 6 | 6 |  |
| After 12 months post-ART initiation | 12 | 12 | 12 |  |
| Costs (in 2020 USD) |  |  |  |  |
| Early infant diagnosis program, $/test |  |  |  |  |
| Nucleic acid amplification test | 26·06 | 26·06 | 26·06 | ^149^ |
| Antibody test | 4·06 | 4·06 | 4·06 | ^150^ |
| Negative result return | 2·09 | 2·09 | 2·09 | ^151^ |
| Positive result return | 3·48 | 3·48 | 3·48 |  |
| Pediatric ART (range by age and weight, per mo.) |  |  |  |  |
| 1^st^ line | 5·65 – 11·70 | 5·65 – 11·70 | 5·65 – 11·70 | ^152,153^ |
| 2^nd^ line | 12·43 – 25·99 | 12·43 – 25·99 | 12·43 – 25·99 | ^152,153^ |
| Adult ART (per mo.) |  |  |  |  |
| 1^st^ line | 5·50 | 5·50 | 5·50 | ^154^ |
| 2^nd^ line | 23·00 | 23·00 | 23·00 | ^154^ |
| Infant oral prophylaxis (NVP +/- ZDV), per mo. | 0·91 – 1·74 | 0·91 – 1·74 | 0·91 – 1·74 | ^152,153^ |
| bNAb prophylaxis, per dose | 20·00 | 20·00 | 20·00 | ^24–27^ |
| Routine HIV care, by age, $/mo. (range by CD4%/CD4 and ART status) |  |  |  |  |
| 0-18 years | 4·59 – 181·90 | 3·73 – 148·88 | 7·04 – 35·18 | ^105,155–158^ |
| ≥18 years | 4·16 – 28·48 | 3·73 – 148·88 | 7·04 – 35·18 | ^105,106,156–158^ |
| Routine CD4 monitoring test, $/test | 3·98 | 3·98 | 3·98 | ^159,160^ |
| Routine HIV viral load test, $/test | 24·05 | 24·05 | 24·05 | ^149^ |
| Adult OI prophylaxis (co-trimoxazole), $/mo. | 1·04 | 1·04 | 1·04 | ^161^ |
| Acute OI care (range by OI type), $/mo. |  |  |  |  |
| 0-5 years | 128·96 – 456·65 | 867·99 – 1525·15 | 0 | ^155,162,163^ |
| ≥5 years | 82·51 – 574·89 | 230·20 – 780·51 | 0 | ^105,106,158^ |
| Toxicity, $/event |  |  |  |  |
| Standard-of-care oral infant prophylaxis, minor (outpatient visit) | 0 | 0 | 0 | ^106,158,164^ |
| Infant bNAb prophylaxis, minor/major (outpatient visit) | 2·72 | 22·03 | 1·93 | ^106,158,164^ |
| Adult OI oral prophylaxis (co-trimoxazole), minor (outpatient visit) | 2·72 | 22·03 | 1·93 | ^106,158,164^ |
| Adult OI oral prophylaxis (co-trimoxazole), major (hospitalization) | 83·60 | 1611·45 | 52·61 | ^106,158,164^ |
| Death-related costs, $/death | 90·49 | 586·16 | 0 | ^106,111,158,164^ |
| Cost of identifying high-risk HIV-exposed infant | 31.66 | 31.66 | 31.66 | ^26,27,149,151^ |

*ART, antiretroviral therapy; mo, month; SD, standard deviation; WHO, World Health Organization; OI, opportunistic infection; NVP, nevirapine; ZDV, zidovudine; bNAb, broadly neutralizing antibody; LPV/r, lopinavir/ritonavir; EFV, efavirenz; DTG, dolutegravir; PI, protease inhibitor.*

^†^ All women included in this analysis are known to have HIV infection by delivery. Therefore, we did not model acute HIV infection during breastfeeding.

^φ^ Postnatal transmission risks among infants whose mothers are on ART while breastfeeding account for the potential impact of antiretrovirals transferred through breastmilk.

^‡^ Infants with known, high-risk HIV exposure received dual oral infant prophylaxis with nevirapine (NVP) + zidovudine (ZDV) for 12 weeks. Infants with known, low-risk HIV exposure received NVP alone for 6 weeks.

^§^ The probability of receiving broadly neutralizing antibody (bNAb) prophylaxis was based on the probability of receiving World Health Organization (WHO) Expanded Program on Immunization vaccines, by age at recommended immunization.

^¶^ A reduction in the risk of intrapartum transmission with use of oral infant prophylaxis was only applied to infants born to mothers who were known to have HIV infection, but who were not on antiretroviral therapy (ART) at delivery. The impact of oral infant prophylaxis on intrapartum transmission among mothers on ART at delivery is already captured in the on ART perinatal transmission estimates.

# **Supplementary Table 3.** Justification of sensitivity analysis ranges

| **Model input parameter** | **Base case value** | **Sensitivity analysis range** | **Justification** |
| --- | --- | --- | --- |
| Antiretroviral therapy (ART) costs (per mo.) | Pediatric ART:  *1^st^ Line:* $5·65 - $11·70  *2^nd^ Line:* $12·43 - $25·99  Adult ART:  *1^st^ Line:* $5·50  *2^nd^ Line:* $23·00 | 0·5x – 2·0x | By halving and doubling all base case ART costs, we aim to capture a plausible range of potential cost changes due to reasons such as new formulations coming to market or price adjustments of existing formulations. |
| Broadly neutralizing antibody (bNAb) cost (per dose) | $20 | $5 - $100 | This range was chosen to capture the lower and upper bounds of projected bNAb costs: $6·26 and $27·32, respectively (see appendix pp 5-6). Given that our bNAb efficacy estimate assumes a three-bNAb product, we also wanted to capture the price if production cost for each bNAb was separate and equal to the upper bound (i.e., 3 x $20 for production alone). |
| BNAb effect duration | 3 mo. | 1 mo. - 6 mo. | A single, subcutaneous 20 mg/kg dose of VRC01—an older generation, single bNAb product—achieved the mean serum concentration target in infants of >50 ug/mL for 28 days following administration.^18^ VRC01’s pharmacokinetic data informed the lower bound of the sensitivity analysis range, while the upper bound was 2x the suggested efficacy duration of newer generation antibodies, such as VRC07-523LS, from similar pharmacokinetic studies.^16^ |
| BNAb efficacy | 70% | 10% - 100% | We varied bNAb efficacy widely in increments of 10% from 10% to 100% to capture all potential efficacies of a bNAb product used for infant HIV prophylaxis. |
| BNAb uptake (varying by age) | Côte d’Ivoire:  56% - 83%  South Africa:  85% - 96%  Zimbabwe:  71% - 92% | Côte d’Ivoire:  56% at all time points – 83% at all time points  South Africa:  85% at all time points – 92% at all time points  Zimbabwe:  71% at all time points – 92% at all time points | The lower bound of bNAb uptake was informed by a linear regression model of average uptake of all country-specific recommended vaccinations through 18 months.^100^ The upper bound was informed by the average uptake of recommended vaccines at birth for South Africa or the proportion of infants delivered in a health facility for Côte d’Ivoire and Zimbabwe reported by UNICEF.^42,44^ |
| Mean breastfeeding duration | Côte d’Ivoire:  *Women with HIV:* 14 mo.  *Women w/o HIV:* 19 mo.  South Africa:  *Women with HIV:* 6 mo.  *Women w/o HIV:* 12 mo.  Zimbabwe:  *Women with HIV:* 13 mo.  *Women w/o HIV:* 18 mo. | Côte d’Ivoire:  *Women with HIV:* 8 mo. - 24 mo.  *Women w/o HIV:* 8 mo. - 24 mo.  South Africa:  *Women with HIV:* 2 mo. - 18 mo.  *Women w/o HIV:* 2 mo. - 18 mo.  Zimbabwe:  *Women with HIV:* 8 mo. - 20 mo.  *Women w/o HIV:* 15 mo. - 20 mo. | In Côte d’Ivoire, we varied the range of mean breastfeeding duration to capture all plausible scenarios in a clinical setting. The same range was used for all women, irrespective of HIV status. We chose a lower bound of 8 months based on clinical assumptions. The upper bound was partially informed by subnational Multiple Indicator Cluster Survey (MICS) estimates: Nord-Est region has the longest duration of breastfeeding (21·5 months).^42^  In South Africa, the range for women without HIV was informed by published literature and survey estimates. In the Mother-Infant Health Study, women without HIV predominantly breastfed for 2 months, informing the lower bound.^166^ Demographic Health Survey (DHS) data reported that the longest median duration of breastfeeding among any subgroup was 16·5 months among women in the lowest wealth quintile, which was used to inform the upper bound.^43^  In Zimbabwe, the range for women without HIV was informed by provincial MICS data: Harare had the shortest breastfeeding duration (15·9 months) and Mashonaland Central had the longest breastfeeding duration (19·4 months).^44^ The range for women with HIV was informed by the IQR of a nationally representative sample of mother and HIV-exposed infant pairs between 2013 and 2014.^79^ |
| Cost of ascertaining high-risk status | $31·66 | $0 - $31·66 | The cost of ascertaining whether an infant has low- or high-risk HIV exposure was assumed to include the cost of a maternal viral load test. The cost components included the cost of a viral load test ($24·05), result return with counseling ($3·48), and personnel/overhead ($4·13), reported in 2020 USD.^26,27,149,151^ In sensitivity analyses, we wanted to assess whether assuming zero additional cost would make the *HR-HIVE* strategies cost-effective relative to other bNAb strategies. We chose not to increase the cost of ascertaining risk status because, compared to the base case results, it would only make the *HR-HIVE* strategies even more costly and less clinically beneficial than other strategies (i.e., dominated). |
| Maternal ART coverage during pregnancy | Côte d’Ivoire: 95%  South Africa: 97%  Zimbabwe: 89% | Côte d’Ivoire: 70% - 100%  South Africa: 60% - 100%  Zimbabwe: 65% - 100% | We chose the ranges for maternal ART coverage during pregnancy based on the 95% confidence intervals from UNAIDS 2021 data (Côte d’Ivoire: 70 - >98%; South Africa: 60 - >98%; Zimbabwe: 68 - >98%).^54^ |
| Maternal HIV prevalence and incidence | Côte d’Ivoire:  *Prevalence at delivery:* 2·6%  *Incidence postpartum:* 0·003%/mo.  South Africa:  *Prevalence at delivery:* 33·0%  *Incidence postpartum:* 0·240%/mo.  Zimbabwe:  *Prevalence at delivery:* 12·3%  *Incidence postpartum:* 0·056%/mo. | Côte d’Ivoire:  *Prevalence at delivery:* 1·0% - 5·0%  *Incidence postpartum:*  0·001%/mo. – 0·005%/mo.  South Africa:  *Prevalence at delivery:* 15·0% - 45·0%  *Incidence postpartum:*  0·107%/mo. – 0·331%/mo.  Zimbabwe:  *Prevalence at delivery:* 5·0% - 25·0%  *Incidence postpartum:*  0·022%/mo. – 0·116%/mo. | We chose the ranges for maternal HIV prevalence and incidence based on subnational estimates among women ages 15-49 years old from Population-Based HIV Impact Assessments (PHIA) data for Côte d’Ivoire and Zimbabwe and National Antenatal Sentinel HIV Survey data for South Africa. In our analysis, maternal HIV prevalence at delivery is inclusive of prevalence and incidence during pregnancy. We applied a multiplier to the base case estimate of HIV prevalence at delivery such that it captured the range of subnational estimates. We assumed the same multiplier applies to incidence postpartum.  In Côte d’Ivoire, Woroba had the lowest prevalence (1·5%) and Montagnes had the highest prevalence (5·0%).^55^ In South Africa, Western Cape province had the lowest prevalence (15·9%) KwaZulu-Natal province had the highest prevalence (41·1%).^49^ In Zimbabwe, the lower bound of maternal HIV prevalence was informed by the lower 95% confidence interval estimate of maternal HIV prevalence (9·9%). The HIV prevalence for women in Matabeleland South (24·2%) informs the upper bound.^53^ |
| Maternal knowledge of acute HIV infection | Côte d’Ivoire:  *During pregnancy:* 56%  *Postpartum:* 2%/mo.  South Africa:  *During pregnancy:* 55%  *Postpartum:* 9%/mo.  Zimbabwe:  *During pregnancy:* 70%  *Postpartum:* 5%/mo. | All countries:  *During pregnancy:* 25% - 95%  *Postpartum:* 0%/mo. – 15%/mo. | The lower bound of maternal knowledge of acute HIV infection during pregnancy was informed by published literature. In a study from Kenya, 26·7% of women who initially tested HIV-negative in antenatal care and returned to antenatal care to retest prior to delivery.^39^ We aimed to capture all plausible scenarios of maternal knowledge of acute HIV infection by setting the upper bound of obtaining knowledge during pregnancy at 95% and during the postpartum period at 15%/mo. |
| Maternal knowledge of chronic HIV infection | Côte d’Ivoire:  93%  South Africa:  99%  Zimbabwe:  98% | Côte d’Ivoire:  56-100%  South Africa:  75-100%  Zimbabwe:  80-100% | The ranges for maternal knowledge of chronic HIV infection were informed by the upper and lower bounds of 95% confidence intervals for knowledge of HIV status as reported in South Africa DHS 2016 and UNAIDS 2020 data.^43,60^ |
| Oral infant prophylaxis efficacy  Oral infant prophylaxis efficacy (continued) | Against intrapartum transmission: 69%  Against postnatal transmission: 71% | Against intrapartum transmission: 40% - 90%  Against postnatal transmission: 58% - 80% | We chose the range of oral infant prophylaxis efficacy against intrapartum transmission based on ranges reported in the PEPI, HPTN 040, and PHT-5 studies.^14,167,168^  The range for oral infant prophylaxis efficacy against intrapartum transmission was informed by the 95% confidence interval of relative risk reduction for infants given a short course of nevirapine (NVP) + zidovudine (ZDV) compared to a single dose of NVP.^14^ The range for oral infant prophylaxis efficacy against postnatal transmission was informed by the 95% confidence interval of infant HIV infection for infants given 6 weeks of NVP compared to no oral infant prophylaxis.^15^ |
| Postnatal vertical transmission risk | On ART  *Acute HIV*: 4·0%  *Chronic HIV, suppressed*: 0·06%  *Chronic HIV, not suppressed*:  0·39% – 0·78%  Off ART  *Acute HIV*: 4·6%  *Chronic HIV*: 0·89% | 0·5x – 2·0x | By halving and doubling all base case postnatal vertical transmission risks, we aim to capture a plausible range of potential transmission risks due to reasons such as adherence to ART regimens or varying HIV viral loads. |
| Postpartum maternal ART retention | Côte d’Ivoire:  *Known, high-risk / low-risk:*  *0 mo.*: 86% / 100%  *6 mo.:* 87%  *12 mo.:* 87%  *24 mo.:* 79%  *Unrecognized exposed and unexposed:*  *0 mo.*: 0%  *6 mo.:* 9%  *12 mo.:* 17%  *24 mo.:* 27%  South Africa:  *Known, high-risk / low-risk:*  *0 mo.*: 87% / 100%  *6 mo.:* 88%  *12 mo.:* 87%  *24 mo.:* 79%  *Unrecognized exposed and unexposed:*  *0 mo.*: 0%  *6 mo.:* 37%  *12 mo.:* 57%  *24 mo.:* 69%  Zimbabwe:  *Known, high-risk / low-risk:*  *0 mo.*: 59% / 100%  *6 mo.:* 88%  *12 mo.:* 87%  *24 mo.:* 79%  *Unrecognized exposed and unexposed:*  *0 mo.*: 0%  *6 mo.:* 19%  *12 mo.:* 33%  *24 mo.:* 47% | All countries:  *Known, high-risk and low-risk*  *6+ mo.*: 50% - 100%  Côte d’Ivoire:  *Unrecognized exposed and unexposed:*  *0 mo.:* 0%  *6 mo.:* 5% - 10%  *12 mo.:* 10% - 19%  *24 mo.:* 17% - 35%  South Africa:  *Unrecognized exposed and unexposed:*  *0 mo.:* 0%  *6 mo.:* 21% - 42%  *12 mo.:* 33% - 66%  *24 mo.:* 44% - 87%  Zimbabwe:  *Unrecognized exposed and unexposed:*  *0 mo.:* 0%  *6 mo.:* 11% - 21%  *12 mo.:* 19% - 38%  *24 mo.:* 30% - 59% | We varied postpartum maternal ART retention for the known, high-risk and low-risk cohorts from 50% to 100% after 6 months to capture all plausible scenarios in a clinical setting. For the unrecognized as HIV exposed and unexposed cohorts, we calculated the ranges based on receiving ART during pregnancy if HIV status is known and maternal knowledge of HIV during breastfeeding.^43,53–55^ |
| Postpartum maternal HIV incidence | Côte d’Ivoire:  0·003%/mo.  South Africa:  0·240%/mo.  Zimbabwe:  0·056%/mo. | 0·5x – 2·0x | By halving and doubling all base case postpartum maternal HIV incidence, we aim to capture a plausible range of postpartum incidence changes due to reasons such as subnational incidence rates and missing cases in survey data. |

*mo.: month; w/o: without; IQR: interquartile range.*

# **Supplementary Table 4.** Validation of CEPAC-P infant HIV vertical transmission projections

| **Country** | **CEPAC-P projected vertical transmission rate**  **in the standard of care (SOC) strategy, %** | **UNAIDS 2021 estimates,**  **% (95% CI)**^62^ |
| --- | --- | --- |
| Côte d’Ivoire | 8·4 | 8 (4-12) |
| South Africa | 4·4 | 4 (3-8) |
| Zimbabwe | 9·5 | 9 (6-12) |

*SOC: standard-of-care; CI: confidence interval.*

Model-projected estimates of vertical transmission are often higher than those observed in clinical studies since they capture infants who may have acquired HIV infection, but be undiagnosed due to loss to follow up from EID programs or death prior to diagnosis.

# **Supplementary Table 5.** Clinical and economic outcomes of all modeled bNAb infant prophylaxis programs, by country

| **Country/strategy** | **Clinical outcomes** | | | **Lifetime efficacy and costs** | | | |
| --- | --- | --- | --- | --- | --- | --- | --- |
|  | **IU/IP cumulative HIV incidence (%)** | **Postnatal cumulative HIV incidence (%)** | **Total cumulative HIV incidence (%)** | **Undiscounted life expectancy (yrs)** | **Discounted life expectancy (yrs)** | **Discounted costs ($)** | **ICER ($/YLS)** |
| **Côte d’Ivoire [CET: ICER ≤ $465/YLS (20% GDP per capita), ICER ≤ $1163/YLS (50% GDP per capita)]** | | | | | | | |
| Standard-of-care | 3·6 | 5·0 | 8·4 | 60·129 | 26·373 | 15 | Reference |
| HR-HIVE – 1 dose | 3·3 | 4·8 | 8·0 | 60·133 | 26·375 | 15 | dominated |
| HR-HIVE – 2 doses | 3·3 | 4·7 | 7·8 | 60·134 | 26·375 | 15 | dominated |
| HIVE – 1 dose | 3·3 | 4·7 | 7·8 | 60·134 | 26·375 | 14 | dominated |
| HIVE – 1 dose plus HR-HIVE – 2 doses | 3·3 | 4·6 | 7·6 | 60·136 | 26·376 | 15 | dominated |
| HR-HIVE – Extended | 3·3 | 4·3 | 7·5 | 60·136 | 26·376 | 14 | dominated |
| HIVE – 2 doses | 3·3 | 4·3 | 7·4 | 60·138 | 26·377 | 14 | dominated |
| HIVE – 1 dose plus HR-HIVE Extended | 3·3 | 4·2 | 7·3 | 60·138 | 26·377 | 14 | dominated |
| ALL – 1 dose | 3·0 | 4·6 | 7·5 | 60·139 | 26·377 | 27 | dominated |
| ALL – 1 dose plus HR-HIVE – 2 doses | 3·0 | 4·5 | 7·3 | 60·140 | 26·378 | 28 | dominated |
| ALL – 2 doses | 3·0 | 4·2 | 7·0 | 60·140 | 26·378 | 43 | dominated |
| ALL – 1 dose plus HIVE – 2 doses | 3·0 | 4·3 | 7·1 | 60·143 | 26·379 | 27 | dominated |
| ALL – 1 dose plus HR-HIVE - Extended | 3·0 | 4·1 | 7·0 | 60·143 | 26·379 | 28 | dominated |
| HIVE – Extended | 3·3 | 3·2 | 6·3 | 60·146 | 26·379 | 13 | cost-saving*^,^† |
| ALL – 1 dose plus HIVE - Extended | 3·0 | 3·1 | 6·0 | 60·150 | 26·382 | 26 | 6242 |
| ALL - Extended | 3·0 | 2·7 | 5·5 | 60·156 | 26·383 | 91 | 57 860 |
| **South Africa [CET: ICER ≤ $1131/YLS (20% GDP per capita), ICER ≤ $2828/YLS (50% GDP per capita)]** | | | | | | | |
| Standard-of-care | 2·3 | 2·2 | 4·4 | 68·924 | 28·499 | 112 | Reference |
| HR-HIVE – 1 dose | 2·1 | 2·1 | 4·0 | 68·979 | 28·518 | 115 | dominated |
| HR-HIVE – 2 doses | 2·1 | 2·1 | 4·0 | 68·986 | 28·521 | 115 | dominated |
| HR-HIVE – Extended | 2·1 | 2·0 | 3·9 | 68·995 | 28·523 | 114 | dominated |
| HIVE – 1 dose | 2·0 | 2·0 | 3·8 | 69·011 | 28·529 | 105 | dominated |
| HIVE – 1 dose plus HR-HIVE – 2 doses | 2·0 | 1·9 | 3·8 | 69·018 | 28·532 | 115 | dominated |
| HIVE – 1 dose plus HR-HIVE - Extended | 2·0 | 1·9 | 3·7 | 69·027 | 28·534 | 114 | dominated |
| HIVE – 2 doses | 2·0 | 1·8 | 3·6 | 69·045 | 28·541 | 104 | dominated |
| ALL – 1 dose | 1·8 | 1·8 | 3·5 | 69·063 | 28·548 | 116 | dominated |
| ALL – 1 dose plus HR-HIVE – 2 doses | 1·8 | 1·7 | 3·4 | 69·070 | 28·550 | 126 | dominated |
| ALL – 1 dose plus HR-HIVE - Extended | 1·8 | 1·7 | 3·4 | 69·079 | 28·553 | 125 | dominated |
| HIVE – Extended | 2·0 | 1·5 | 3·3 | 69·095 | 28·557 | 100 | cost-saving |
| ALL – 1 dose plus HIVE – 2 doses | 1·8 | 1·6 | 3·3 | 69·097 | 28·559 | 115 | dominated |
| ALL – 2 doses | 1·8 | 1·5 | 3·2 | 69·122 | 28·568 | 122 | dominated |
| ALL – 1 dose plus HIVE - Extended | 1·8 | 1·3 | 3·0 | 69·147 | 28·575 | 111 | 606 |
| ALL - Extended | 1·8 | 0·8 | 2·5 | 69·228 | 28·602 | 135 | 882*^,^† |

**Supplemental Table 5.** Clinical and economic outcomes of all modeled bNAb infant prophylaxis programs, by country (cont.)

| **Country/strategy** | **Clinical outcomes** | | | **Lifetime efficacy and costs** | | | |
| --- | --- | --- | --- | --- | --- | --- | --- |
|  | **IU/IP cumulative HIV incidence (%)** | **Postnatal cumulative HIV incidence (%)** | **Total cumulative HIV incidence (%)** | **Undiscounted life expectancy (yrs)** | **Discounted life expectancy (yrs)** | **Discounted costs ($)** | **ICER ($/YLS)** |
| **Zimbabwe [CET: ICER ≤ $243/YLS (20% GDP per capita), ICER ≤ $607/YLS (50% GDP per capita)]** | | | | | | | |
| Standard-of-care | 4·3 | 5·6 | 9·5 | 68·321 | 28·034 | 72 | Reference |
| HR-HIVE – 1 dose | 3·7 | 5·3 | 8·6 | 68·366 | 28·050 | 71 | dominated |
| HR-HIVE – 2 doses | 3·7 | 5·2 | 8·5 | 68·373 | 28·053 | 70 | dominated |
| HIVE – 1 dose | 3·7 | 5·2 | 8·4 | 68·380 | 28·055 | 67 | dominated |
| HR-HIVE – Extended | 3·7 | 4·9 | 8·2 | 68·387 | 28·057 | 69 | dominated |
| HIVE – 1 dose plus HR-HIVE – 2 doses | 3·7 | 5·0 | 8·3 | 68·387 | 28·057 | 70 | dominated |
| HIVE – 1 dose plus HR-HIVE - Extended | 3·7 | 4·7 | 8·0 | 68·401 | 28·062 | 69 | dominated |
| ALL – 1 dose | 3·4 | 4·9 | 7·9 | 68·400 | 28·062 | 79 | dominated |
| HIVE – 2 doses | 3·7 | 4·8 | 8·0 | 68·402 | 28·063 | 66 | dominated |
| ALL – 1 dose plus HR-HIVE – 2 doses | 3·4 | 4·7 | 7·8 | 68·408 | 28·065 | 83 | dominated |
| ALL – 1 dose plus HR-HIVE - Extended | 3·4 | 4·5 | 7·5 | 68·422 | 28·069 | 82 | dominated |
| ALL – 1 dose plus HIVE – 2 doses | 3·4 | 4·5 | 7·5 | 68·423 | 28·070 | 79 | dominated |
| ALL – 2 doses | 3·4 | 4·3 | 7·4 | 68·438 | 28·074 | 93 | dominated |
| HIVE – Extended | 3·7 | 3·6 | 6·9 | 68·458 | 28·081 | 61 | cost-saving*^,^† |
| ALL – 1 dose plus HIVE - Extended | 3·4 | 3·4 | 6·5 | 68·478 | 28·088 | 74 | 1731 |
| ALL - Extended | 3·4 | 2·6 | 5·7 | 68·517 | 28·101 | 134 | 4516 |

*bNAb: broadly neutralizing antibody; IU/IP: intrauterine/intrapartum; yr: year; ICER: incremental cost-effectiveness ratio; YLS: years of life saved; CET: cost-effectiveness threshold; HR-HIVE: high-risk HIV-exposed infants; HIVE: all HIV-exposed infants; ALL: all live infants at birth.*

Pediatric HIV incidence is rounded to the nearest tenth of a percent. IU/IP HIV incidence is calculated based on the number of infants exposed to HIV at birth. Postnatal and total HIV incidence is calculated based on the number of infants ever exposed to HIV through 36 months of life. Undiscounted and discounted life expectancies are rounded to the nearest ten thousandth. Costs are rounded to the nearest dollar and are presented in 2020 USD. Discounted values are discounted at 3% per year. ICERs are rounded to the nearest dollar and are calculated using unrounded discounted life expectancy and discounted costs. The cost-effective bNAb strategy was the strategy that offered the greatest increase in overall population life expectancy while still having an ICER less than the cost-effectiveness threshold when compared to the next best performing, non-dominated strategy. *Indicates the cost-effective strategy using a cost-effectiveness threshold of 20% GDP per capita. †Indicates the cost-effective strategy using a cost-effectiveness threshold of 50% GDP per capita.

# **Supplementary Table 6.** Scenario analysis: bNAbs do not reduce intrapartum transmission (base case: 70% reduction)

| **Country/strategy** | **Clinical outcomes** | | | **Lifetime efficacy and costs** | | | |
| --- | --- | --- | --- | --- | --- | --- | --- |
|  | **IU/IP cumulative HIV incidence (%)** | **Postnatal cumulative HIV incidence (%)** | **Total cumulative HIV incidence (%)** | **Undiscounted life expectancy (yrs)** | **Discounted life expectancy (yrs)** | **Discounted costs ($)** | **ICER ($/YLS)** |
| **Côte d’Ivoire [CET: ICER ≤ $465/YLS (20% GDP per capita), ICER ≤ $1163/YLS (50% GDP per capita)]** | | | | | | | |
| Standard-of-care | 3·6 | 5·0 | 8·4 | 60·129 | 26·373 | 15 | Reference |
| HR-HIVE – 1 dose | 3·6 | 4·9 | 8·3 | 60·130 | 26·374 | 15 | dominated |
| HR-HIVE – 2 doses | 3·6 | 4·7 | 8·1 | 60·131 | 26·374 | 15. | dominated |
| HIVE – 1 dose | 3·6 | 4·7 | 8·1 | 60·131 | 26·374 | 15 | dominated |
| HR-HIVE – Extended | 3·6 | 4·4 | 7·8 | 60·134 | 26·375 | 15 | dominated |
| HIVE – 2 doses | 3·6 | 4·3 | 7·8 | 60·135 | 26·375 | 14 | dominated |
| ALL – 1 dose | 3·6 | 4·7 | 8·1 | 60·134 | 26·375 | 28 | dominated |
| ALL – 2 doses | 3·6 | 4·2 | 7·6 | 60·135 | 26·376 | 43 | dominated |
| HIVE – Extended | 3·6 | 3·2 | 6·6 | 60·142 | 26·378 | 13 | cost-saving*^,^† |
| ALL – 1 dose plus HIVE - Extended | 3·6 | 3·1 | 6·6 | 60·145 | 26·380 | 27 | 10 008 |
| ALL - Extended | 3·6 | 2·7 | 6·1 | 60·151 | 26·381 | 92 | 59 542 |
| **South Africa [CET: ICER ≤ $1131/YLS (20% GDP per capita), ICER ≤ $2828/YLS (50% GDP per capita)]** | | | | | | | |
| Standard-of-care | 2·3 | 2·2 | 4·4 | 68·924 | 28·499 | 112 | Reference |
| HR-HIVE – 1 dose | 2·3 | 2·1 | 4·3 | 68·938 | 28·504 | 121 | dominated |
| HR-HIVE – 2 doses | 2·3 | 2·1 | 4·2 | 68·945 | 28·506 | 121 | dominated |
| HR-HIVE – Extended | 2·3 | 2·0 | 4·2 | 68·953 | 28·509 | 120 | dominated |
| HIVE – 1 dose | 2·3 | 2·0 | 4·2 | 68·955 | 28·510 | 113 | dominated |
| ALL – 1 dose | 2·3 | 1·8 | 4·0 | 68·986 | 28·521 | 127 | dominated |
| HIVE – 2 doses | 2·3 | 1·8 | 4·0 | 68·989 | 28·522 | 112 | dominated |
| HIVE – Extended | 2·3 | 1·5 | 3·7 | 69·038 | 28·538 | 109 | cost-saving |
| ALL – 2 doses | 2·3 | 1·5 | 3·7 | 69·044 | 28·541 | 133 | dominated |
| ALL – 1 dose plus HIVE - Extended | 2·3 | 1·3 | 3·5 | 69·069 | 28·549 | 122 | dominated |
| ALL - Extended | 2·3 | 0·8 | 3·0 | 69·150 | 28·575 | 146 | 998*^,^† |
| **Zimbabwe [CET: ICER ≤ $243/YLS (20% GDP per capita), ICER ≤ $607/YLS (50% GDP per capita)]** | | | | | | | |
| Standard-of-care | 4·3 | 5·6 | 9·5 | 68·321 | 28·034 | 72 | Reference |
| HR-HIVE – 1 dose | 4·3 | 5·4 | 9·2 | 68·337 | 28·040 | 74 | dominated |
| HR-HIVE – 2 doses | 4·3 | 5·2 | 9·1 | 68·344 | 28·042 | 74 | dominated |
| HIVE – 1 dose | 4·3 | 5·2 | 9·0 | 68·346 | 28·043 | 71 | dominated |
| HR-HIVE – Extended | 4·3 | 4·9 | 8·8 | 68·358 | 28·046 | 73 | dominated |
| ALL – 1 dose | 4·3 | 4·9 | 8·8 | 68·357 | 28·046 | 85 | dominated |
| HIVE – 2 doses | 4·3 | 4·8 | 8·6 | 68·369 | 28·051 | 70 | dominated |
| ALL – 2 doses | 4·3 | 4·4 | 8·2 | 68·395 | 28·058 | 98 | dominated |
| HIVE – Extended | 4·3 | 3·7 | 7·6 | 68·424 | 28·068 | 65 | cost-saving*^,^† |
| ALL – 1 dose plus HIVE - Extended | 4·3 | 3·4 | 7·3 | 68·434 | 28·072 | 79 | 3855 |
| ALL - Extended | 4·3 | 2·6 | 6·5 | 68·472 | 28·085 | 139 | 4584 |

*bNAb: broadly neutralizing antibody; IU/IP: intrauterine/intrapartum; yr: year; ICER: incremental cost-effectiveness ratio; YLS: years of life saved; CET: cost-effectiveness threshold; HR-HIVE: high-risk HIV-exposed infants; HIVE: all HIV-exposed infants; ALL: all live infants at birth.*

Pediatric HIV incidence is rounded to the nearest tenth of a percent. IU/IP HIV incidence is calculated based on the number of infants exposed to HIV at birth. Postnatal and total HIV incidence is calculated based on the number of infants ever exposed to HIV through 36 months of life. Undiscounted and discounted life expectancies are rounded to the nearest ten thousandth. Costs are rounded to the nearest dollar and are presented in 2020 USD. Discounted values are discounted at 3% per year. ICERs are rounded to the nearest dollar and are calculated using unrounded discounted life expectancy and discounted costs. The cost-effective bNAb strategy was the strategy that offered the greatest increase in overall population life expectancy while still having an ICER less than the cost-effectiveness threshold when compared to the next best performing, non-dominated strategy. *Indicates the cost-effective strategy using a cost-effectiveness threshold of 20% GDP per capita. †Indicates the cost-effective strategy using a cost-effectiveness threshold of 50% GDP per capita.

| **Country/strategy** | **Clinical outcomes** | | | **Lifetime efficacy and costs** | | | |
| --- | --- | --- | --- | --- | --- | --- | --- |
|  | **IU/IP cumulative HIV incidence (%)** | **Postnatal cumulative HIV incidence (%)** | **Total cumulative HIV incidence (%)** | **Undiscounted life expectancy (yrs)** | **Discounted life expectancy (yrs)** | **Discounted costs ($)** | **ICER ($/YLS)** |
| **Côte d’Ivoire [CET: ICER ≤ $465/YLS (20% GDP per capita), ICER ≤ $1163/YLS (50% GDP per capita)]** | | | | | | | |
| Standard-of-care | 3·6 | 5·0 | 8·4 | 60·129 | 26·373 | 15 | Reference |
| HR-HIVE – 1 dose | 3·3 | 5·0 | 8·1 | 60·131 | 26·374 | 15 | dominated |
| HR-HIVE – 2 doses | 3·3 | 4·9 | 8·0 | 60·132 | 26·374 | 15 | dominated |
| HIVE – 1 dose | 3·3 | 4·9 | 8·0 | 60·132 | 26·374 | 14 | dominated |
| HR-HIVE – Extended | 3·3 | 4·5 | 7·6 | 60·135 | 26·375 | 15 | dominated |
| ALL – 1 dose | 3·0 | 4·9 | 7·7 | 60·134 | 26·375 | 28 | dominated |
| HIVE – 2 doses | 3·3 | 4·5 | 7·6 | 60·136 | 26·376 | 14 | dominated |
| ALL – 2 doses | 3·0 | 4·4 | 7·2 | 60·144 | 26·378 | 43 | dominated |
| HIVE – Extended | 3·3 | 3·4 | 6·5 | 60·143 | 26·379 | 13 | cost-saving*^,^† |
| ALL – 1 dose plus HIVE - Extended | 3·0 | 3·3 | 6·2 | 60·145 | 26·379 | 26 | 15 769 |
| ALL - Extended | 3·0 | 2·9 | 5·8 | 60·156 | 26·383 | 91 | 21 344 |
| **South Africa [CET: ICER ≤ $1131/YLS (20% GDP per capita), ICER ≤ $2828/YLS (50% GDP per capita)]** | | | | | | | |
| Standard-of-care | 2·3 | 2·2 | 4·4 | 68·924 | 28·499 | 112 | Reference |
| HR-HIVE – 1 dose | 2·1 | 2·2 | 4·1 | 68·969 | 28·514 | 116 | dominated |
| HR-HIVE – 2 doses | 2·1 | 2·1 | 4·0 | 68·975 | 28·517 | 116 | dominated |
| HR-HIVE – Extended | 2·1 | 2·1 | 4·0 | 68·984 | 28·519 | 115 | dominated |
| HIVE – 1 dose | 2·0 | 2·1 | 3·9 | 69·000 | 28·525 | 106 | dominated |
| HIVE – 2 doses | 2·0 | 1·9 | 3·7 | 69·032 | 28·536 | 105 | dominated |
| ALL – 1 dose | 1·8 | 1·9 | 3·6 | 69·058 | 28·544 | 118 | dominated |
| HIVE – Extended | 2·0 | 1·6 | 3·4 | 69·080 | 28·552 | 102 | cost-saving |
| ALL – 2 doses | 1·8 | 1·5 | 3·2 | 69·107 | 28·562 | 123 | dominated |
| ALL – 1 dose plus HIVE - Extended | 1·8 | 1·4 | 3·1 | 69·138 | 28·571 | 113 | 587 |
| ALL - Extended | 1·8 | 0·9 | 2·6 | 69·211 | 28·596 | 136 | 941*^,^† |
| **Zimbabwe [CET: ICER ≤ $243/YLS (20% GDP per capita), ICER ≤ $607/YLS (50% GDP per capita)]** | | | | | | | |
| Standard-of-care | 4·3 | 5·6 | 9·5 | 68·321 | 28·034 | 72 | Reference |
| HR-HIVE – 1 dose | 3·7 | 5·6 | 8·9 | 68·349 | 28·044 | 72 | dominated |
| HR-HIVE – 2 doses | 3·7 | 5·5 | 8·8 | 68·356 | 28·046 | 72 | dominated |
| HIVE – 1 dose | 3·7 | 5·5 | 8·7 | 68·361 | 28·048 | 69 | dominated |
| HR-HIVE – Extended | 3·7 | 5·2 | 8·5 | 68·370 | 28·051 | 71 | dominated |
| ALL – 1 dose | 3·4 | 5·2 | 8·3 | 68·381 | 28·056 | 81 | dominated |
| HIVE – 2 doses | 3·7 | 5·1 | 8·3 | 68·383 | 28·056 | 68 | dominated |
| ALL – 2 doses | 3·4 | 4·7 | 7·7 | 68·411 | 28·066 | 95 | dominated |
| HIVE – Extended | 3·7 | 4·0 | 7·2 | 68·438 | 28·074 | 63 | cost-saving*^,^† |
| ALL – 1 dose plus HIVE - Extended | 3·4 | 3·7 | 6·8 | 68·457 | 28·081 | 76 | 1696 |
| ALL - Extended | 3·4 | 2·9 | 6·0 | 68·497 | 28·093 | 136 | 5020 |

# **Supplementary Table 7.** Scenario analysis: bNAbs replace WHO-recommended standard-of-care oral infant prophylaxis

*bNAb: broadly neutralizing antibody; IU/IP: intrauterine/intrapartum; yr: year; ICER: incremental cost-effectiveness ratio; YLS: years of life saved; CET: cost-effectiveness threshold; HR-HIVE: high-risk HIV-exposed infants; HIVE: all HIV-exposed infants; ALL: all live infants at birth.*

In this scenario, infants receive either bNAbs or WHO-recommended standard-of-care oral prophylaxis, but not both; sub-populations not eligible to receive bNAbs in a strategy continue to be eligible for WHO-recommended standard-of-care oral prophylaxis. For example, in the HR-HIVE strategy, infants who are known, high-risk, HIV-exposed could only receive bNAbs but not oral prophylaxis while all other sub-populations would still be eligible to receive WHO-recommended standard-of-care oral prophylaxis. Pediatric HIV incidence is rounded to the nearest tenth of a percent. IU/IP HIV incidence is calculated based on the number of infants exposed to HIV at birth. Postnatal and total HIV incidence is calculated based on the number of infants ever exposed to HIV through 36 months of life. Undiscounted and discounted life expectancies are rounded to the nearest ten thousandth. Costs are rounded to the nearest dollar and are presented in 2020 USD. Discounted values are discounted at 3% per year. ICERs are rounded to the nearest dollar and are calculated using unrounded discounted life expectancy and discounted costs. The cost-effective bNAb strategy was the strategy that offered the greatest increase in overall population life expectancy while still having an ICER less than the cost-effectiveness threshold when compared to the next best performing, non-dominated strategy. *Indicates the cost-effective strategy using a cost-effectiveness threshold of 20% GDP per capita. †Indicates the cost-effective strategy using a cost-effectiveness threshold of 50% GDP per capita.

# **Supplementary Table 8.** Scenario analysis: maternal HIV prevalence and incidence

| **Country/strategy** | **Clinical outcomes** | | | **Lifetime efficacy and costs** | | | |
| --- | --- | --- | --- | --- | --- | --- | --- |
|  | **IU/IP cumulative HIV incidence (%)** | **Postnatal cumulative HIV incidence (%)** | **Total cumulative HIV incidence (%)** | **Undiscounted life expectancy (yrs)** | **Discounted life expectancy (yrs)** | **Discounted costs ($)** | **ICER ($/YLS)** |
| **Côte d’Ivoire [CET: ICER ≤ $465/YLS (20% GDP per capita), ICER ≤ $1163/YLS (50% GDP per capita)]** | | | | | | | |
| ***Low maternal HIV prevalence/incidence*** | |  |  |  |  |  |  |
| Standard-of-care | 3·6 | 5·0 | 8·4 | 60·177 | 26·392 | 5 | Reference |
| HR-HIVE – 1 dose | 3·3 | 4·9 | 8·0 | 60·178 | 26·393 | 5 | dominated |
| HR-HIVE – 2 doses | 3·3 | 4·7 | 7·8 | 60·179 | 26·393 | 5 | dominated |
| HIVE – 1 dose | 3·3 | 4·7 | 7·8 | 60·179 | 26·393 | 5 | dominated |
| HR-HIVE – Extended | 3·3 | 4·4 | 7·5 | 60·180 | 26·393 | 5 | dominated |
| HIVE – 2 doses | 3·3 | 4·3 | 7·4 | 60·180 | 26·393 | 5 | dominated |
| ALL – 2 doses | 3·0 | 4·2 | 7·0 | 60·181 | 26·394 | 35 | dominated |
| ALL – 1 dose | 3·0 | 4·7 | 7·5 | 60·183 | 26·394 | 20 | dominated |
| HIVE – Extended | 3·3 | 3·2 | 6·3 | 60·183 | 26·395 | 4 | cost-saving*^,^† |
| ALL – 1 dose plus HIVE - Extended | 3·0 | 3·1 | 6·0 | 60·187 | 26·396 | 19 | 9521 |
| ALL - Extended | 3·0 | 2·7 | 5·6 | 60·190 | 26·396 | 85 | 213 006 |
| ***High maternal HIV prevalence/incidence*** | |  |  |  |  |  |  |
| Standard-of-care | 3·6 | 5·0 | 8·4 | 60·059 | 26·345 | 27 | Reference |
| HR-HIVE – 1 dose | 3·3 | 4·8 | 7·9 | 60·065 | 26·348 | 27 | dominated |
| HR-HIVE – 2 doses | 3·3 | 4·7 | 7·8 | 60·067 | 26·349 | 27 | dominated |
| HIVE – 1 dose | 3·3 | 4·7 | 7·8 | 60·069 | 26·349 | 26 | dominated |
| HR-HIVE – Extended | 3·3 | 4·3 | 7·5 | 60·072 | 26·351 | 27 | dominated |
| HIVE – 2 doses | 3·3 | 4·3 | 7·4 | 60·075 | 26·352 | 25 | dominated |
| ALL – 1 dose | 3·0 | 4·6 | 7·5 | 60·077 | 26·353 | 40 | dominated |
| ALL – 2 doses | 3·0 | 4·1 | 7·0 | 60·081 | 26·355 | 54 | dominated |
| HIVE – Extended | 3·3 | 3·1 | 6·3 | 60·090 | 26·357 | 23 | cost-saving*^,^† |
| ALL – 1 dose plus HIVE - Extended | 3·0 | 3·1 | 6·0 | 60·098 | 26·361 | 37 | 4158 |
| ALL - Extended | 3·0 | 2·7 | 5·5 | 60·106 | 26·363 | 99 | 32 843 |
| **South Africa [CET: ICER ≤ $1131/YLS (20% GDP per capita), ICER ≤ $2828/YLS (50% GDP per capita)]** | | | | | | | |
| ***Low maternal HIV prevalence/incidence*** | |  |  |  |  |  |  |
| Standard-of-care | 2·5 | 2·5 | 4·8 | 69·322 | 28·639 | 56 | Reference |
| HR-HIVE – 1 dose | 2·2 | 2·3 | 4·4 | 69·349 | 28·648 | 57 | dominated |
| HR-HIVE – 2 doses | 2·2 | 2·3 | 4·4 | 69·352 | 28·649 | 57 | dominated |
| HR-HIVE – Extended | 2·2 | 2·2 | 4·3 | 69·356 | 28·650 | 56 | dominated |
| HIVE – 1 dose | 2·1 | 2·2 | 4·2 | 69·363 | 28·653 | 52 | dominated |
| HIVE – 2 doses | 2·1 | 2·0 | 4·0 | 69·378 | 28·658 | 52 | dominated |
| ALL – 1 dose | 2·0 | 2·0 | 3·8 | 69·389 | 28·661 | 70 | dominated |
| HIVE – Extended | 2·1 | 1·7 | 3·7 | 69·401 | 28·665 | 50 | cost-saving* |
| ALL – 2 doses | 2·0 | 1·6 | 3·4 | 69·423 | 28·673 | 81 | dominated |
| ALL – 1 dose plus HIVE - Extended | 2·0 | 1·5 | 3·3 | 69·427 | 28·674 | 68 | 2065 |
| ALL - Extended | 2·0 | 0·9 | 2·7 | 69·477 | 28·690 | 104 | 2262† |

| **Country/strategy** | **Clinical outcomes** | | | **Lifetime efficacy and costs** | | | |
| --- | --- | --- | --- | --- | --- | --- | --- |
|  | **IU/IP cumulative HIV incidence (%)** | **Postnatal cumulative HIV incidence (%)** | **Total cumulative HIV incidence (%)** | **Undiscounted life expectancy (yrs)** | **Discounted life expectancy (yrs)** | **Discounted costs ($)** | **ICER ($/YLS)** |
| **South Africa [CET: ICER ≤ $1131/YLS (20% GDP per capita), ICER ≤ $2828/YLS (50% GDP per capita)]** | | | | | | | |
| ***High maternal HIV prevalence/incidence*** | |  |  |  |  |  |  |
| Standard-of-care | 2·2 | 2·0 | 4·1 | 68·714 | 28·426 | 144 | Reference |
| HR-HIVE – 1 dose | 1·9 | 1·9 | 3·7 | 68·786 | 28·450 | 149 | dominated |
| HR-HIVE – 2 doses | 1·9 | 1·9 | 3·7 | 68·795 | 28·453 | 149 | dominated |
| HR-HIVE – Extended | 1·9 | 1·8 | 3·7 | 68·807 | 28·457 | 148 | dominated |
| HIVE – 1 dose | 1·8 | 1·8 | 3·5 | 68·830 | 28·466 | 135 | dominated |
| HIVE – 2 doses | 1·8 | 1·6 | 3·3 | 68·876 | 28·482 | 134 | dominated |
| ALL – 1 dose | 1·7 | 1·6 | 3·2 | 68·888 | 28·487 | 142 | dominated |
| HIVE – Extended | 1·8 | 1·3 | 3·0 | 68·945 | 28·504 | 129 | cost-saving |
| ALL – 2 doses | 1·7 | 1·3 | 2·9 | 68·963 | 28·512 | 145 | dominated |
| ALL – 1 dose plus HIVE - Extended | 1·7 | 1·1 | 2·8 | 69·003 | 28·525 | 135 | 298 |
| ALL - Extended | 1·7 | 0·7 | 2·4 | 69·094 | 28·555 | 152 | 548*^,^† |
| **Zimbabwe [CET: ICER ≤ $243/YLS (20% GDP per capita), ICER ≤ $607/YLS (50% GDP per capita)]** | | | | | | | |
| ***Low maternal HIV prevalence/incidence*** | |  |  |  |  |  |  |
| Standard-of-care | 4·4 | 5·8 | 9·6 | 68·675 | 28·160 | 29 | Reference |
| HR-HIVE – 1 dose | 3·8 | 5·4 | 8·8 | 68·694 | 28·166 | 29 | dominated |
| HR-HIVE – 2 doses | 3·8 | 5·3 | 8·7 | 68·697 | 28·167 | 29 | dominated |
| HIVE – 1 dose | 3·7 | 5·3 | 8·6 | 68·699 | 28·168 | 27 | dominated |
| HR-HIVE – Extended | 3·8 | 5·0 | 8·4 | 68·703 | 28·169 | 28 | dominated |
| ALL – 1 dose | 3·5 | 5·0 | 8·1 | 68·708 | 28·170 | 43 | dominated |
| HIVE – 2 doses | 3·7 | 4·9 | 8·2 | 68·708 | 28·171 | 27 | dominated |
| ALL – 2 doses | 3·5 | 4·4 | 7·5 | 68·728 | 28·176 | 59 | dominated |
| HIVE – Extended | 3·7 | 3·8 | 7·1 | 68·731 | 28·179 | 25 | cost-saving*^,^† |
| ALL – 1 dose plus HIVE - Extended | 3·5 | 3·5 | 6·6 | 68·739 | 28·181 | 41 | 7410 |
| ALL - Extended | 3·5 | 2·6 | 5·8 | 68·756 | 28·187 | 109 | 10 687 |
| ***High maternal HIV prevalence/incidence*** | |  |  |  |  |  |  |
| Standard-of-care | 4·2 | 5·4 | 9·1 | 67·742 | 27·829 | 139 | Reference |
| HR-HIVE – 1 dose | 3·6 | 5·1 | 8·3 | 67·829 | 27·860 | 137 | dominated |
| HR-HIVE – 2 doses | 3·6 | 5·0 | 8·2 | 67·843 | 27·865 | 137 | dominated |
| HIVE – 1 dose | 3·5 | 4·9 | 8·1 | 67·857 | 27·870 | 130 | dominated |
| HR-HIVE – Extended | 3·6 | 4·7 | 7·9 | 67·871 | 27·874 | 134 | dominated |
| HIVE – 2 doses | 3·5 | 4·5 | 7·7 | 67·903 | 27·886 | 128 | dominated |
| ALL – 1 dose | 3·3 | 4·7 | 7·7 | 67·903 | 27·886 | 138 | dominated |
| ALL – 2 doses | 3·3 | 4·2 | 7·1 | 67·973 | 27·909 | 148 | dominated |
| HIVE – Extended | 3·5 | 3·4 | 6·6 | 68·016 | 27·922 | 119 | cost-saving* |
| ALL – 1 dose plus HIVE - Extended | 3·3 | 3·2 | 6·2 | 68·062 | 27·938 | 126 | 494† |
| ALL - Extended | 3·3 | 2·4 | 5·5 | 68·134 | 27·963 | 173 | 1894 |

**Supplementary Table 8.** Scenario analysis: maternal HIV prevalence and incidence (cont.)

*bNAb: broadly neutralizing antibody; IU/IP: intrauterine/intrapartum; yr: year; ICER: incremental cost-effectiveness ratio; YLS: years of life saved; CET: cost-effectiveness threshold; HR-HIVE: high-risk HIV-exposed infants; HIVE: all HIV-exposed infants; ALL: all live infants at birth.*

The setting-specific maternal HIV prevalence and incidence are specified in Supplementary Table 2 (base case) and Supplementary Table 3 (low/high scenarios). Pediatric HIV incidence is rounded to the nearest tenth of a percent. IU/IP HIV incidence is calculated based on the number of infants exposed to HIV at birth. Postnatal and total HIV incidence is calculated based on the number of infants ever exposed to HIV through 36 months of life. Undiscounted and discounted life expectancies are rounded to the nearest ten thousandth. Costs are rounded to the nearest dollar and are presented in 2020 USD. Discounted values are discounted at 3% per year. ICERs are rounded to the nearest dollar and are calculated using unrounded discounted life expectancy and discounted costs. The cost-effective bNAb strategy was the strategy that offered the greatest increase in overall population life expectancy while still having an ICER less than the cost-effectiveness threshold when compared to the next best performing, non-dominated strategy. * Indicates the cost-effective strategy using a cost-effectiveness threshold of 20% GDP per capita. † Indicates the cost-effective strategy using a cost-effectiveness threshold of 50% GDP per capita.

# **Supplementary Table 9.** One-way sensitivity analysis: bNAb efficacy against intrapartum and postnatal transmission (base case: 70% reduction)

| **Country/strategy** | **Clinical outcomes** | | | **Lifetime efficacy and costs** | | | |
| --- | --- | --- | --- | --- | --- | --- | --- |
|  | **IU/IP cumulative HIV incidence (%)** | **Postnatal cumulative HIV incidence (%)** | **Total cumulative HIV incidence (%)** | **Undiscounted life expectancy (yrs)** | **Discounted life expectancy (yrs)** | **Discounted costs ($)** | **ICER ($/YLS)** |
| **Côte d’Ivoire [CET: ICER ≤ $465/YLS (20% GDP per capita), ICER ≤ $1163/YLS (50% GDP per capita)]** | | | | | | | |
| ***bNAb efficacy: 10%*** |  |  |  |  |  |  |  |
| Standard-of-care | 3·6 | 5·0 | 8·4 | 60·129 | 26·373 | 15 | Reference* |
| HR-HIVE – 1 dose | 3·6 | 5·0 | 8·3 | 60·130 | 26·373 | 15 | dominated |
| HR-HIVE – 2 doses | 3·6 | 4·9 | 8·3 | 60·130 | 26·373 | 15 | dominated |
| HIVE – 1 dose | 3·6 | 4·9 | 8·3 | 60·130 | 26·374 | 15 | 706† |
| HR-HIVE – Extended | 3·6 | 4·9 | 8·2 | 60·130 | 26·374 | 16 | dominated |
| HIVE – 2 doses | 3·6 | 4·9 | 8·2 | 60·131 | 26·374 | 15 | dominated |
| ALL – 2 doses | 3·5 | 4·9 | 8·2 | 60·130 | 26·374 | 45 | dominated |
| HIVE – Extended | 3·6 | 4·7 | 8·1 | 60·132 | 26·374 | 16 | 1593 |
| ALL – 1 dose | 3·5 | 4·9 | 8·2 | 60·133 | 26·375 | 28 | dominated |
| ALL – 1 dose plus HIVE - Extended | 3·5 | 4·7 | 8·0 | 60·134 | 26·375 | 29 | 11 561 |
| ALL - Extended | 3·5 | 4·7 | 8·0 | 60·137 | 26·375 | 95 | 615 409 |
| ***bNAb efficacy: 100%*** |  |  |  |  |  |  |  |
| Standard-of-care | 3·6 | 5·0 | 8·4 | 60·129 | 26·373 | 15 | Reference |
| HR-HIVE – 1 dose | 3·2 | 4·8 | 7·8 | 60·134 | 26·375 | 15 | dominated |
| HR-HIVE – 2 doses | 3·2 | 4·6 | 7·6 | 60·136 | 26·376 | 14 | dominated |
| HIVE – 1 dose | 3·1 | 4·6 | 7·5 | 60·137 | 26·376 | 14 | dominated |
| HR-HIVE – Extended | 3·2 | 4·1 | 7·1 | 60·139 | 26·377 | 14 | dominated |
| HIVE – 2 doses | 3·1 | 4·0 | 7·0 | 60·141 | 26·378 | 13 | dominated |
| ALL – 1 dose | 2·8 | 4·5 | 7·1 | 60·143 | 26·379 | 27 | dominated |
| ALL – 2 doses | 2·8 | 3·8 | 6·4 | 60·146 | 26·380 | 42 | dominated |
| HIVE – Extended | 3·1 | 2·4 | 5·3 | 60·153 | 26·382 | 11 | cost-saving*^,^† |
| ALL – 1 dose plus HIVE - Extended | 2·8 | 2·3 | 4·9 | 60·159 | 26·385 | 24 | 5017 |
| ALL - Extended | 2·8 | 1·6 | 4·3 | 60·166 | 26·387 | 89 | 38 627 |
| **South Africa [CET: ICER ≤ $1131/YLS (20% GDP per capita), ICER ≤ $2828/YLS (50% GDP per capita)]** | | | | | | | |
| ***bNAb efficacy: 10%*** |  |  |  |  |  |  |  |
| Standard-of-care | 2·3 | 2·2 | 4·4 | 68·924 | 28·499 | 112 | Reference |
| HR-HIVE – 1 dose | 2·3 | 2·2 | 4·3 | 68·931 | 28·502 | 122 | dominated |
| HR-HIVE – 2 doses | 2·3 | 2·2 | 4·3 | 68·933 | 28·502 | 122 | dominated |
| HR-HIVE – Extended | 2·3 | 2·2 | 4·3 | 68·934 | 28·503 | 122 | dominated |
| HIVE – 1 dose | 2·3 | 2·2 | 4·3 | 68·934 | 28·503 | 115 | 1009 |
| ALL – 1 dose | 2·3 | 2·1 | 4·2 | 68·938 | 28·504 | 132 | dominated |
| HIVE – 2 doses | 2·3 | 2·1 | 4·3 | 68·940 | 28·505 | 118 | dominated |
| HIVE – Extended | 2·3 | 2·1 | 4·2 | 68·947 | 28·507 | 120 | 1118*^,^† |
| ALL – 2 doses | 2·3 | 2·1 | 4·2 | 68·950 | 28·508 | 143 | dominated |
| ALL – 1 dose plus HIVE - Extended | 2·3 | 2·1 | 4·2 | 68·951 | 28·509 | 136 | dominated |
| ALL - Extended | 2·3 | 2·0 | 4·1 | 68·967 | 28·513 | 169 | 7902 |

| **Country/strategy** | **Clinical outcomes** | | | **Lifetime efficacy and costs** | | | |
| --- | --- | --- | --- | --- | --- | --- | --- |
|  | **IU/IP cumulative HIV incidence (%)** | **Postnatal cumulative HIV incidence (%)** | **Total cumulative HIV incidence (%)** | **Undiscounted life expectancy (yrs)** | **Discounted life expectancy (yrs)** | **Discounted costs ($)** | **ICER ($/YLS)** |
| **South Africa [CET: ICER ≤ $1131/YLS (20% GDP per capita), ICER ≤ $2828/YLS (50% GDP per capita)]** | | | | | | | |
| ***bNAb efficacy: 100%*** |  |  |  |  |  |  |  |
| Standard-of-care | 2·3 | 2·2 | 4·4 | 68·924 | 28·499 | 112 | Reference |
| HR-HIVE – 1 dose | 1·9 | 2·1 | 3·9 | 69·004 | 28·527 | 112 | dominated |
| HR-HIVE – 2 doses | 1·9 | 2·0 | 3·8 | 69·013 | 28·530 | 111 | dominated |
| HR-HIVE – Extended | 1·9 | 1·9 | 3·7 | 69·026 | 28·534 | 110 | dominated |
| HIVE – 1 dose | 1·8 | 1·9 | 3·6 | 69·050 | 28·543 | 100 | dominated |
| HIVE – 2 doses | 1·8 | 1·6 | 3·3 | 69·098 | 28·559 | 97 | dominated |
| ALL – 1 dose | 1·6 | 1·6 | 3·1 | 69·127 | 28·570 | 108 | dominated |
| HIVE – Extended | 1·8 | 1·2 | 2·9 | 69·169 | 28·582 | 90 | cost-saving |
| ALL – 2 doses | 1·6 | 1·1 | 2·6 | 69·210 | 28·599 | 111 | dominated |
| ALL – 1 dose plus HIVE - Extended | 1·6 | 0·9 | 2·4 | 69·246 | 28·609 | 98 | 295 |
| ALL - Extended | 1·6 | 0·2 | 1·7 | 69·363 | 28·648 | 117 | 476*^,^† |
| **Zimbabwe [CET: ICER ≤ $243/YLS (20% GDP per capita), ICER ≤ $607/YLS (50% GDP per capita)]** | | | | | | | |
| ***bNAb efficacy: 10%*** |  |  |  |  |  |  |  |
| Standard-of-care | 4·3 | 5·6 | 9·5 | 68·321 | 28·034 | 72 | Reference* |
| ALL – 1 dose | 4·2 | 5·5 | 9·2 | 68·328 | 28·036 | 87 | dominated |
| HR-HIVE – 1 dose | 4·3 | 5·6 | 9·3 | 68·327 | 28·036 | 75 | dominated |
| HR-HIVE – 2 doses | 4·3 | 5·6 | 9·3 | 68·328 | 28·037 | 75 | dominated |
| HIVE – 1 dose | 4·2 | 5·6 | 9·3 | 68·330 | 28·037 | 72 | dominated |
| HR-HIVE – Extended | 4·3 | 5·5 | 9·3 | 68·330 | 28·037 | 76 | 276† |
| HIVE – 2 doses | 4·2 | 5·5 | 9·3 | 68·333 | 28·038 | 74 | dominated |
| ALL – 2 doses | 4·2 | 5·5 | 9·2 | 68·339 | 28·039 | 104 | dominated |
| ALL – 1 dose plus HIVE - Extended | 4·2 | 5·3 | 9·0 | 68·338 | 28·039 | 92 | 1379 |
| HIVE – Extended | 4·2 | 5·4 | 9·1 | 68·340 | 28·040 | 77 | dominated |
| ALL - Extended | 4·2 | 5·2 | 8·9 | 68·342 | 28·041 | 157 | 82 283 |
| ***bNAb efficacy: 100%*** |  |  |  |  |  |  |  |
| Standard-of-care | 4·3 | 5·6 | 9·5 | 68·321 | 28·034 | 72 | Reference |
| HR-HIVE – 1 dose | 3·5 | 5·2 | 8·3 | 68·386 | 28·057 | 68 | dominated |
| HR-HIVE – 2 doses | 3·5 | 5·0 | 8·1 | 68·396 | 28·061 | 67 | dominated |
| HIVE – 1 dose | 3·4 | 4·9 | 7·9 | 68·405 | 28·064 | 64 | dominated |
| HR-HIVE – Extended | 3·5 | 4·6 | 7·7 | 68·417 | 28·067 | 65 | dominated |
| HIVE – 2 doses | 3·4 | 4·4 | 7·4 | 68·437 | 28·075 | 62 | dominated |
| ALL – 1 dose | 3·1 | 4·5 | 7·3 | 68·437 | 28·076 | 75 | dominated |
| ALL – 2 doses | 3·1 | 3·7 | 6·5 | 68·490 | 28·093 | 87 | dominated |
| HIVE – Extended | 3·4 | 2·7 | 5·8 | 68·518 | 28·101 | 53 | cost-saving*^,^† |
| ALL – 1 dose plus HIVE - Extended | 3·1 | 2·4 | 5·1 | 68·550 | 28·113 | 65 | 995 |
| ALL - Extended | 3·1 | 1·2 | 4·0 | 68·609 | 28·132 | 121 | 2849 |

**Supplementary Table 9.** One-way sensitivity analysis: bNAb efficacy against intrapartum and postnatal transmission (base case: 70% reduction) (cont.)

*bNAb: broadly neutralizing antibody; IU/IP: intrauterine/intrapartum; yr: year; ICER: incremental cost-effectiveness ratio; YLS: years of life saved; CET: cost-effectiveness threshold; HR-HIVE: high-risk HIV-exposed infants; HIVE: all HIV-exposed infants; ALL: all live infants at birth.*

Pediatric HIV incidence is rounded to the nearest tenth of a percent. IU/IP HIV incidence is calculated based on the number of infants exposed to HIV at birth. Postnatal and total HIV incidence is calculated based on the number of infants ever exposed to HIV through 36 months of life. Undiscounted and discounted life expectancies are rounded to the nearest ten thousandth. Costs are rounded to the nearest dollar and are presented in 2020 USD. Discounted values are discounted at 3% per year. ICERs are rounded to the nearest dollar and are calculated using unrounded discounted life expectancy and discounted costs. The cost-effective bNAb strategy was the strategy that offered the greatest increase in overall population life expectancy while still having an ICER less than the cost-effectiveness threshold when compared to the next best performing, non-dominated strategy. * Indicates the cost-effective strategy using a cost-effectiveness threshold of 20% GDP per capita. † Indicates the cost-effective strategy using a cost-effectiveness threshold of 50% GDP per capita.

# **Supplementary Table 10.** One-way sensitivity analysis: bNAb cost (base case: $20/dose)

| **Country/strategy** | **Clinical outcomes** | | | **Lifetime efficacy and costs** | | | |
| --- | --- | --- | --- | --- | --- | --- | --- |
|  | **IU/IP cumulative HIV incidence (%)** | **Postnatal cumulative HIV incidence (%)** | **Total cumulative HIV incidence (%)** | **Undiscounted life expectancy (yrs)** | **Discounted life expectancy (yrs)** | **Discounted costs ($)** | **ICER ($/YLS)** |
| **Côte d’Ivoire [CET: ICER ≤ $465/YLS (20% GDP per capita), ICER ≤ $1163/YLS (50% GDP per capita)]** | | | | | | | |
| ***bNAb cost: $5/dose*** |  |  |  |  |  |  |  |
| Standard-of-care | 3·6 | 5·0 | 8·4 | 60·129 | 26·373 | 15 | Reference |
| HR-HIVE – 1 dose | 3·3 | 4·8 | 8·0 | 60·133 | 26·375 | 15 | dominated |
| HR-HIVE – 2 doses | 3·3 | 4·7 | 7·8 | 60·134 | 26·375 | 15 | dominated |
| HIVE – 1 dose | 3·3 | 4·7 | 7·8 | 60·135 | 26·375 | 14 | dominated |
| HR-HIVE – Extended | 3·3 | 4·3 | 7·5 | 60·136 | 26·376 | 14 | dominated |
| HIVE – 2 doses | 3·3 | 4·3 | 7·4 | 60·138 | 26·377 | 13 | dominated |
| ALL – 1 dose | 3·0 | 4·6 | 7·5 | 60·139 | 26·377 | 17 | dominated |
| ALL – 2 doses | 3·0 | 4·2 | 7·0 | 60·140 | 26·378 | 21 | dominated |
| HIVE – Extended | 3·3 | 3·2 | 6·3 | 60·146 | 26·379 | 11 | cost-saving*^,^† |
| ALL – 1 dose plus HIVE - Extended | 3·0 | 3·1 | 6·0 | 60·151 | 26·382 | 15 | 1673 |
| ALL - Extended | 3·0 | 2·7 | 5·5 | 60·156 | 26·383 | 31 | 14 322 |
| ***bNAb cost: $100/dose*** |  |  |  |  |  |  |  |
| Standard-of-care | 3·6 | 5·0 | 8·4 | 60·129 | 26·373 | 15 | Reference |
| HR-HIVE – 1 dose | 3·3 | 4·8 | 8·0 | 60·133 | 26·375 | 15 | dominated |
| HR-HIVE – 2 doses | 3·3 | 4·7 | 7·8 | 60·134 | 26·375 | 16 | dominated |
| HIVE – 1 dose | 3·3 | 4·7 | 7·8 | 60·135 | 26·375 | 15 | 400* |
| HR-HIVE – Extended | 3·3 | 4·3 | 7·5 | 60·136 | 26·376 | 16 | dominated |
| HIVE – 2 doses | 3·3 | 4·3 | 7·4 | 60·138 | 26·377 | 17 | dominated |
| ALL – 1 dose | 3·0 | 4·6 | 7·5 | 60·139 | 26·377 | 82 | dominated |
| ALL – 2 doses | 3·0 | 4·2 | 7·0 | 60·140 | 26·378 | 162 | dominated |
| HIVE – Extended | 3·3 | 3·2 | 6·3 | 60·146 | 26·379 | 19 | 874† |
| ALL – 1 dose plus HIVE - Extended | 3·0 | 3·1 | 6·0 | 60·151 | 26·382 | 86 | 31 377 |
| ALL - Extended | 3·0 | 2·7 | 5·5 | 60·156 | 26·383 | 410 | 289 474 |
| **South Africa [CET: ICER ≤ $1131/YLS (20% GDP per capita), ICER ≤ $2828/YLS (50% GDP per capita)]** | | | | | | | |
| ***bNAb cost: $5/dose*** |  |  |  |  |  |  |  |
| Standard-of-care | 2·3 | 2·2 | 4·4 | 68·924 | 28·499 | 112 | Reference |
| HR-HIVE – 1 dose | 2·1 | 2·1 | 4·0 | 68·979 | 28·518 | 114 | dominated |
| HR-HIVE – 2 doses | 2·1 | 2·1 | 4·0 | 68·986 | 28·521 | 114 | dominated |
| HR-HIVE – Extended | 2·1 | 2·0 | 3·9 | 68·995 | 28·523 | 113 | dominated |
| HIVE – 1 dose | 2·0 | 2·0 | 3·8 | 69·011 | 28·529 | 103 | dominated |
| HIVE – 2 doses | 2·0 | 1·8 | 3·6 | 69·045 | 28·541 | 99 | dominated |
| ALL – 1 dose | 1·8 | 1·8 | 3·5 | 69·063 | 28·548 | 105 | dominated |
| HIVE – Extended | 2·0 | 1·5 | 3·3 | 69·095 | 28·557 | 93 | cost-saving |
| ALL – 2 doses | 1·8 | 1·5 | 3·2 | 69·122 | 28·568 | 102 | dominated |
| ALL – 1 dose plus HIVE - Extended | 1·8 | 1·3 | 3·0 | 69·147 | 28·575 | 96 | dominated |
| ALL - Extended | 1·8 | 0·8 | 2·5 | 69·228 | 28·602 | 94 | 24*^,^† |

**Supplementary Table 10.** One-way sensitivity analysis: bNAb cost (base case: $20/dose) (cont.)

| **Country/strategy** | **Clinical outcomes** | | | **Lifetime efficacy and costs** | | | |
| --- | --- | --- | --- | --- | --- | --- | --- |
|  | **IU/IP cumulative HIV incidence (%)** | **Postnatal cumulative HIV incidence (%)** | **Total cumulative HIV incidence (%)** | **Undiscounted life expectancy (yrs)** | **Discounted life expectancy (yrs)** | **Discounted costs ($)** | **ICER ($/YLS)** |
| **South Africa [CET: ICER ≤ $1131/YLS (20% GDP per capita), ICER ≤ $2828/YLS (50% GDP per capita)]** | | | | | | | |
| ***bNAb cost: $100/dose*** |  |  |  |  |  |  |  |
| Standard-of-care | 2·3 | 2·2 | 4·4 | 68·924 | 28·499 | 112 | Reference |
| HR-HIVE – 1 dose | 2·1 | 2·1 | 4·0 | 68·979 | 28·518 | 117 | dominated |
| HR-HIVE – 2 doses | 2·1 | 2·1 | 4·0 | 68·986 | 28·521 | 119 | dominated |
| HR-HIVE – Extended | 2·1 | 2·0 | 3·9 | 68·995 | 28·523 | 121 | dominated |
| HIVE – 1 dose | 2·0 | 2·0 | 3·8 | 69·011 | 28·529 | 118 | 214 |
| HIVE – 2 doses | 2·0 | 1·8 | 3·6 | 69·045 | 28·541 | 127 | dominated |
| ALL – 1 dose | 1·8 | 1·8 | 3·5 | 69·063 | 28·548 | 172 | dominated |
| HIVE – Extended | 2·0 | 1·5 | 3·3 | 69·095 | 28·557 | 138 | 723*^,^† |
| ALL – 2 doses | 1·8 | 1·5 | 3·2 | 69·122 | 28·568 | 228 | dominated |
| ALL – 1 dose plus HIVE - Extended | 1·8 | 1·3 | 3·0 | 69·147 | 28·575 | 192 | 2918 |
| ALL - Extended | 1·8 | 0·8 | 2·5 | 69·228 | 28·602 | 350 | 5977 |
| **Zimbabwe [CET: ICER ≤ $243/YLS (20% GDP per capita), ICER ≤ $607/YLS (50% GDP per capita)]** | | | | | | | |
| ***bNAb cost: $5/dose*** |  |  |  |  |  |  |  |
| Standard-of-care | 4·3 | 5·6 | 9·5 | 68·321 | 28·034 | 72 | Reference |
| HR-HIVE – 1 dose | 3·7 | 5·3 | 8·6 | 68·366 | 28·050 | 70 | dominated |
| HR-HIVE – 2 doses | 3·7 | 5·2 | 8·5 | 68·373 | 28·053 | 69 | dominated |
| HIVE – 1 dose | 3·7 | 5·2 | 8·4 | 68·380 | 28·055 | 65 | dominated |
| HR-HIVE – Extended | 3·7 | 4·9 | 8·2 | 68·387 | 28·057 | 67 | dominated |
| ALL – 1 dose | 3·4 | 4·9 | 7·9 | 68·400 | 28·062 | 67 | dominated |
| HIVE – 2 doses | 3·7 | 4·8 | 8·0 | 68·402 | 28·063 | 63 | dominated |
| ALL – 2 doses | 3·4 | 4·3 | 7·4 | 68·438 | 28·074 | 67 | dominated |
| HIVE – Extended | 3·7 | 3·6 | 6·9 | 68·458 | 28·081 | 55 | cost-saving |
| ALL – 1 dose plus HIVE - Extended | 3·4 | 3·4 | 6·5 | 68·478 | 28·088 | 56 | 160*^,^† |
| ALL - Extended | 3·4 | 2·6 | 5·7 | 68·517 | 28·101 | 67 | 791 |
| ***bNAb cost: $100/dose*** |  |  |  |  |  |  |  |
| Standard-of-care | 4·3 | 5·6 | 9·5 | 68·321 | 28·034 | 72 | Reference |
| HR-HIVE – 1 dose | 3·7 | 5·3 | 8·6 | 68·366 | 28·050 | 72 | 54* |
| HR-HIVE – 2 doses | 3·7 | 5·2 | 8·5 | 68·373 | 28·053 | 74 | dominated |
| HIVE – 1 dose | 3·7 | 5·2 | 8·4 | 68·380 | 28·055 | 74 | 295† |
| HR-HIVE – Extended | 3·7 | 4·9 | 8·2 | 68·387 | 28·057 | 78 | dominated |
| ALL – 1 dose | 3·4 | 4·9 | 7·9 | 68·400 | 28·062 | 146 | dominated |
| HIVE – 2 doses | 3·7 | 4·8 | 8·0 | 68·402 | 28·063 | 80 | dominated |
| ALL – 2 doses | 3·4 | 4·3 | 7·4 | 68·438 | 28·074 | 228 | dominated |
| HIVE – Extended | 3·7 | 3·6 | 6·9 | 68·458 | 28·081 | 93 | 756 |
| ALL – 1 dose plus HIVE - Extended | 3·4 | 3·4 | 6·5 | 68·478 | 28·088 | 165 | 9833 |
| ALL - Extended | 3·4 | 2·6 | 5·7 | 68·517 | 28·101 | 488 | 24 338 |

*bNAb: broadly neutralizing antibody; IU/IP: intrauterine/intrapartum; yr: year; ICER: incremental cost-effectiveness ratio; YLS: years of life saved; CET: cost-effectiveness threshold; HR-HIVE: high-risk HIV-exposed infants; HIVE: all HIV-exposed infants; ALL: all live infants at birth.*

Pediatric HIV incidence is rounded to the nearest tenth of a percent. IU/IP HIV incidence is calculated based on the number of infants exposed to HIV at birth. Postnatal and total HIV incidence is calculated based on the number of infants ever exposed to HIV through 36 months of life. Undiscounted and discounted life expectancies are rounded to the nearest ten thousandth. Costs are rounded to the nearest dollar and are presented in 2020 USD. Discounted values are discounted at 3% per year. ICERs are rounded to the nearest dollar and are calculated using unrounded discounted life expectancy and discounted costs. The cost-effective bNAb strategy was the strategy that offered the greatest increase in overall population life expectancy while still having an ICER less than the cost-effectiveness threshold when compared to the next best performing, non-dominated strategy. * Indicates the cost-effective strategy using a cost-effectiveness threshold of 20% GDP per capita. † Indicates the cost-effective strategy using a cost-effectiveness threshold of 50% GDP per capita.

# **Supplementary Table 11.** One-way sensitivity analysis: bNAb effect duration (base case: 3 months)

| **Country/strategy** | **Clinical outcomes** | | | **Lifetime efficacy and costs** | | | |
| --- | --- | --- | --- | --- | --- | --- | --- |
|  | **IU/IP cumulative HIV incidence (%)** | **Postnatal cumulative HIV incidence (%)** | **Total cumulative HIV incidence (%)** | **Undiscounted life expectancy (yrs)** | **Discounted life expectancy (yrs)** | **Discounted costs ($)** | **ICER ($/YLS)** |
| **Côte d’Ivoire [CET: ICER ≤ $465/YLS (20% GDP per capita), ICER ≤ $1163/YLS (50% GDP per capita)]** | | | | | | | |
| ***bNAb effect duration: 1 month*** |  |  |  |  |  |  |  |
| Standard-of-care | 3·6 | 5·0 | 8·4 | 60·129 | 26·373 | 15 | Reference |
| HR-HIVE – 1 dose | 3·3 | 4·9 | 8·0 | 60·132 | 26·374 | 15 | dominated |
| HR-HIVE – 2 doses | 3·3 | 4·9 | 8·0 | 60·133 | 26·375 | 15 | dominated |
| HIVE – 1 dose | 3·3 | 4·9 | 7·9 | 60·133 | 26·375 | 14 | cost-saving |
| HIVE – 2 doses | 3·3 | 4·8 | 7·8 | 60·134 | 26·375 | 15 | dominated |
| HR-HIVE – Extended | 3·3 | 4·4 | 7·5 | 60·136 | 26·376 | 15 | dominated |
| ALL – 1 dose | 3·0 | 4·9 | 7·7 | 60·137 | 26·377 | 28 | dominated |
| ALL – 2 doses | 3·0 | 4·7 | 7·6 | 60·140 | 26·378 | 45 | dominated |
| HIVE – Extended | 3·3 | 3·2 | 6·3 | 60·145 | 26·379 | 16 | 267*^,^† |
| ALL – 1 dose plus HIVE - Extended | 3·0 | 3·2 | 6·1 | 60·150 | 26·381 | 29 | 7052 |
| ALL - Extended | 3·0 | 2·7 | 5·6 | 60·158 | 26·384 | 235 | 81 484 |
| ***bNAb effect duration: 6 months*** |  |  |  |  |  |  |  |
| Standard-of-care | 3·6 | 5·0 | 8·4 | 60·129 | 26·373 | 15 | Reference |
| HR-HIVE – 1 dose | 3·3 | 4·7 | 7·8 | 60·134 | 26·375 | 15 | dominated |
| HR-HIVE – 2 doses | 3·3 | 4·5 | 7·6 | 60·135 | 26·376 | 14 | dominated |
| HR-HIVE – Extended | 3·3 | 4·3 | 7·5 | 60·136 | 26·376 | 14 | dominated |
| HIVE – 1 dose | 3·3 | 4·4 | 7·4 | 60·137 | 26·376 | 14 | dominated |
| HIVE – 2 doses | 3·3 | 3·7 | 6·7 | 60·142 | 26·378 | 13 | dominated |
| ALL – 1 dose | 3·0 | 4·2 | 7·1 | 60·143 | 26·379 | 27 | dominated |
| HIVE – Extended | 3·3 | 3·1 | 6·2 | 60·146 | 26·379 | 12 | cost-saving*^,^† |
| ALL – 2 doses | 3·0 | 3·3 | 6·2 | 60·145 | 26·381 | 40 | dominated |
| ALL – 1 dose plus HIVE - Extended | 3·0 | 3·0 | 5·9 | 60·151 | 26·382 | 25 | 5467 |
| ALL - Extended | 3·0 | 2·6 | 5·5 | 60·158 | 26·383 | 55 | 19 636 |
| **South Africa [CET: ICER ≤ $1131/YLS (20% GDP per capita), ICER ≤ $2828/YLS (50% GDP per capita)]** | | | | | | | |
| ***bNAb effect duration: 1 month*** |  |  |  |  |  |  |  |
| Standard-of-care | 2·3 | 2·2 | 4·4 | 68·924 | 28·499 | 112 | Reference |
| HR-HIVE – 1 dose | 2·1 | 2·1 | 4·1 | 68·970 | 28·515 | 116 | dominated |
| HR-HIVE – 2 doses | 2·1 | 2·1 | 4·0 | 68·976 | 28·517 | 116 | dominated |
| HIVE – 1 dose | 2·0 | 2·1 | 4·0 | 68·985 | 28·520 | 108 | cost-saving |
| HR-HIVE – Extended | 2·1 | 2·0 | 3·9 | 68·995 | 28·523 | 117 | dominated |
| HIVE – 2 doses | 2·0 | 2·1 | 3·9 | 68·997 | 28·525 | 110 | dominated |
| ALL – 1 dose | 1·8 | 2·1 | 3·8 | 69·011 | 28·529 | 121 | dominated |
| ALL – 2 doses | 1·8 | 1·9 | 3·6 | 69·038 | 28·539 | 133 | dominated |
| HIVE – Extended | 2·0 | 1·5 | 3·3 | 69·093 | 28·557 | 116 | 222* |
| ALL – 1 dose plus HIVE - Extended | 1·8 | 1·4 | 3·1 | 69·119 | 28·566 | 130 | 1462† |
| ALL - Extended | 1·8 | 0·8 | 2·5 | 69·221 | 28·600 | 227 | 2861 |

**Supplementary Table 11.** One-way sensitivity analysis: bNAb effect duration (base case: 3 months) (cont.)

| **Country/strategy** | **Clinical outcomes** | | | **Lifetime efficacy and costs** | | | |
| --- | --- | --- | --- | --- | --- | --- | --- |
|  | **IU/IP cumulative HIV incidence (%)** | **Postnatal cumulative HIV incidence (%)** | **Total cumulative HIV incidence (%)** | **Undiscounted life expectancy (yrs)** | **Discounted life expectancy (yrs)** | **Discounted costs ($)** | **ICER ($/YLS)** |
| **South Africa [CET: ICER ≤ $1131/YLS (20% GDP per capita), ICER ≤ $2828/YLS (50% GDP per capita)]** | | | | | | | |
| ***bNAb effect duration: 6 months*** |  |  |  |  |  |  |  |
| Standard-of-care | 2·3 | 2·2 | 4·4 | 68·924 | 28·499 | 112 | Reference |
| HR-HIVE – 1 dose | 2·1 | 2·1 | 4·0 | 68·986 | 28·520 | 114 | dominated |
| HR-HIVE – 2 doses | 2·1 | 2·0 | 3·9 | 68·994 | 28·523 | 113 | dominated |
| HR-HIVE – Extended | 2·1 | 2·0 | 3·9 | 68·995 | 28·523 | 113 | dominated |
| HIVE – 1 dose | 2·0 | 1·8 | 3·6 | 69·044 | 28·541 | 101 | dominated |
| HIVE – 2 doses | 2·0 | 1·5 | 3·4 | 69·086 | 28·554 | 97 | dominated |
| HIVE – Extended | 2·0 | 1·5 | 3·3 | 69·097 | 28·558 | 96 | cost-saving |
| ALL – 1 dose | 1·8 | 1·5 | 3·2 | 69·119 | 28·567 | 109 | dominated |
| ALL – 1 dose plus HIVE - Extended | 1·8 | 1·1 | 2·8 | 69·171 | 28·584 | 105 | dominated |
| ALL – 2 doses | 1·8 | 1·0 | 2·7 | 69·200 | 28·593 | 110 | dominated |
| ALL - Extended | 1·8 | 0·8 | 2·5 | 69·231 | 28·603 | 111 | 322*^,^† |
| **Zimbabwe [CET: ICER ≤ $243/YLS (20% GDP per capita), ICER ≤ $607/YLS (50% GDP per capita)]** | | | | | | | |
| ***bNAb effect duration: 1 month*** |  |  |  |  |  |  |  |
| Standard-of-care | 4·3 | 5·6 | 9·5 | 68·321 | 28·034 | 72 | Reference |
| HR-HIVE – 1 dose | 3·7 | 5·5 | 8·8 | 68·356 | 28·046 | 72 | dominated |
| HR-HIVE – 2 doses | 3·7 | 5·4 | 8·7 | 68·361 | 28·048 | 72 | dominated |
| HIVE – 1 dose | 3·7 | 5·5 | 8·7 | 68·361 | 28·048 | 69 | cost-saving |
| ALL – 1 dose | 3·4 | 5·4 | 8·4 | 68·370 | 28·051 | 82 | dominated |
| HIVE – 2 doses | 3·7 | 5·3 | 8·6 | 68·370 | 28·051 | 70 | dominated |
| HR-HIVE – Extended | 3·7 | 4·9 | 8·2 | 68·388 | 28·057 | 73 | dominated |
| ALL – 2 doses | 3·4 | 5·1 | 8·2 | 68·392 | 28·058 | 99 | dominated |
| HIVE – Extended | 3·7 | 3·6 | 6·9 | 68·457 | 28·080 | 76 | 223*^,^† |
| ALL – 1 dose plus HIVE - Extended | 3·4 | 3·6 | 6·7 | 68·466 | 28·083 | 89 | 4366 |
| ALL - Extended | 3·4 | 2·6 | 5·7 | 68·511 | 28·101 | 294 | 11 905 |
| ***bNAb effect duration: 6 months*** |  |  |  |  |  |  |  |
| Standard-of-care | 4·3 | 5·6 | 9·5 | 68·321 | 28·034 | 72 | Reference |
| HR-HIVE – 1 dose | 3·7 | 5·2 | 8·5 | 68·373 | 28·052 | 70 | dominated |
| HR-HIVE – 2 doses | 3·7 | 5·0 | 8·3 | 68·382 | 28·055 | 69 | dominated |
| HR-HIVE – Extended | 3·7 | 4·9 | 8·2 | 68·387 | 28·057 | 68 | dominated |
| HIVE – 1 dose | 3·7 | 4·8 | 8·0 | 68·401 | 28·062 | 64 | dominated |
| ALL – 1 dose | 3·4 | 4·4 | 7·4 | 68·430 | 28·072 | 76 | dominated |
| HIVE – 2 doses | 3·7 | 4·1 | 7·4 | 68·436 | 28·074 | 60 | dominated |
| HIVE – Extended | 3·7 | 3·6 | 6·9 | 68·459 | 28·081 | 58 | cost-saving*^,^† |
| ALL – 2 doses | 3·4 | 3·3 | 6·4 | 68·487 | 28·090 | 83 | dominated |
| ALL – 1 dose plus HIVE - Extended | 3·4 | 3·2 | 6·3 | 68·488 | 28·091 | 69 | 1108 |
| ALL - Extended | 3·4 | 2·5 | 5·6 | 68·520 | 28·102 | 94 | 2223 |

*bNAb: broadly neutralizing antibody; IU/IP: intrauterine/intrapartum; yr: year; ICER: incremental cost-effectiveness ratio; YLS: years of life saved; CET: cost-effectiveness threshold; HR-HIVE: high-risk HIV-exposed infants; HIVE: all HIV-exposed infants; ALL: all live infants at birth.*

Pediatric HIV incidence is rounded to the nearest tenth of a percent. IU/IP HIV incidence is calculated based on the number of infants exposed to HIV at birth. Postnatal and total HIV incidence is calculated based on the number of infants ever exposed to HIV through 36 months of life. Undiscounted and discounted life expectancies are rounded to the nearest ten thousandth. Costs are rounded to the nearest dollar and are presented in 2020 USD. Discounted values are discounted at 3% per year. ICERs are rounded to the nearest dollar and are calculated using unrounded discounted life expectancy and discounted costs. The cost-effective bNAb strategy was the strategy that offered the greatest increase in overall population life expectancy while still having an ICER less than the cost-effectiveness threshold when compared to the next best performing, non-dominated strategy. * Indicates the cost-effective strategy using a cost-effectiveness threshold of 20% GDP per capita. † Indicates the cost-effective strategy using a cost-effectiveness threshold of 50% GDP per capita.

# **Supplementary Table 12.** One-way sensitivity analysis: bNAb uptake

| **Country/strategy** | **Clinical outcomes** | | | **Lifetime efficacy and costs** | | | |
| --- | --- | --- | --- | --- | --- | --- | --- |
|  | **IU/IP cumulative HIV incidence (%)** | **Postnatal cumulative HIV incidence (%)** | **Total cumulative HIV incidence (%)** | **Undiscounted life expectancy (yrs)** | **Discounted life expectancy (yrs)** | **Discounted costs ($)** | **ICER ($/YLS)** |
| **Côte d’Ivoire [CET: ICER ≤ $465/YLS (20% GDP per capita), ICER ≤ $1163/YLS (50% GDP per capita)]** | | | | | | | |
| ***56% uptake at all time points*** |  |  |  |  |  |  |  |
| Standard-of-care | 3·6 | 5·0 | 8·4 | 60·129 | 26·373 | 15 | Reference |
| HR-HIVE – 1 dose | 3·4 | 4·9 | 8·0 | 60·132 | 26·374 | 15 | dominated |
| HR-HIVE – 2 doses | 3·4 | 4·8 | 7·9 | 60·133 | 26·375 | 15 | dominated |
| HIVE – 1 dose | 3·3 | 4·8 | 7·9 | 60·133 | 26·375 | 14 | dominated |
| HR-HIVE – Extended | 3·4 | 4·5 | 7·7 | 60·135 | 26·375 | 15 | dominated |
| HIVE – 2 doses | 3·3 | 4·5 | 7·6 | 60·136 | 26·376 | 14 | dominated |
| ALL – 1 dose | 3·1 | 4·7 | 7·7 | 60·138 | 26·376 | 25 | dominated |
| ALL – 2 doses | 3·1 | 4·4 | 7·3 | 60·142 | 26·378 | 35 | dominated |
| HIVE – Extended | 3·3 | 3·6 | 6·7 | 60·142 | 26·378 | 13 | cost-saving*^,^† |
| ALL – 1 dose plus HIVE - Extended | 3·1 | 3·5 | 6·5 | 60·146 | 26·380 | 24 | 6745 |
| ALL - Extended | 3·1 | 3·2 | 6·2 | 60·153 | 26·381 | 75 | 38 825 |
| ***83% uptake at all time points*** |  |  |  |  |  |  |  |
| Standard-of-care | 3·6 | 5·0 | 8·4 | 60·129 | 26·373 | 15 | Reference |
| HR-HIVE – 1 dose | 3·3 | 4·8 | 7·9 | 60·134 | 26·375 | 15 | dominated |
| HR-HIVE – 2 doses | 3·3 | 4·7 | 7·7 | 60·135 | 26·375 | 15 | dominated |
| HIVE – 1 dose | 3·2 | 4·7 | 7·7 | 60·136 | 26·376 | 14 | dominated |
| HR-HIVE – Extended | 3·3 | 4·2 | 7·3 | 60·137 | 26·376 | 14 | dominated |
| HIVE – 2 doses | 3·2 | 4·3 | 7·3 | 60·139 | 26·377 | 14 | dominated |
| ALL – 1 dose | 2·9 | 4·6 | 7·3 | 60·141 | 26·378 | 30 | dominated |
| ALL – 2 doses | 2·9 | 4·1 | 6·8 | 60·143 | 26·379 | 45 | dominated |
| HIVE – Extended | 3·2 | 2·9 | 5·9 | 60·148 | 26·380 | 12 | cost-saving*^,^† |
| ALL – 1 dose plus HIVE - Extended | 2·9 | 2·8 | 5·6 | 60·154 | 26·383 | 28 | 6494 |
| ALL - Extended | 2·9 | 2·3 | 5·1 | 60·158 | 26·384 | 103 | 65 508 |
| **South Africa [CET: ICER ≤ $1131/YLS (20% GDP per capita), ICER ≤ $2828/YLS (50% GDP per capita)]** | | | | | | | |
| ***85% uptake at all time points*** |  |  |  |  |  |  |  |
| Standard-of-care | 2·3 | 2·2 | 4·4 | 68·924 | 28·499 | 112 | Reference |
| HR-HIVE – 1 dose | 2·1 | 2·1 | 4·1 | 68·973 | 28·516 | 116 | dominated |
| HR-HIVE – 2 doses | 2·1 | 2·1 | 4·0 | 68·980 | 28·518 | 115 | dominated |
| HR-HIVE – Extended | 2·1 | 2·0 | 4·0 | 68·987 | 28·521 | 115 | dominated |
| HIVE – 1 dose | 2·0 | 2·0 | 3·9 | 69·002 | 28·526 | 106 | dominated |
| HIVE – 2 doses | 2·0 | 1·8 | 3·7 | 69·032 | 28·537 | 105 | dominated |
| ALL – 1 dose | 1·9 | 1·8 | 3·6 | 69·050 | 28·543 | 115 | dominated |
| HIVE – Extended | 2·0 | 1·5 | 3·4 | 69·078 | 28·552 | 102 | cost-saving |
| ALL – 2 doses | 1·9 | 1·5 | 3·3 | 69·098 | 28·560 | 122 | dominated |
| ALL – 1 dose plus HIVE - Extended | 1·9 | 1·4 | 3·1 | 69·126 | 28·568 | 111 | 589 |
| ALL - Extended | 1·9 | 0·9 | 2·7 | 69·201 | 28·592 | 134 | 930*^,^† |

**Supplementary Table 12.** One-way sensitivity analysis: bNAb uptake (cont.)

| **Country/strategy** | **Clinical outcomes** | | | **Lifetime efficacy and costs** | | | |
| --- | --- | --- | --- | --- | --- | --- | --- |
|  | **IU/IP cumulative HIV incidence (%)** | **Postnatal cumulative HIV incidence (%)** | **Total cumulative HIV incidence (%)** | **Undiscounted life expectancy (yrs)** | **Discounted life expectancy (yrs)** | **Discounted costs ($)** | **ICER ($/YLS)** |
| **South Africa [CET: ICER ≤ $1131/YLS (20% GDP per capita), ICER ≤ $2828/YLS (50% GDP per capita)]** | | | | | | | |
| ***96% uptake at all time points*** |  |  |  |  |  |  |  |
| Standard-of-care | 2·3 | 2·2 | 4·4 | 68·924 | 28·499 | 112 | Reference |
| HR-HIVE – 1 dose | 2·1 | 2·1 | 4·0 | 68·979 | 28·518 | 115 | dominated |
| HR-HIVE – 2 doses | 2·1 | 2·1 | 4·0 | 68·986 | 28·521 | 115 | dominated |
| HR-HIVE – Extended | 2·1 | 2·0 | 3·9 | 68·996 | 28·524 | 114 | dominated |
| HIVE – 1 dose | 2·0 | 2·0 | 3·8 | 69·011 | 28·529 | 105 | dominated |
| HIVE – 2 doses | 2·0 | 1·8 | 3·6 | 69·045 | 28·541 | 104 | dominated |
| ALL – 1 dose | 1·8 | 1·8 | 3·5 | 69·063 | 28·548 | 116 | dominated |
| HIVE – Extended | 2·0 | 1·5 | 3·3 | 69·098 | 28·558 | 100 | cost-saving |
| ALL – 2 doses | 1·8 | 1·4 | 3·1 | 69·122 | 28·568 | 122 | dominated |
| ALL – 1 dose plus HIVE - Extended | 1·8 | 1·3 | 3·0 | 69·150 | 28·576 | 111 | 606 |
| ALL - Extended | 1·8 | 0·8 | 2·5 | 69·235 | 28·604 | 136 | 891*^,^† |
| **Zimbabwe [CET: ICER ≤ $243/YLS (20% GDP per capita), ICER ≤ $607/YLS (50% GDP per capita)]** | | | | | | | |
| ***71% uptake at all time points*** |  |  |  |  |  |  |  |
| Standard-of-care | 4·3 | 5·6 | 9·5 | 68·321 | 28·034 | 72 | Reference |
| HR-HIVE – 1 dose | 3·8 | 5·4 | 8·8 | 68·358 | 28·047 | 71 | dominated |
| HR-HIVE – 2 doses | 3·8 | 5·3 | 8·7 | 68·363 | 28·049 | 71 | dominated |
| HIVE – 1 dose | 3·8 | 5·2 | 8·6 | 68·370 | 28·051 | 68 | dominated |
| HR-HIVE – Extended | 3·8 | 5·0 | 8·4 | 68·375 | 28·053 | 70 | dominated |
| ALL – 1 dose | 3·6 | 5·0 | 8·2 | 68·381 | 28·056 | 78 | dominated |
| HIVE – 2 doses | 3·8 | 4·9 | 8·3 | 68·387 | 28·057 | 67 | dominated |
| ALL – 2 doses | 3·6 | 4·6 | 7·8 | 68·419 | 28·067 | 89 | dominated |
| HIVE – Extended | 3·8 | 4·0 | 7·3 | 68·433 | 28·072 | 63 | cost-saving*^,^† |
| ALL – 1 dose plus HIVE - Extended | 3·6 | 3·7 | 7·0 | 68·445 | 28·077 | 73 | 2444 |
| ALL - Extended | 3·6 | 3·1 | 6·3 | 68·488 | 28·090 | 124 | 3706 |
| ***92% uptake at all time points*** |  |  |  |  |  |  |  |
| Standard-of-care | 4·3 | 5·6 | 9·5 | 68·321 | 28·034 | 72 | Reference |
| HR-HIVE – 1 dose | 3·7 | 5·3 | 8·6 | 68·369 | 28·051 | 70 | dominated |
| HR-HIVE – 2 doses | 3·7 | 5·2 | 8·4 | 68·377 | 28·054 | 70 | dominated |
| HIVE – 1 dose | 3·6 | 5·1 | 8·3 | 68·384 | 28·056 | 66 | dominated |
| HR-HIVE – Extended | 3·7 | 4·8 | 8·1 | 68·392 | 28·059 | 68 | dominated |
| HIVE – 2 doses | 3·6 | 4·7 | 7·9 | 68·406 | 28·064 | 65 | dominated |
| ALL – 1 dose | 3·4 | 4·8 | 7·8 | 68·409 | 28·065 | 80 | dominated |
| ALL – 2 doses | 3·4 | 4·3 | 7·3 | 68·441 | 28·076 | 93 | dominated |
| HIVE – Extended | 3·6 | 3·4 | 6·7 | 68·469 | 28·084 | 60 | cost-saving*^,^† |
| ALL – 1 dose plus HIVE - Extended | 3·4 | 3·2 | 6·2 | 68·494 | 28·093 | 74 | 1545 |
| ALL - Extended | 3·4 | 2·3 | 5·3 | 68·535 | 28·107 | 140 | 4822 |

*bNAb: broadly neutralizing antibody; IU/IP: intrauterine/intrapartum; yr: year; ICER: incremental cost-effectiveness ratio; YLS: years of life saved; CET: cost-effectiveness threshold; HR-HIVE: high-risk HIV-exposed infants; HIVE: all HIV-exposed infants; ALL: all live infants at birth.*

The setting-specific base case uptake is specified in Supplementary Table 2. Pediatric HIV incidence is rounded to the nearest tenth of a percent. IU/IP HIV incidence is calculated based on the number of infants exposed to HIV at birth. Postnatal and total HIV incidence is calculated based on the number of infants ever exposed to HIV through 36 months of life. Undiscounted and discounted life expectancies are rounded to the nearest ten thousandth. Costs are rounded to the nearest dollar and are presented in 2020 USD. Discounted values are discounted at 3% per year. ICERs are rounded to the nearest dollar and are calculated using unrounded discounted life expectancy and discounted costs. The cost-effective bNAb strategy was the strategy that offered the greatest increase in overall population life expectancy while still having an ICER less than the cost-effectiveness threshold when compared to the next best performing, non-dominated strategy. * Indicates the cost-effective strategy using a cost-effectiveness threshold of 20% GDP per capita. † Indicates the cost-effective strategy using a cost-effectiveness threshold of 50% GDP per capita.

# **Supplementary Table 13.** One-way sensitivity analysis: proportion of mothers on antiretroviral therapy during pregnancy

| **Country/strategy** | **Clinical outcomes** | | | **Lifetime efficacy and costs** | | | |
| --- | --- | --- | --- | --- | --- | --- | --- |
|  | **IU/IP cumulative HIV incidence (%)** | **Postnatal cumulative HIV incidence (%)** | **Total cumulative HIV incidence (%)** | **Undiscounted life expectancy (yrs)** | **Discounted life expectancy (yrs)** | **Discounted costs ($)** | **ICER ($/YLS)** |
| **Côte d’Ivoire [CET: ICER ≤ $465/YLS (20% GDP per capita), ICER ≤ $1163/YLS (50% GDP per capita)]** | | | | | | | |
| ***Proportion of mothers on ART during pregnancy: 70%*** | |  |  |  |  |  |  |
| Standard-of-care | 7·3 | 5·4 | 12·2 | 60·081 | 26·353 | 42 | Reference |
| HR-HIVE – 1 dose | 6·4 | 5·2 | 11·1 | 60·090 | 26·357 | 39 | dominated |
| HIVE – 1 dose | 6·3 | 5·1 | 11·0 | 60·091 | 26·357 | 38 | dominated |
| HR-HIVE – 2 doses | 6·4 | 4·9 | 10·8 | 60·092 | 26·357 | 39 | dominated |
| HIVE – 2 doses | 6·3 | 4·6 | 10·5 | 60·094 | 26·359 | 37 | dominated |
| HR-HIVE – Extended | 6·4 | 4·3 | 10·3 | 60·095 | 26·359 | 37 | dominated |
| ALL – 1 dose | 6·1 | 5·0 | 10·6 | 60·096 | 26·359 | 52 | dominated |
| ALL – 2 doses | 6·1 | 4·4 | 10·1 | 60·097 | 26·360 | 66 | dominated |
| HIVE – Extended | 6·3 | 3·4 | 9·4 | 60·102 | 26·361 | 33 | cost-saving*^,^† |
| ALL – 1 dose plus HIVE - Extended | 6·1 | 3·3 | 9·1 | 60·107 | 26·363 | 47 | 6244 |
| ALL - Extended | 6·1 | 2·9 | 8·6 | 60·113 | 26·365 | 112 | 57 665 |
| ***Proportion of mothers on ART during pregnancy: 100%*** | |  |  |  |  |  |  |
| Standard-of-care | 3·0 | 4·9 | 7·7 | 60·138 | 26·377 | 14 | Reference |
| HR-HIVE – 1 dose | 2·8 | 4·8 | 7·4 | 60·141 | 26·378 | 14 | dominated |
| HR-HIVE – 2 doses | 2·8 | 4·7 | 7·3 | 60·142 | 26·378 | 14 | dominated |
| HIVE – 1 dose | 2·7 | 4·7 | 7·2 | 60·143 | 26·379 | 13 | dominated |
| HR-HIVE – Extended | 2·8 | 4·3 | 7·0 | 60·144 | 26·379 | 14 | dominated |
| HIVE – 2 doses | 2·7 | 4·3 | 6·8 | 60·146 | 26·380 | 13 | dominated |
| ALL – 1 dose | 2·5 | 4·6 | 6·9 | 60·148 | 26·381 | 27 | dominated |
| ALL – 2 doses | 2·5 | 4·1 | 6·5 | 60·148 | 26·381 | 42 | dominated |
| HIVE – Extended | 2·7 | 3·1 | 5·7 | 60·154 | 26·383 | 12 | cost-saving*^,^† |
| ALL – 1 dose plus HIVE - Extended | 2·5 | 3·1 | 5·4 | 60·159 | 26·385 | 25 | 6242 |
| ALL - Extended | 2·5 | 2·6 | 5·0 | 60·164 | 26·386 | 90 | 57 888 |
| **South Africa [CET: ICER ≤ $1131/YLS (20% GDP per capita), ICER ≤ $2828/YLS (50% GDP per capita)]** | | | | | | | |
| ***Proportion of mothers on ART during pregnancy: 70%*** | |  |  |  |  |  |  |
| Standard-of-care | 8·1 | 2·6 | 10·1 | 67·590 | 28·013 | 232 | Reference |
| HR-HIVE – 1 dose | 6·4 | 2·3 | 8·3 | 67·824 | 28·091 | 209 | dominated |
| HIVE – 1 dose | 6·3 | 2·3 | 8·1 | 67·844 | 28·098 | 199 | dominated |
| HR-HIVE – 2 doses | 6·4 | 2·2 | 8·1 | 67·848 | 28·099 | 207 | dominated |
| HR-HIVE – Extended | 6·4 | 2·0 | 8·0 | 67·869 | 28·106 | 206 | dominated |
| HIVE – 2 doses | 6·3 | 2·0 | 7·9 | 67·884 | 28·112 | 196 | dominated |
| ALL – 1 dose | 6·2 | 2·0 | 7·8 | 67·895 | 28·116 | 209 | dominated |
| HIVE – Extended | 6·3 | 1·7 | 7·6 | 67·931 | 28·127 | 193 | cost-saving |
| ALL – 2 doses | 6·2 | 1·6 | 7·4 | 67·961 | 28·139 | 214 | dominated |
| ALL – 1 dose plus HIVE - Extended | 6·2 | 1·5 | 7·2 | 67·982 | 28·145 | 204 | 575 |
| ALL - Extended | 6·2 | 0·9 | 6·7 | 68·066 | 28·172 | 227 | 850*^,^† |

**Supplementary Table 13.** One-way sensitivity analysis: proportion of mothers on antiretroviral therapy during pregnancy (cont.)

| **Country/strategy** | **Clinical outcomes** | | | **Lifetime efficacy and costs** | | | |
| --- | --- | --- | --- | --- | --- | --- | --- |
|  | **IU/IP cumulative HIV incidence (%)** | **Postnatal cumulative HIV incidence (%)** | **Total cumulative HIV incidence (%)** | **Undiscounted life expectancy (yrs)** | **Discounted life expectancy (yrs)** | **Discounted costs ($)** | **ICER ($/YLS)** |
| **South Africa [CET: ICER ≤ $1131/YLS (20% GDP per capita), ICER ≤ $2828/YLS (50% GDP per capita)]** | | | | | | | |
| ***Proportion of mothers on ART during pregnancy: 100%*** | |  |  |  |  |  |  |
| Standard-of-care | 2·0 | 2·2 | 4·0 | 69·018 | 28·534 | 104 | Reference |
| HR-HIVE – 1 dose | 1·8 | 2·1 | 3·7 | 69·061 | 28·548 | 109 | dominated |
| HR-HIVE – 2 doses | 1·8 | 2·0 | 3·7 | 69·067 | 28·550 | 108 | dominated |
| HR-HIVE – Extended | 1·8 | 2·0 | 3·7 | 69·075 | 28·553 | 108 | dominated |
| HIVE –1 dose | 1·7 | 2·0 | 3·5 | 69·094 | 28·560 | 99 | dominated |
| HIVE – 2 doses | 1·7 | 1·8 | 3·3 | 69·127 | 28·571 | 98 | dominated |
| ALL – 1 dose | 1·5 | 1·8 | 3·2 | 69·145 | 28·578 | 110 | dominated |
| HIVE – Extended | 1·7 | 1·5 | 3·0 | 69·177 | 28·588 | 94 | cost-saving |
| ALL – 2 doses | 1·5 | 1·4 | 2·9 | 69·204 | 28·598 | 116 | dominated |
| ALL – 1 dose plus HIVE - Extended | 1·5 | 1·3 | 2·7 | 69·229 | 28·606 | 105 | 606 |
| ALL - Extended | 1·5 | 0·8 | 2·2 | 69·310 | 28·632 | 129 | 884*^,^† |
| **Zimbabwe [CET: ICER ≤ $243/YLS (20% GDP per capita), ICER ≤ $607/YLS (50% GDP per capita)]** | | | | | | | |
| ***Proportion of mothers on ART during pregnancy: 70%*** | |  |  |  |  |  |  |
| Standard-of-care | 7·8 | 6·1 | 12·9 | 68·038 | 27·928 | 93 | Reference |
| HR-HIVE – 1 dose | 6·5 | 5·6 | 11·3 | 68·119 | 27·957 | 88 | dominated |
| HIVE – 1 dose | 6·4 | 5·5 | 11·1 | 68·130 | 27·960 | 84 | dominated |
| HR-HIVE – 2 doses | 6·5 | 5·3 | 11·0 | 68·133 | 27·961 | 87 | dominated |
| ALL– 1 dose | 6·2 | 5·2 | 10·6 | 68·150 | 27·967 | 97 | dominated |
| HR-HIVE – Extended | 6·5 | 4·8 | 10·6 | 68·156 | 27·969 | 85 | dominated |
| HIVE – 2 doses | 6·4 | 5·0 | 10·7 | 68·154 | 27·969 | 83 | dominated |
| ALL – 2 doses | 6·2 | 4·6 | 10·0 | 68·191 | 27·980 | 110 | dominated |
| HIVE – Extended | 6·4 | 3·9 | 9·6 | 68·208 | 27·986 | 78 | cost-saving*^,^† |
| ALL – 1 dose plus HIVE - Extended | 6·2 | 3·6 | 9·1 | 68·228 | 27·993 | 91 | 1739 |
| ALL - Extended | 6·2 | 2·7 | 8·3 | 68·267 | 28·007 | 151 | 4466 |
| ***Proportion of mothers on ART during pregnancy: 100%*** | |  |  |  |  |  |  |
| Standard-of-care | 2·7 | 5·4 | 7·8 | 68·461 | 28·087 | 61 | Reference |
| HR-HIVE – 1 dose | 2·5 | 5·2 | 7·4 | 68·488 | 28·096 | 62 | dominated |
| HR-HIVE – 2 doses | 2·5 | 5·1 | 7·3 | 68·492 | 28·098 | 62 | dominated |
| HR-HIVE – Extended | 2·5 | 4·9 | 7·1 | 68·502 | 28·101 | 61 | dominated |
| HIVE – 1 dose | 2·4 | 5·0 | 7·1 | 68·504 | 28·102 | 58 | dominated |
| HIVE – 2 doses | 2·4 | 4·6 | 6·8 | 68·525 | 28·109 | 58 | dominated |
| ALL – 1 dose | 2·2 | 4·7 | 6·7 | 68·524 | 28·109 | 71 | dominated |
| ALL – 2 doses | 2·2 | 4·2 | 6·2 | 68·561 | 28·121 | 85 | dominated |
| HIVE – Extended | 2·4 | 3·5 | 5·7 | 68·581 | 28·127 | 53 | cost-saving*^,^† |
| ALL – 1 dose plus HIVE - Extended | 2·2 | 3·3 | 5·2 | 68·602 | 28·135 | 66 | 1729 |
| ALL - Extended | 2·2 | 2·5 | 4·5 | 68·640 | 28·148 | 126 | 4533 |

*bNAb: broadly neutralizing antibody; IU/IP: intrauterine/intrapartum; yr: year; ICER: incremental cost-effectiveness ratio; YLS: years of life saved; CET: cost-effectiveness threshold; HR-HIVE: high-risk HIV-exposed infants; HIVE: all HIV-exposed infants; ALL: all live infants at birth.*

Pediatric HIV incidence is rounded to the nearest tenth of a percent. IU/IP HIV incidence is calculated based on the number of infants exposed to HIV at birth. Postnatal and total HIV incidence is calculated based on the number of infants ever exposed to HIV through 36 months of life. Undiscounted and discounted life expectancies are rounded to the nearest ten thousandth. Costs are rounded to the nearest dollar and are presented in 2020 USD. Discounted values are discounted at 3% per year. ICERs are rounded to the nearest dollar and are calculated using unrounded discounted life expectancy and discounted costs. The cost-effective bNAb strategy was the strategy that offered the greatest increase in overall population life expectancy while still having an ICER less than the cost-effectiveness threshold when compared to the next best performing, non-dominated strategy. * Indicates the cost-effective strategy using a cost-effectiveness threshold of 20% GDP per capita. † Indicates the cost-effective strategy using a cost-effectiveness threshold of 50% GDP per capita.

# **Supplementary Table 14.** One-way sensitivity analysis: breastfeeding duration

| **Country/strategy** | **Clinical outcomes** | | | **Lifetime efficacy and costs** | | | |
| --- | --- | --- | --- | --- | --- | --- | --- |
|  | **IU/IP cumulative HIV incidence (%)** | **Postnatal cumulative HIV incidence (%)** | **Total cumulative HIV incidence (%)** | **Undiscounted life expectancy (yrs)** | **Discounted life expectancy (yrs)** | **Discounted costs ($)** | **ICER ($/YLS)** |
| **Côte d’Ivoire [CET: ICER ≤ $465/YLS (20% GDP per capita), ICER ≤ $1163/YLS (50% GDP per capita)]** | | | | | | | |
| ***Mean breastfeeding duration: 8 months for all women*** | |  |  |  |  |  |  |
| Standard-of-care | 3·6 | 2·7 | 6·2 | 60·155 | 26·381 | 10 | Reference |
| HR-HIVE – 1 dose | 3·3 | 2·6 | 5·8 | 60·158 | 26·383 | 10 | dominated |
| HR-HIVE – 2 doses | 3·3 | 2·5 | 5·7 | 60·159 | 26·383 | 10 | dominated |
| HIVE – 1 dose | 3·3 | 2·5 | 5·7 | 60·160 | 26·383 | 9 | dominated |
| HR-HIVE – Extended | 3·3 | 2·3 | 5·5 | 60·160 | 26·384 | 10 | dominated |
| HIVE – 2 doses | 3·3 | 2·2 | 5·4 | 60·162 | 26·384 | 9 | dominated |
| ALL – 1 dose | 3·0 | 2·4 | 5·4 | 60·161 | 26·385 | 22 | dominated |
| HIVE – Extended | 3·3 | 1·6 | 4·8 | 60·166 | 26·386 | 9 | cost-saving*^,^† |
| ALL – 2 doses | 3·0 | 2·1 | 5·0 | 60·167 | 26·386 | 33 | dominated |
| ALL – 1 dose plus HIVE - Extended | 3·0 | 1·5 | 4·5 | 60·168 | 26·387 | 21 | 10 463 |
| ALL - Extended | 3·0 | 1·3 | 4·3 | 60·168 | 26·388 | 52 | 51 570 |
| ***Mean breastfeeding duration: 24 months for all women*** | |  |  |  |  |  |  |
| Standard-of-care | 3·6 | 7·9 | 11·2 | 60·121 | 26·369 | 20 | Reference |
| ALL – 1 dose | 3·0 | 7·6 | 10·3 | 60·120 | 26·370 | 34 | dominated |
| HR-HIVE – 1 dose | 3·3 | 7·8 | 10·8 | 60·125 | 26·371 | 20 | dominated |
| HR-HIVE – 2 doses | 3·3 | 7·7 | 10·7 | 60·126 | 26·371 | 20 | dominated |
| HIVE – 1 dose | 3·3 | 7·7 | 10·6 | 60·127 | 26·372 | 19 | dominated |
| ALL – 2 doses | 3·0 | 7·1 | 9·8 | 60·127 | 26·372 | 49 | dominated |
| HR-HIVE – Extended | 3·3 | 7·1 | 10·1 | 60·130 | 26·373 | 20 | dominated |
| HIVE – 2 doses | 3·3 | 7·2 | 10·2 | 60·130 | 26·373 | 19 | dominated |
| ALL – 1 dose plus HIVE - Extended | 3·0 | 5·3 | 8·1 | 60·136 | 26·376 | 31 | dominated |
| HIVE – Extended | 3·3 | 5·4 | 8·4 | 60·142 | 26·378 | 17 | cost-saving*^,^† |
| ALL - Extended | 3·0 | 4·8 | 7·6 | 60·146 | 26·380 | 103 | 40 559 |
| **South Africa [CET: ICER ≤ $1131/YLS (20% GDP per capita), ICER ≤ $2828/YLS (50% GDP per capita)]** | | | | | | | |
| ***Mean breastfeeding duration: 2 months for all women*** | |  |  |  |  |  |  |
| Standard-of-care | 2·3 | 0·9 | 3·2 | 69·125 | 28·566 | 84 | Reference |
| HR-HIVE – 1 dose | 2·1 | 0·9 | 2·9 | 69·178 | 28·583 | 87 | dominated |
| HR-HIVE – 2 doses | 2·1 | 0·8 | 2·9 | 69·181 | 28·585 | 87 | dominated |
| HR-HIVE – Extended | 2·1 | 0·8 | 2·8 | 69·185 | 28·586 | 87 | dominated |
| HIVE – 1 dose | 2·0 | 0·8 | 2·7 | 69·205 | 28·593 | 77 | dominated |
| HIVE – 2 doses | 2·0 | 0·7 | 2·6 | 69·225 | 28·600 | 76 | dominated |
| HIVE – Extended | 2·0 | 0·6 | 2·5 | 69·244 | 28·606 | 75 | cost-saving |
| ALL – 1 dose | 1·8 | 0·7 | 2·4 | 69·252 | 28·610 | 83 | dominated |
| ALL – 2 doses | 1·8 | 0·5 | 2·3 | 69·280 | 28·619 | 85 | dominated |
| ALL – 1 dose plus HIVE - Extended | 1·8 | 0·4 | 2·2 | 69·291 | 28·622 | 81 | 364* |
| ALL - Extended | 1·8 | 0·3 | 2·1 | 69·306 | 28·628 | 87 | 1145† |

**Supplementary Table 14.** One-way sensitivity analysis: breastfeeding duration (cont.)

| **Country/strategy** | **Clinical outcomes** | | | **Lifetime efficacy and costs** | | | |
| --- | --- | --- | --- | --- | --- | --- | --- |
|  | **IU/IP cumulative HIV incidence (%)** | **Postnatal cumulative HIV incidence (%)** | **Total cumulative HIV incidence (%)** | **Undiscounted life expectancy (yrs)** | **Discounted life expectancy (yrs)** | **Discounted costs ($)** | **ICER ($/YLS)** |
| **South Africa [CET: ICER ≤ $1131/YLS (20% GDP per capita), ICER ≤ $2828/YLS (50% GDP per capita)]** | | | | | | | |
| ***Mean breastfeeding duration: 18 months for all women*** | |  |  |  |  |  |  |
| Standard-of-care | 2·3 | 4·9 | 6·9 | 68·494 | 28·361 | 175 | Reference |
| HR-HIVE – 1 dose | 2·1 | 4·7 | 6·6 | 68·553 | 28·381 | 178 | dominated |
| HR-HIVE – 2 doses | 2·1 | 4·7 | 6·5 | 68·563 | 28·385 | 178 | dominated |
| HIVE – 1 dose | 2·0 | 4·6 | 6·3 | 68·591 | 28·394 | 168 | dominated |
| HR-HIVE – Extended | 2·1 | 4·4 | 6·3 | 68·602 | 28·397 | 174 | dominated |
| HIVE – 2 doses | 2·0 | 4·3 | 6·0 | 68·645 | 28·413 | 166 | dominated |
| ALL – 1 dose | 1·8 | 4·4 | 6·0 | 68·645 | 28·413 | 180 | dominated |
| ALL – 2 doses | 1·8 | 3·9 | 5·6 | 68·717 | 28·439 | 186 | dominated |
| HIVE – Extended | 2·0 | 2·9 | 4·7 | 68·860 | 28·482 | 148 | cost-saving |
| ALL – 1 dose plus HIVE - Extended | 1·8 | 2·7 | 4·4 | 68·914 | 28·501 | 159 | 607 |
| ALL - Extended | 1·8 | 2·0 | 3·7 | 69·031 | 28·539 | 192 | 847*^,^† |
| **Zimbabwe [CET: ICER ≤ $243/YLS (20% GDP per capita), ICER ≤ $607/YLS (50% GDP per capita)]** | | | | | | | |
| ***Mean breastfeeding duration: 8 months for women with HIV and 15 months for women without HIV*** | | | |  |  |  |  |
| Standard-of-care | 4·3 | 4·1 | 8·0 | 68·397 | 28·058 | 59 | Reference |
| HR-HIVE – 1 dose | 3·7 | 3·8 | 7·2 | 68·440 | 28·074 | 58 | dominated |
| HR-HIVE – 2 doses | 3·7 | 3·7 | 7·1 | 68·446 | 28·076 | 57 | dominated |
| HIVE – 1 dose | 3·7 | 3·7 | 7·0 | 68·453 | 28·078 | 54 | dominated |
| HR-HIVE – Extended | 3·7 | 3·5 | 7·0 | 68·454 | 28·078 | 57 | dominated |
| HIVE – 2 doses | 3·7 | 3·3 | 6·7 | 68·470 | 28·084 | 53 | dominated |
| ALL – 1 dose | 3·4 | 3·4 | 6·5 | 68·480 | 28·087 | 67 | dominated |
| HIVE – Extended | 3·7 | 2·7 | 6·1 | 68·500 | 28·094 | 51 | cost-saving*^,^† |
| ALL – 2 doses | 3·4 | 2·9 | 6·1 | 68·502 | 28·096 | 80 | dominated |
| ALL – 1 dose plus HIVE - Extended | 3·4 | 2·5 | 5·6 | 68·527 | 28·103 | 64 | 1348 |
| ALL - Extended | 3·4 | 1·8 | 5·0 | 68·554 | 28·113 | 116 | 5362 |
| ***Mean breastfeeding duration: 20 months for all women*** | |  |  |  |  |  |  |
| Standard-of-care | 4·3 | 7·6 | 11·3 | 68·226 | 28·004 | 87 | Reference |
| HR-HIVE – 1 dose | 3·7 | 7·3 | 10·5 | 68·271 | 28·020 | 86 | dominated |
| HR-HIVE – 2 doses | 3·7 | 7·1 | 10·4 | 68·279 | 28·023 | 86 | dominated |
| HIVE – 1 dose | 3·7 | 7·1 | 10·3 | 68·285 | 28·025 | 82 | dominated |
| HR-HIVE – Extended | 3·7 | 6·7 | 9·9 | 68·301 | 28·030 | 84 | dominated |
| ALL – 1 dose | 3·4 | 6·8 | 9·8 | 68·298 | 28·031 | 95 | dominated |
| HIVE – 2 doses | 3·7 | 6·7 | 9·8 | 68·311 | 28·034 | 81 | dominated |
| ALL – 2 doses | 3·4 | 6·3 | 9·2 | 68·340 | 28·044 | 109 | dominated |
| HIVE – Extended | 3·7 | 4·9 | 8·1 | 68·398 | 28·062 | 74 | cost-saving*^,^† |
| ALL – 1 dose plus HIVE - Extended | 3·4 | 4·6 | 7·7 | 68·411 | 28·068 | 86 | 2140 |
| ALL - Extended | 3·4 | 3·8 | 6·8 | 68·467 | 28·086 | 151 | 3624 |

*bNAb: broadly neutralizing antibody; IU/IP: intrauterine/intrapartum; yr: year; ICER: incremental cost-effectiveness ratio; YLS: years of life saved; CET: cost-effectiveness threshold; HR-HIVE: high-risk HIV-exposed infants; HIVE: all HIV-exposed infants; ALL: all live infants at birth.*

Pediatric HIV incidence is rounded to the nearest tenth of a percent. IU/IP HIV incidence is calculated based on the number of infants exposed to HIV at birth. Postnatal and total HIV incidence is calculated based on the number of infants ever exposed to HIV through 36 months of life. Undiscounted and discounted life expectancies are rounded to the nearest ten thousandth. Costs are rounded to the nearest dollar and are presented in 2020 USD. Discounted values are discounted at 3% per year. ICERs are rounded to the nearest dollar and are calculated using unrounded discounted life expectancy and discounted costs. The cost-effective bNAb strategy was the strategy that offered the greatest increase in overall population life expectancy while still having an ICER less than the cost-effectiveness threshold when compared to the next best performing, non-dominated strategy. * Indicates the cost-effective strategy using a cost-effectiveness threshold of 20% GDP per capita. † Indicates the cost-effective strategy using a cost-effectiveness threshold of 50% GDP per capita.

# **Supplementary Table 15.** One-way sensitivity analysis: maternal postpartum HIV incidence

| **Country/strategy** | **Clinical outcomes** | | | **Lifetime efficacy and costs** | | | |
| --- | --- | --- | --- | --- | --- | --- | --- |
|  | **IU/IP cumulative HIV incidence (%)** | **Postnatal cumulative HIV incidence (%)** | **Total cumulative HIV incidence (%)** | **Undiscounted life expectancy (yrs)** | **Discounted life expectancy (yrs)** | **Discounted costs ($)** | **ICER ($/YLS)** |
| **Côte d’Ivoire [CET: ICER ≤ $465/YLS (20% GDP per capita), ICER ≤ $1163/YLS (50% GDP per capita)]** | | | | | | | |
| ***Postpartum incidence: 0·5x base case*** | |  |  |  |  |  |  |
| Standard-of-care | 3·6 | 4·9 | 8·3 | 60·130 | 26·374 | 14 | Reference |
| HR-HIVE – 1 dose | 3·3 | 4·7 | 7·9 | 60·134 | 26·375 | 14 | dominated |
| HR-HIVE – 2 doses | 3·3 | 4·6 | 7·7 | 60·135 | 26·375 | 14 | dominated |
| HIVE – 1 dose | 3·3 | 4·6 | 7·7 | 60·135 | 26·376 | 13 | dominated |
| HR-HIVE – Extended | 3·3 | 4·2 | 7·4 | 60·137 | 26·376 | 13 | dominated |
| HIVE – 2 doses | 3·3 | 4·2 | 7·3 | 60·138 | 26·377 | 13 | dominated |
| ALL – 1 dose | 3·0 | 4·5 | 7·4 | 60·141 | 26·378 | 27 | dominated |
| ALL – 2 doses | 3·0 | 4·0 | 6·9 | 60·141 | 26·378 | 43 | dominated |
| HIVE – Extended | 3·3 | 3·0 | 6·2 | 60·146 | 26·380 | 12 | cost-saving*^,^† |
| ALL – 1 dose plus HIVE - Extended | 3·0 | 3·0 | 5·9 | 60·152 | 26·382 | 26 | 6337 |
| ALL - Extended | 3·0 | 2·6 | 5·5 | 60·156 | 26·383 | 91 | 75 319 |
| ***Postpartum incidence: 2·0x base case*** | |  |  |  |  |  |  |
| Standard-of-care | 3·6 | 5·2 | 8·5 | 60·126 | 26·372 | 15 | Reference |
| HR-HIVE – 1 dose | 3·3 | 5·1 | 8·1 | 60·130 | 26·374 | 15 | dominated |
| HR-HIVE – 2 doses | 3·3 | 5·0 | 8·0 | 60·131 | 26·374 | 15 | dominated |
| HIVE – 1 dose | 3·3 | 5·0 | 8·0 | 60·131 | 26·374 | 14 | dominated |
| HR-HIVE –Extended | 3·3 | 4·6 | 7·7 | 60·133 | 26·375 | 14 | dominated |
| HIVE – 2 doses | 3·3 | 4·6 | 7·6 | 60·135 | 26·376 | 14 | dominated |
| ALL – 1 dose | 3·0 | 4·9 | 7·6 | 60·137 | 26·377 | 28 | dominated |
| ALL – 2 doses | 3·0 | 4·4 | 7·2 | 60·138 | 26·377 | 44 | dominated |
| HIVE – Extended | 3·3 | 3·4 | 6·5 | 60·143 | 26·378 | 13 | cost-saving*^,^† |
| ALL – 1 dose plus HIVE - Extended | 3·0 | 3·3 | 6·2 | 60·149 | 26·381 | 27 | 5993 |
| ALL - Extended | 3·0 | 2·8 | 5·6 | 60·154 | 26·382 | 91 | 58 257 |
| **South Africa [CET: ICER ≤ $1131/YLS (20% GDP per capita), ICER ≤ $2828/YLS (50% GDP per capita)]** | | | | | | | |
| ***Postpartum incidence: 0·5x base case*** | |  |  |  |  |  |  |
| Standard-of-care | 2·3 | 1·9 | 4·1 | 68·984 | 28·519 | 104 | Reference |
| HR-HIVE – 1 dose | 2·1 | 1·8 | 3·7 | 69·039 | 28·538 | 108 | dominated |
| HR-HIVE – 2 doses | 2·1 | 1·7 | 3·7 | 69·046 | 28·541 | 107 | dominated |
| HR-HIVE – Extended | 2·1 | 1·7 | 3·6 | 69·055 | 28·543 | 107 | dominated |
| HIVE – 1 dose | 2·0 | 1·7 | 3·5 | 69·071 | 28·549 | 98 | dominated |
| HIVE – 2 doses | 2·0 | 1·5 | 3·3 | 69·105 | 28·561 | 96 | dominated |
| ALL – 1 dose | 1·8 | 1·5 | 3·2 | 69·116 | 28·565 | 109 | dominated |
| HIVE – Extended | 2·0 | 1·1 | 3·0 | 69·155 | 28·577 | 93 | cost-saving |
| ALL – 2 doses | 1·8 | 1·2 | 2·9 | 69·169 | 28·583 | 116 | dominated |
| ALL – 1 dose plus HIVE - Extended | 1·8 | 1·0 | 2·7 | 69·200 | 28·593 | 104 | 700* |
| ALL - Extended | 1·8 | 0·7 | 2·4 | 69·251 | 28·609 | 131 | 1,675† |

**Supplementary Table 15.** One-way sensitivity analysis: maternal postpartum HIV incidence (cont.)

| **Country/strategy** | **Clinical outcomes** | | | **Lifetime efficacy and costs** | | | |
| --- | --- | --- | --- | --- | --- | --- | --- |
|  | **IU/IP cumulative HIV incidence (%)** | **Postnatal cumulative HIV incidence (%)** | **Total cumulative HIV incidence (%)** | **Undiscounted life expectancy (yrs)** | **Discounted life expectancy (yrs)** | **Discounted costs ($)** | **ICER ($/YLS)** |
| **South Africa [CET: ICER ≤ $1131/YLS (20% GDP per capita), ICER ≤ $2828/YLS (50% GDP per capita)]** | | | | | | | |
| ***Postpartum incidence: 2·0x base case*** | |  |  |  |  |  |  |
| Standard-of-care | 2·3 | 2·8 | 4·8 | 68·813 | 28·462 | 127 | Reference |
| HR-HIVE – 1 dose | 2·1 | 2·7 | 4·5 | 68·868 | 28·481 | 130 | dominated |
| HR-HIVE – 2 doses | 2·1 | 2·6 | 4·5 | 68·875 | 28·483 | 130 | dominated |
| HR-HIVE – Extended | 2·1 | 2·6 | 4·4 | 68·884 | 28·486 | 129 | dominated |
| HIVE – 1 dose | 2·0 | 2·6 | 4·3 | 68·900 | 28·492 | 121 | dominated |
| HIVE – 2 doses | 2·0 | 2·4 | 4·1 | 68·934 | 28·504 | 119 | dominated |
| ALL – 1 dose | 1·8 | 2·3 | 3·9 | 68·960 | 28·513 | 130 | dominated |
| HIVE – Extended | 2·0 | 2·1 | 3·8 | 68·984 | 28·520 | 116 | cost-saving |
| ALL – 2 doses | 1·8 | 1·9 | 3·5 | 69·034 | 28·540 | 135 | dominated |
| ALL – 1 dose plus HIVE - Extended | 1·8 | 1·8 | 3·5 | 69·043 | 28·541 | 125 | dominated |
| ALL - Extended | 1·8 | 1·0 | 2·6 | 69·183 | 28·588 | 140 | 359*^,^† |
| **Zimbabwe [CET: ICER ≤ $243/YLS (20% GDP per capita), ICER ≤ $607/YLS (50% GDP per capita)]** | | | | | | | |
| ***Postpartum incidence: 0·5x base case*** | | |  |  |  |  |  |
| Standard-of-care | 4·3 | 5·2 | 9·2 | 68·351 | 28·044 | 66 | Reference |
| HR-HIVE – 1 dose | 3·7 | 4·9 | 8·3 | 68·396 | 28·060 | 65 | dominated |
| HR-HIVE – 2 doses | 3·7 | 4·7 | 8·2 | 68·403 | 28·062 | 65 | dominated |
| HIVE – 1 dose | 3·7 | 4·7 | 8·1 | 68·410 | 28·065 | 61 | dominated |
| HR-HIVE – Extended | 3·7 | 4·4 | 7·9 | 68·417 | 28·067 | 64 | dominated |
| ALL – 1 dose | 3·4 | 4·4 | 7·6 | 68·430 | 28·072 | 75 | dominated |
| HIVE – 2 doses | 3·7 | 4·3 | 7·7 | 68·432 | 28·073 | 61 | dominated |
| ALL – 2 doses | 3·4 | 3·9 | 7·1 | 68·466 | 28·083 | 89 | dominated |
| HIVE – Extended | 3·7 | 3·1 | 6·6 | 68·488 | 28·090 | 56 | cost-saving*^,^† |
| ALL – 1 dose plus HIVE - Extended | 3·4 | 2·9 | 6·1 | 68·508 | 28·097 | 70 | 1976 |
| ALL - Extended | 3·4 | 2·3 | 5·6 | 68·534 | 28·107 | 131 | 6572 |
| ***Postpartum incidence: 2·0x base case*** | |  |  |  |  |  |  |
| Standard-of-care | 4·3 | 6·4 | 10·0 | 68·258 | 28·014 | 68 | Reference |
| HR-HIVE – 1 dose | 3·7 | 6·1 | 9·2 | 68·303 | 28·030 | 68 | dominated |
| HR-HIVE – 2 doses | 3·7 | 6·0 | 9·1 | 68·310 | 28·032 | 68 | dominated |
| HIVE – 1 dose | 3·7 | 6·0 | 9·0 | 68·317 | 28·035 | 68 | dominated |
| HR-HIVE – Extended | 3·7 | 5·7 | 8·8 | 68·325 | 28·037 | 68 | dominated |
| HIVE – 2 doses | 3·7 | 5·6 | 8·6 | 68·339 | 28·042 | 68 | dominated |
| ALL – 1 dose | 3·4 | 5·7 | 8·5 | 68·342 | 28·043 | 68 | dominated |
| ALL – 2 doses | 3·4 | 5·0 | 7·9 | 68·387 | 28·058 | 68 | dominated |
| HIVE – Extended | 3·7 | 4·5 | 7·6 | 68·395 | 28·060 | 68 | cost-saving*^,^† |
| ALL – 1 dose plus HIVE - Extended | 3·4 | 4·2 | 7·1 | 68·420 | 28·069 | 68 | 1503 |
| ALL - Extended | 3·4 | 3·0 | 5·9 | 68·486 | 28·092 | 68 | 2414 |

*bNAb: broadly neutralizing antibody; IU/IP: intrauterine/intrapartum; yr: year; ICER: incremental cost-effectiveness ratio; YLS: years of life saved; CET: cost-effectiveness threshold; HR-HIVE: high-risk HIV-exposed infants; HIVE: all HIV-exposed infants; ALL: all live infants at birth.*

Pediatric HIV incidence is rounded to the nearest tenth of a percent. IU/IP HIV incidence is calculated based on the number of infants exposed to HIV at birth. Postnatal and total HIV incidence is calculated based on the number of infants ever exposed to HIV through 36 months of life. Undiscounted and discounted life expectancies are rounded to the nearest ten thousandth. Costs are rounded to the nearest dollar and are presented in 2020 USD. Discounted values are discounted at 3% per year. ICERs are rounded to the nearest dollar and are calculated using unrounded discounted life expectancy and discounted costs. The cost-effective bNAb strategy was the strategy that offered the greatest increase in overall population life expectancy while still having an ICER less than the cost-effectiveness threshold when compared to the next best performing, non-dominated strategy. * Indicates the cost-effective strategy using a cost-effectiveness threshold of 20% GDP per capita. † Indicates the cost-effective strategy using a cost-effectiveness threshold of 50% GDP per capita.

# **Supplementary Table 16.** One-way sensitivity analysis: antiretroviral therapy treatment cost

| **Country/strategy** | **Clinical outcomes** | | | **Lifetime efficacy and costs** | | | |
| --- | --- | --- | --- | --- | --- | --- | --- |
|  | **IU/IP cumulative HIV incidence (%)** | **Postnatal cumulative HIV incidence (%)** | **Total cumulative HIV incidence (%)** | **Undiscounted life expectancy (yrs)** | **Discounted life expectancy (yrs)** | **Discounted costs ($)** | **ICER ($/YLS)** |
| **Côte d’Ivoire [CET: ICER ≤ $465/YLS (20% GDP per capita), ICER ≤ $1163/YLS (50% GDP per capita)]** | | | | | | | |
| ***ART treatment cost: 0·5x base case*** | |  |  |  |  |  |  |
| Standard-of-care | 3·6 | 5·0 | 8·4 | 60·129 | 26·373 | 12 | Reference |
| HR-HIVE – 1 dose | 3·3 | 4·8 | 8·0 | 60·133 | 26·375 | 12 | dominated |
| HR-HIVE – 2 doses | 3·3 | 4·7 | 7·8 | 60·134 | 26·375 | 12 | dominated |
| HIVE – 1 dose | 3·3 | 4·7 | 7·8 | 60·134 | 26·375 | 11 | dominated |
| HR-HIVE – Extended | 3·3 | 4·3 | 7·5 | 60·136 | 26·376 | 12 | dominated |
| HIVE – 2 doses | 3·3 | 4·3 | 7·4 | 60·138 | 26·377 | 11 | dominated |
| ALL – 1 dose | 3·0 | 4·6 | 7·5 | 60·139 | 26·377 | 26 | dominated |
| ALL – 2 doses | 3·0 | 4·2 | 7·0 | 60·140 | 26·378 | 41 | dominated |
| HIVE – Extended | 3·3 | 3·2 | 6·3 | 60·146 | 26·379 | 10 | cost-saving*^,^† |
| ALL – 1 dose plus HIVE - Extended | 3·0 | 3·1 | 6·0 | 60·150 | 26·382 | 25 | 6722 |
| ALL - Extended | 3·0 | 2·7 | 5·5 | 60·156 | 26·383 | 89 | 57 928 |
| ***ART treatment cost: 2·0x base case*** | |  |  |  |  |  |  |
| Standard-of-care | 3·6 | 5·0 | 8·4 | 60·129 | 26·373 | 18 | Reference |
| HR-HIVE – 1 dose | 3·3 | 4·8 | 8·0 | 60·133 | 26·375 | 19 | dominated |
| HR-HIVE – 2 doses | 3·3 | 4·7 | 7·8 | 60·134 | 26·375 | 18 | dominated |
| HIVE – 1 dose | 3·3 | 4·7 | 7·8 | 60·134 | 26·375 | 18 | dominated |
| HR-HIVE – Extended | 3·3 | 4·3 | 7·5 | 60·136 | 26·376 | 18 | dominated |
| HIVE – 2 doses | 3·3 | 4·3 | 7·4 | 60·138 | 26·377 | 17 | dominated |
| ALL – 1 dose | 3·0 | 4·6 | 7·5 | 60·139 | 26·377 | 31 | dominated |
| ALL – 2 doses | 3·0 | 4·2 | 7·0 | 60·140 | 26·378 | 47 | dominated |
| HIVE – Extended | 3·3 | 3·2 | 6·3 | 60·146 | 26·379 | 15 | cost-saving*^,^† |
| ALL – 1 dose plus HIVE - Extended | 3·0 | 3·1 | 6·0 | 60·150 | 26·382 | 29 | 6197 |
| ALL - Extended | 3·0 | 2·7 | 5·5 | 60·156 | 26·383 | 93 | 57 720 |
| **South Africa [CET: ICER ≤ $1131/YLS (20% GDP per capita), ICER ≤ $2828/YLS (50% GDP per capita)]** | | | | | | | |
| ***ART treatment cost: 0·5x base case*** | |  |  |  |  |  |  |
| Standard-of-care | 2·3 | 2·2 | 4·4 | 68·924 | 28·499 | 103 | Reference |
| HR-HIVE – 1 dose | 2·1 | 2·1 | 4·0 | 68·979 | 28·518 | 108 | dominated |
| HR-HIVE – 2 doses | 2·1 | 2·1 | 4·0 | 68·986 | 28·521 | 107 | dominated |
| HR-HIVE – Extended | 2·1 | 2·0 | 3·9 | 68·995 | 28·523 | 107 | dominated |
| HIVE – 1 dose | 2·0 | 2·0 | 3·8 | 69·011 | 28·529 | 98 | dominated |
| HIVE – 2 doses | 2·0 | 1·8 | 3·6 | 69·045 | 28·541 | 97 | dominated |
| ALL – 1 dose | 1·8 | 1·8 | 3·5 | 69·063 | 28·548 | 109 | dominated |
| HIVE – Extended | 2·0 | 1·5 | 3·3 | 69·095 | 28·557 | 94 | cost-saving |
| ALL – 2 doses | 1·8 | 1·5 | 3·2 | 69·122 | 28·568 | 116 | dominated |
| ALL – 1 dose plus HIVE - Extended | 1·8 | 1·3 | 3·0 | 69·147 | 28·575 | 105 | 596 |
| ALL - Extended | 1·8 | 0·8 | 2·5 | 69·228 | 28·602 | 129 | 913*^,^† |

**Supplementary Table 16.** One-way sensitivity analysis: antiretroviral therapy treatment cost (cont.)

| **Country/strategy** | **Clinical outcomes** | | | **Lifetime efficacy and costs** | | | |
| --- | --- | --- | --- | --- | --- | --- | --- |
|  | **IU/IP cumulative HIV incidence (%)** | **Postnatal cumulative HIV incidence (%)** | **Total cumulative HIV incidence (%)** | **Undiscounted life expectancy (yrs)** | **Discounted life expectancy (yrs)** | **Discounted costs ($)** | **ICER ($/YLS)** |
| **South Africa [CET: ICER ≤ $1131/YLS (20% GDP per capita), ICER ≤ $2828/YLS (50% GDP per capita)]** | | | | | | | |
| ***ART treatment cost: 2·0x base case*** | |  |  |  |  |  |  |
| Standard-of-care | 2·3 | 2·2 | 4·4 | 68·924 | 28·499 | 130 | Reference |
| HR-HIVE – 1 dose | 2·1 | 2·1 | 4·0 | 68·979 | 28·518 | 131 | dominated |
| HR-HIVE – 2 doses | 2·1 | 2·1 | 4·0 | 68·986 | 28·521 | 131 | dominated |
| HR-HIVE – Extended | 2·1 | 2·0 | 3·9 | 68·995 | 28·523 | 130 | dominated |
| HIVE – 1 dose | 2·0 | 2·0 | 3·8 | 69·011 | 28·529 | 120 | dominated |
| HIVE – 2 doses | 2·0 | 1·8 | 3·6 | 69·045 | 28·541 | 119 | dominated |
| ALL – 1 dose | 1·8 | 1·8 | 3·5 | 69·063 | 28·548 | 130 | dominated |
| HIVE – Extended | 2·0 | 1·5 | 3·3 | 69·095 | 28·557 | 114 | cost-saving |
| ALL – 2 doses | 1·8 | 1·5 | 3·2 | 69·122 | 28·568 | 135 | dominated |
| ALL – 1 dose plus HIVE - Extended | 1·8 | 1·3 | 3·0 | 69·147 | 28·575 | 124 | 518 |
| ALL - Extended | 1·8 | 0·8 | 2·5 | 69·228 | 28·602 | 145 | 819*^,^† |
| **Zimbabwe [CET: ICER ≤ $243/YLS (20% GDP per capita), ICER ≤ $607/YLS (50% GDP per capita)]** | | | | | | | |
| ***ART treatment cost: 0·5x base case*** | | |  |  |  |  |  |
| Standard-of-care | 4·3 | 5·6 | 9·5 | 68·321 | 28·034 | 62 | Reference |
| HR-HIVE – 1 dose | 3·7 | 5·3 | 8·6 | 68·366 | 28·050 | 61 | dominated |
| HR-HIVE – 2 doses | 3·7 | 5·2 | 8·5 | 68·373 | 28·053 | 61 | dominated |
| HIVE – 1 dose | 3·7 | 5·2 | 8·4 | 68·380 | 28·055 | 58 | dominated |
| HR-HIVE – Extended | 3·7 | 4·9 | 8·2 | 68·387 | 28·057 | 60 | dominated |
| ALL – 1 dose | 3·4 | 4·9 | 7·9 | 68·400 | 28·062 | 71 | dominated |
| HIVE – 2 doses | 3·7 | 4·8 | 8·0 | 68·402 | 28·063 | 57 | dominated |
| ALL – 2 doses | 3·4 | 4·3 | 7·4 | 68·438 | 28·074 | 85 | dominated |
| HIVE – Extended | 3·7 | 3·6 | 6·9 | 68·458 | 28·081 | 54 | cost-saving*^,^† |
| ALL – 1 dose plus HIVE - Extended | 3·4 | 3·4 | 6·5 | 68·478 | 28·088 | 67 | 1777 |
| ALL - Extended | 3·4 | 2·6 | 5·7 | 68·517 | 28·101 | 128 | 4601 |
| ***ART treatment cost: 2·0x base case*** | |  |  |  |  |  |  |
| Standard-of-care | 4·3 | 5·6 | 9·5 | 68·321 | 28·034 | 91 | Reference |
| HR-HIVE – 1 dose | 3·7 | 5·3 | 8·6 | 68·366 | 28·050 | 88 | dominated |
| HR-HIVE – 2 doses | 3·7 | 5·2 | 8·5 | 68·373 | 28·053 | 87 | dominated |
| HIVE – 1 dose | 3·7 | 5·2 | 8·4 | 68·380 | 28·055 | 84 | dominated |
| HR-HIVE – Extended | 3·7 | 4·9 | 8·2 | 68·387 | 28·057 | 86 | dominated |
| ALL – 1 dose | 3·4 | 4·9 | 7·9 | 68·400 | 28·062 | 95 | dominated |
| HIVE – 2 doses | 3·7 | 4·8 | 8·0 | 68·402 | 28·063 | 82 | dominated |
| ALL – 2 doses | 3·4 | 4·3 | 7·4 | 68·438 | 28·074 | 108 | dominated |
| HIVE – Extended | 3·7 | 3·6 | 6·9 | 68·458 | 28·081 | 75 | cost-saving*^,^† |
| ALL – 1 dose plus HIVE - Extended | 3·4 | 3·4 | 6·5 | 68·478 | 28·088 | 86 | 1522 |
| ALL - Extended | 3·4 | 2·6 | 5·7 | 68·517 | 28·101 | 145 | 4411 |

*bNAb: broadly neutralizing antibody; IU/IP: intrauterine/intrapartum; yr: year; ICER: incremental cost-effectiveness ratio; YLS: years of life saved; CET: cost-effectiveness threshold; HR-HIVE: high-risk HIV-exposed infants; HIVE: all HIV-exposed infants; ALL: all live infants at birth.*

Pediatric HIV incidence is rounded to the nearest tenth of a percent. IU/IP HIV incidence is calculated based on the number of infants exposed to HIV at birth. Postnatal and total HIV incidence is calculated based on the number of infants ever exposed to HIV through 36 months of life. Undiscounted and discounted life expectancies are rounded to the nearest ten thousandth. Costs are rounded to the nearest dollar and are presented in 2020 USD. Discounted values are discounted at 3% per year. ICERs are rounded to the nearest dollar and are calculated using unrounded discounted life expectancy and discounted costs. The cost-effective bNAb strategy was the strategy that offered the greatest increase in overall population life expectancy while still having an ICER less than the cost-effectiveness threshold when compared to the next best performing, non-dominated strategy. * Indicates the cost-effective strategy using a cost-effectiveness threshold of 20% GDP per capita. † Indicates the cost-effective strategy using a cost-effectiveness threshold of 50% GDP per capita.

# **Supplementary Table 17.** One-way sensitivity analysis: postpartum vertical transmission risk

| **Country/strategy** | **Clinical outcomes** | | | **Lifetime efficacy and costs** | | | |
| --- | --- | --- | --- | --- | --- | --- | --- |
|  | **IU/IP cumulative HIV incidence (%)** | **Postnatal cumulative HIV incidence (%)** | **Total cumulative HIV incidence (%)** | **Undiscounted life expectancy (yrs)** | **Discounted life expectancy (yrs)** | **Discounted costs ($)** | **ICER ($/YLS)** |
| **Côte d’Ivoire [CET: ICER ≤ $465/YLS (20% GDP per capita), ICER ≤ $1163/YLS (50% GDP per capita)]** | | | | | | | |
| ***Postpartum vertical transmission risk: 0·5x base case*** | |  |  |  |  |  |  |
| Standard-of-care | 3·6 | 2·5 | 6·0 | 60·147 | 26·380 | 10 | Reference |
| HR-HIVE – 1 dose | 3·3 | 2·5 | 5·7 | 60·150 | 26·381 | 10 | dominated |
| HR-HIVE – 2 doses | 3·3 | 2·4 | 5·6 | 60·150 | 26·381 | 10 | dominated |
| HIVE – 1 dose | 3·3 | 2·4 | 5·5 | 60·151 | 26·382 | 9 | dominated |
| HR-HIVE – Extended | 3·3 | 2·2 | 5·4 | 60·152 | 26·382 | 10 | dominated |
| HIVE – 2 doses | 3·3 | 2·2 | 5·3 | 60·153 | 26·382 | 9 | dominated |
| ALL – 2 doses | 3·0 | 2·1 | 5·0 | 60·155 | 26·383 | 39 | dominated |
| ALL – 1 dose | 3·0 | 2·4 | 5·3 | 60·156 | 26·384 | 24 | dominated |
| HIVE – Extended | 3·3 | 1·6 | 4·8 | 60·157 | 26·384 | 9 | cost-saving*^,^† |
| ALL – 1 dose plus HIVE - Extended | 3·0 | 1·6 | 4·5 | 60·161 | 26·386 | 24 | 7038 |
| ALL - Extended | 3·0 | 1·3 | 4·3 | 60·166 | 26·386 | 89 | 112 676 |
| ***Postpartum vertical transmission risk: 2·0x base case*** | |  |  |  |  |  |  |
| Standard-of-care | 3·6 | 9·6 | 12·8 | 60·096 | 26·361 | 22 | Reference |
| HR-HIVE – 1 dose | 3·3 | 9·3 | 12·3 | 60·101 | 26·362 | 22 | dominated |
| HR-HIVE – 2 doses | 3·3 | 9·1 | 12·0 | 60·103 | 26·363 | 22 | dominated |
| HIVE – 1 dose | 3·3 | 9·1 | 12·0 | 60·103 | 26·364 | 21 | dominated |
| HR-HIVE – Extended | 3·3 | 8·4 | 11·3 | 60·107 | 26·365 | 21 | dominated |
| ALL – 1 dose | 3·0 | 8·9 | 11·6 | 60·109 | 26·366 | 35 | dominated |
| HIVE – 2 doses | 3·3 | 8·3 | 11·2 | 60·110 | 26·366 | 20 | dominated |
| ALL – 2 doses | 3·0 | 8·0 | 10·7 | 60·113 | 26·368 | 50 | dominated |
| HIVE – Extended | 3·3 | 6·1 | 9·1 | 60·125 | 26·372 | 17 | cost-saving*^,^† |
| ALL – 1 dose plus HIVE - Extended | 3·0 | 5·9 | 8·7 | 60·130 | 26·374 | 31 | 6027 |
| ALL - Extended | 3·0 | 5·2 | 8·0 | 60·138 | 26·376 | 95 | 32 149 |
| **South Africa [CET: ICER ≤ $1131/YLS (20% GDP per capita), ICER ≤ $2828/YLS (50% GDP per capita)]** | | | | | | | |
| ***Postpartum vertical transmission risk: 0·5x base case*** | |  |  |  |  |  |  |
| Standard-of-care | 2·3 | 1·1 | 3·3 | 69·097 | 28·558 | 90 | Reference |
| HR-HIVE – 1 dose | 2·1 | 1·1 | 3·0 | 69·146 | 28·574 | 94 | dominated |
| HR-HIVE – 2 doses | 2·1 | 1·1 | 3·0 | 69·149 | 28·576 | 94 | dominated |
| HR-HIVE – Extended | 2·1 | 1·0 | 3·0 | 69·154 | 28·577 | 94 | dominated |
| HIVE – 1 dose | 2·0 | 1·0 | 2·9 | 69·169 | 28·582 | 85 | cost-saving |
| HIVE – 2 doses | 2·0 | 0·9 | 2·8 | 69·187 | 28·589 | 85 | 103 |
| ALL – 1 dose | 1·8 | 0·9 | 2·6 | 69·205 | 28·595 | 97 | dominated |
| HIVE – Extended | 2·0 | 0·8 | 2·6 | 69·212 | 28·597 | 85 | dominated |
| ALL – 2 doses | 1·8 | 0·7 | 2·5 | 69·237 | 28·606 | 107 | dominated |
| ALL – 1 dose plus HIVE - Extended | 1·8 | 0·7 | 2·4 | 69·248 | 28·609 | 98 | 590* |
| ALL - Extended | 1·8 | 0·4 | 2·1 | 69·291 | 28·623 | 126 | 2023† |

**Supplementary Table 17.** One-way sensitivity analysis: postpartum vertical transmission risk (cont.)

| **Country/strategy** | **Clinical outcomes** | | | **Lifetime efficacy and costs** | | | |
| --- | --- | --- | --- | --- | --- | --- | --- |
|  | **IU/IP cumulative HIV incidence (%)** | **Postnatal cumulative HIV incidence (%)** | **Total cumulative HIV incidence (%)** | **Undiscounted life expectancy (yrs)** | **Discounted life expectancy (yrs)** | **Discounted costs ($)** | **ICER ($/YLS)** |
| **South Africa [CET: ICER ≤ $1131/YLS (20% GDP per capita), ICER ≤ $2828/YLS (50% GDP per capita)]** | | | | | | | |
| ***Postpartum vertical transmission risk: 2·0x base case*** | |  |  |  |  |  |  |
| Standard-of-care | 2·3 | 4·2 | 6·3 | 68·599 | 28·390 | 154 | Reference |
| HR-HIVE – 1 dose | 2·1 | 4·0 | 5·9 | 68·668 | 28·413 | 156 | dominated |
| HR-HIVE – 2 doses | 2·1 | 3·9 | 5·8 | 68·681 | 28·418 | 155 | dominated |
| HR-HIVE – Extended | 2·1 | 3·8 | 5·7 | 68·698 | 28·423 | 153 | dominated |
| HIVE – 1 dose | 2·0 | 3·8 | 5·6 | 68·719 | 28·431 | 143 | dominated |
| HIVE – 2 doses | 2·0 | 3·4 | 5·2 | 68·784 | 28·453 | 139 | dominated |
| ALL – 1 dose | 1·8 | 3·4 | 5·1 | 68·797 | 28·459 | 151 | dominated |
| HIVE – Extended | 2·0 | 2·8 | 4·6 | 68·880 | 28·484 | 129 | cost-saving |
| ALL – 2 doses | 1·8 | 2·8 | 4·5 | 68·904 | 28·496 | 151 | dominated |
| ALL – 1 dose plus HIVE - Extended | 1·8 | 2·4 | 4·1 | 68·958 | 28·512 | 136 | 260 |
| ALL - Extended | 1·8 | 1·6 | 3·3 | 69·103 | 28·560 | 151 | 315*^,^† |
| **Zimbabwe [CET: ICER ≤ $243/YLS (20% GDP per capita), ICER ≤ $607/YLS (50% GDP per capita)]** | | | | | | | |
| ***Postpartum vertical transmission risk: 0·5x base case*** | | |  |  |  |  |  |
| Standard-of-care | 4·3 | 2·9 | 6·8 | 68·461 | 28·081 | 52 | Reference |
| HR-HIVE – 1 dose | 3·7 | 2·7 | 6·1 | 68·499 | 28·094 | 52 | dominated |
| HR-HIVE – 2 doses | 3·7 | 2·7 | 6·1 | 68·502 | 28·095 | 52 | dominated |
| HIVE – 1 dose | 3·7 | 2·6 | 6·0 | 68·508 | 28·097 | 48 | cost-saving |
| HR-HIVE – Extended | 3·7 | 2·5 | 5·9 | 68·510 | 28·098 | 52 | dominated |
| HIVE – 2 doses | 3·7 | 2·4 | 5·8 | 68·519 | 28·101 | 49 | dominated |
| ALL – 1 dose | 3·4 | 2·5 | 5·6 | 68·522 | 28·102 | 61 | dominated |
| ALL – 2 doses | 3·4 | 2·2 | 5·3 | 68·546 | 28·109 | 77 | dominated |
| HIVE – Extended | 3·7 | 1·9 | 5·2 | 68·548 | 28·110 | 49 | 18*^,^† |
| ALL – 1 dose plus HIVE - Extended | 3·4 | 1·7 | 4·9 | 68·562 | 28·115 | 62 | 2656 |
| ALL - Extended | 3·4 | 1·3 | 4·5 | 68·582 | 28·123 | 124 | 8712 |
| ***Postpartum vertical transmission risk: 2·0x base case*** | |  |  |  |  |  |  |
| Standard-of-care | 4·3 | 10·7 | 14·3 | 68·060 | 27·946 | 107 | Reference |
| HR-HIVE – 1 dose | 3·7 | 10·1 | 13·2 | 68·118 | 27·967 | 105 | dominated |
| HR-HIVE – 2 doses | 3·7 | 9·9 | 13·0 | 68·132 | 27·972 | 104 | dominated |
| HIVE – 1 dose | 3·7 | 9·8 | 12·9 | 68·141 | 27·976 | 100 | dominated |
| HR-HIVE – Extended | 3·7 | 9·3 | 12·5 | 68·159 | 27·981 | 100 | dominated |
| ALL – 1 dose | 3·4 | 9·3 | 12·2 | 68·173 | 27·987 | 112 | dominated |
| HIVE – 2 doses | 3·7 | 9·0 | 12·1 | 68·185 | 27·991 | 97 | dominated |
| ALL – 2 doses | 3·4 | 8·3 | 11·2 | 68·239 | 28·009 | 122 | dominated |
| HIVE – Extended | 3·7 | 6·8 | 10·0 | 68·292 | 28·025 | 84 | cost-saving*^,^† |
| ALL – 1 dose plus HIVE - Extended | 3·4 | 6·4 | 9·4 | 68·323 | 28·036 | 96 | 1061 |
| ALL - Extended | 3·4 | 5·0 | 8·0 | 68·393 | 28·060 | 152 | 2370 |

*bNAb: broadly neutralizing antibody; IU/IP: intrauterine/intrapartum; yr: year; ICER: incremental cost-effectiveness ratio; YLS: years of life saved; CET: cost-effectiveness threshold; HR-HIVE: high-risk HIV-exposed infants; HIVE: all HIV-exposed infants; ALL: all live infants at birth.*

Pediatric HIV incidence is rounded to the nearest tenth of a percent. IU/IP HIV incidence is calculated based on the number of infants exposed to HIV at birth. Postnatal and total HIV incidence is calculated based on the number of infants ever exposed to HIV through 36 months of life. Undiscounted and discounted life expectancies are rounded to the nearest ten thousandth. Costs are rounded to the nearest dollar and are presented in 2020 USD. Discounted values are discounted at 3% per year. ICERs are rounded to the nearest dollar and are calculated using unrounded discounted life expectancy and discounted costs. The cost-effective bNAb strategy was the strategy that offered the greatest increase in overall population life expectancy while still having an ICER less than the cost-effectiveness threshold when compared to the next best performing, non-dominated strategy. * Indicates the cost-effective strategy using a cost-effectiveness threshold of 20% GDP per capita. † Indicates the cost-effective strategy using a cost-effectiveness threshold of 50% GDP per capita.

# **Supplementary Table 18.** One-way sensitivity analysis: infant oral prophylaxis efficacy (base case: 69% intrapartum efficacy and 71% postpartum efficacy)

| **Country/strategy** | **Clinical outcomes** | | | **Lifetime efficacy and costs** | | | |
| --- | --- | --- | --- | --- | --- | --- | --- |
|  | **IU/IP cumulative HIV incidence (%)** | **Postnatal cumulative HIV incidence (%)** | **Total cumulative HIV incidence (%)** | **Undiscounted life expectancy (yrs)** | **Discounted life expectancy (yrs)** | **Discounted costs ($)** | **ICER ($/YLS)** |
| **Côte d’Ivoire [CET: ICER ≤ $465/YLS (20% GDP per capita), ICER ≤ $1163/YLS (50% GDP per capita)]** | | | | | | | |
| ***Low infant oral prophylaxis efficacy* *(intrapartum efficacy: 40%; postpartum efficacy: 58%)*** | | | | | | | |
| Standard-of-care | 3·7 | 5·1 | 8·5 | 60·128 | 26·373 | 15 | Reference |
| HR-HIVE – 1 dose | 3·4 | 4·9 | 8·1 | 60·132 | 26·374 | 15 | dominated |
| HR-HIVE – 2 doses | 3·4 | 4·8 | 7·9 | 60·133 | 26·375 | 15 | dominated |
| HIVE – 1 dose | 3·3 | 4·8 | 7·9 | 60·134 | 26·375 | 14 | dominated |
| HR-HIVE – Extended | 3·4 | 4·4 | 7·6 | 60·135 | 26·376 | 15 | dominated |
| HIVE – 2 doses | 3·3 | 4·4 | 7·5 | 60·137 | 26·376 | 14 | dominated |
| ALL – 1 dose | 3·1 | 4·7 | 7·6 | 60·139 | 26·377 | 28 | dominated |
| ALL – 2 doses | 3·1 | 4·2 | 7·1 | 60·139 | 26·378 | 43 | dominated |
| HIVE – Extended | 3·3 | 3·2 | 6·4 | 60·145 | 26·379 | 13 | cost-saving*^,^† |
| ALL – 1 dose plus HIVE - Extended | 3·1 | 3·1 | 6·1 | 60·150 | 26·381 | 26 | 6240 |
| ALL - Extended | 3·1 | 2·7 | 5·6 | 60·155 | 26·382 | 91 | 57 858 |
| ***High infant oral prophylaxis efficacy (intrapartum efficacy: 90%; postpartum efficacy: 80%)*** | | | | | | | |
| Standard-of-care | 3·6 | 4·9 | 8·3 | 60·130 | 26·374 | 14 | Reference |
| HR-HIVE – 1 dose | 3·3 | 4·8 | 7·9 | 60·133 | 26·375 | 15 | dominated |
| HR-HIVE – 2 doses | 3·3 | 4·7 | 7·7 | 60·135 | 26·375 | 15 | dominated |
| HIVE – 1 dose | 3·2 | 4·7 | 7·7 | 60·135 | 26·376 | 14 | dominated |
| HR-HIVE – Extended | 3·3 | 4·3 | 7·4 | 60·137 | 26·376 | 14 | dominated |
| HIVE – 2 doses | 3·2 | 4·3 | 7·3 | 60·138 | 26·377 | 14 | dominated |
| ALL – 1 dose | 3·0 | 4·6 | 7·4 | 60·140 | 26·378 | 27 | dominated |
| ALL – 2 doses | 3·0 | 4·1 | 6·9 | 60·141 | 26·378 | 43 | dominated |
| HIVE – Extended | 3·2 | 3·1 | 6·2 | 60·146 | 26·380 | 13 | cost-saving*^,^† |
| ALL – 1 dose plus HIVE - Extended | 3·0 | 3·1 | 5·9 | 60·151 | 26·382 | 26 | 6246 |
| ALL - Extended | 3·0 | 2·6 | 5·5 | 60·157 | 26·383 | 91 | 57 856 |
| **South Africa [CET: ICER ≤ $1131/YLS (20% GDP per capita), ICER ≤ $2828/YLS (50% GDP per capita)]** | | | | | | | |
| ***Low infant oral prophylaxis efficacy* *(intrapartum efficacy: 40%; postpartum efficacy: 58%)*** | | | | | | | |
| Standard-of-care | 2·4 | 2·2 | 4·5 | 68·909 | 28·494 | 114 | Reference |
| HR-HIVE – 1 dose | 2·1 | 2·1 | 4·1 | 68·970 | 28·515 | 116 | dominated |
| HR-HIVE – 2 doses | 2·1 | 2·1 | 4·0 | 68·977 | 28·517 | 116 | dominated |
| HR-HIVE – Extended | 2·1 | 2·0 | 4·0 | 68·985 | 28·520 | 115 | dominated |
| HIVE – 1 dose | 2·0 | 2·0 | 3·9 | 69·003 | 28·526 | 106 | dominated |
| HIVE – 2 doses | 2·0 | 1·8 | 3·7 | 69·037 | 28·538 | 105 | dominated |
| ALL – 1 dose | 1·8 | 1·8 | 3·5 | 69·055 | 28·545 | 117 | dominated |
| HIVE – Extended | 2·0 | 1·5 | 3·4 | 69·086 | 28·554 | 101 | cost-saving |
| ALL – 2 doses | 1·8 | 1·5 | 3·2 | 69·114 | 28·565 | 123 | dominated |
| ALL – 1 dose plus HIVE - Extended | 1·8 | 1·3 | 3·0 | 69·139 | 28·573 | 112 | 596 |
| ALL - Extended | 1·8 | 0·8 | 2·6 | 69·220 | 28·599 | 136 | 882*^,^† |

**Supplementary Table 18.** One-way sensitivity analysis: infant oral prophylaxis efficacy (base case: 69% intrapartum efficacy and 71% postpartum efficacy) (cont.)

| **Country/strategy** | **Clinical outcomes** | | | **Lifetime efficacy and costs** | | | |
| --- | --- | --- | --- | --- | --- | --- | --- |
|  | **IU/IP cumulative HIV incidence (%)** | **Postnatal cumulative HIV incidence (%)** | **Total cumulative HIV incidence (%)** | **Undiscounted life expectancy (yrs)** | **Discounted life expectancy (yrs)** | **Discounted costs ($)** | **ICER ($/YLS)** |
| **South Africa [CET: ICER ≤ $1131/YLS (20% GDP per capita), ICER ≤ $2828/YLS (50% GDP per capita)]** | | | | | | | |
| ***High infant oral prophylaxis efficacy (intrapartum efficacy: 90%; postpartum efficacy: 80%)*** | | | |  |  |  |  |
| Standard-of-care | 2·3 | 2·2 | 4·3 | 68·934 | 28·503 | 110 | Reference |
| HR-HIVE – 1 dose | 2·0 | 2·1 | 4·0 | 68·986 | 28·521 | 114 | dominated |
| HR-HIVE – 2 doses | 2·0 | 2·0 | 3·9 | 68·993 | 28·523 | 114 | dominated |
| HR-HIVE – Extended | 2·0 | 2·0 | 3·9 | 69·002 | 28·526 | 113 | dominated |
| HIVE – 1 dose | 1·9 | 2·0 | 3·8 | 69·017 | 28·531 | 104 | dominated |
| HIVE – 2 doses | 1·9 | 1·8 | 3·6 | 69·051 | 28·543 | 103 | dominated |
| ALL – 1 dose | 1·8 | 1·8 | 3·4 | 69·069 | 28·550 | 115 | dominated |
| HIVE – Extended | 1·9 | 1·5 | 3·3 | 69·101 | 28·559 | 100 | cost-saving |
| ALL – 2 doses | 1·8 | 1·4 | 3·1 | 69·127 | 28·570 | 122 | dominated |
| ALL – 1 dose plus HIVE - Extended | 1·8 | 1·3 | 2·9 | 69·152 | 28·577 | 111 | 613 |
| ALL - Extended | 1·8 | 0·8 | 2·5 | 69·233 | 28·604 | 134 | 882*^,^† |
| **Zimbabwe [CET: ICER ≤ $243/YLS (20% GDP per capita), ICER ≤ $607/YLS (50% GDP per capita)]** | | | | | | | |
| ***Low infant oral prophylaxis efficacy* *(intrapartum efficacy: 40%; postpartum efficacy: 58%)*** | | | | | | | |
| Standard-of-care | 4·5 | 5·8 | 9·8 | 68·303 | 28·027 | 74 | Reference |
| HR-HIVE – 1 dose | 3·9 | 5·4 | 8·8 | 68·354 | 28·046 | 72 | dominated |
| HR-HIVE – 2 doses | 3·9 | 5·3 | 8·7 | 68·361 | 28·048 | 71 | dominated |
| HIVE – 1 dose | 3·8 | 5·2 | 8·6 | 68·369 | 28·051 | 68 | dominated |
| HR-HIVE – Extended | 3·9 | 5·0 | 8·4 | 68·375 | 28·053 | 70 | dominated |
| ALL – 1 dose | 3·6 | 4·9 | 8·1 | 68·389 | 28·058 | 81 | dominated |
| HIVE – 2 doses | 3·8 | 4·8 | 8·2 | 68·391 | 28·059 | 67 | dominated |
| ALL – 2 doses | 3·6 | 4·4 | 7·6 | 68·427 | 28·070 | 94 | dominated |
| HIVE – Extended | 3·8 | 3·7 | 7·1 | 68·446 | 28·076 | 63 | cost-saving*^,^† |
| ALL – 1 dose plus HIVE - Extended | 3·6 | 3·4 | 6·7 | 68·467 | 28·084 | 75 | 1715 |
| ALL - Extended | 3·6 | 2·6 | 5·9 | 68·506 | 28·097 | 135 | 4520 |
| ***High infant oral prophylaxis efficacy (intrapartum efficacy: 90%; postpartum efficacy: 80%)*** | | | | | | | |
| Standard-of-care | 4·2 | 5·5 | 9·2 | 68·334 | 28·038 | 70 | Reference |
| HR-HIVE – 1 dose | 3·6 | 5·3 | 8·5 | 68·374 | 28·053 | 70 | dominated |
| HR-HIVE – 2 doses | 3·6 | 5·1 | 8·3 | 68·382 | 28·056 | 69 | dominated |
| HIVE – 1 dose | 3·6 | 5·1 | 8·3 | 68·388 | 28·058 | 66 | dominated |
| HR-HIVE – Extended | 3·6 | 4·8 | 8·1 | 68·396 | 28·060 | 68 | dominated |
| ALL – 1 dose | 3·3 | 4·8 | 7·8 | 68·408 | 28·065 | 78 | dominated |
| HIVE – 2 doses | 3·6 | 4·7 | 7·9 | 68·410 | 28·066 | 65 | dominated |
| ALL – 2 doses | 3·3 | 4·3 | 7·3 | 68·446 | 28·077 | 92 | dominated |
| HIVE – Extended | 3·6 | 3·6 | 6·8 | 68·466 | 28·083 | 60 | cost-saving*^,^† |
| ALL – 1 dose plus HIVE - Extended | 3·3 | 3·3 | 6·3 | 68·486 | 28·091 | 73 | 1738 |
| ALL - Extended | 3·3 | 2·5 | 5·5 | 68·525 | 28·104 | 133 | 4516 |

*bNAb: broadly neutralizing antibody; IU/IP: intrauterine/intrapartum; yr: year; ICER: incremental cost-effectiveness ratio; YLS: years of life saved; CET: cost-effectiveness threshold; HR-HIVE: high-risk HIV-exposed infants; HIVE: all HIV-exposed infants; ALL: all live infants at birth.*

Pediatric HIV incidence is rounded to the nearest tenth of a percent. IU/IP HIV incidence is calculated based on the number of infants exposed to HIV at birth. Postnatal and total HIV incidence is calculated based on the number of infants ever exposed to HIV through 36 months of life. Undiscounted and discounted life expectancies are rounded to the nearest ten thousandth. Costs are rounded to the nearest dollar and are presented in 2020 USD. Discounted values are discounted at 3% per year. ICERs are rounded to the nearest dollar and are calculated using unrounded discounted life expectancy and discounted costs. The cost-effective bNAb strategy was the strategy that offered the greatest increase in overall population life expectancy while still having an ICER less than the cost-effectiveness threshold when compared to the next best performing, non-dominated strategy. * Indicates the cost-effective strategy using a cost-effectiveness threshold of 20% GDP per capita. † Indicates the cost-effective strategy using a cost-effectiveness threshold of 50% GDP per capita.

# **Supplementary Table 19.** One-way sensitivity analysis: additional cost of identifying high-risk infants

| **Country/strategy** | **Clinical outcomes** | | | **Lifetime efficacy and costs** | | | |
| --- | --- | --- | --- | --- | --- | --- | --- |
|  | **IU/IP cumulative HIV incidence (%)** | **Postnatal cumulative HIV incidence (%)** | **Total cumulative HIV incidence (%)** | **Undiscounted life expectancy (yrs)** | **Discounted life expectancy (yrs)** | **Discounted costs ($)** | **ICER ($/YLS)** |
| **Côte d’Ivoire [CET: ICER ≤ $465/YLS (20% GDP per capita), ICER ≤ $1163/YLS (50% GDP per capita)]** | | | | | | | |
| ***Cost of identifying high-risk infants: $0*** | | |  |  |  |  |  |
| Standard-of-care | 3·6 | 5·0 | 8·4 | 60·129 | 26·373 | 15 | Reference |
| HR-HIVE – 1 dose | 3·3 | 4·8 | 8·0 | 60·133 | 26·375 | 14 | dominated |
| HR-HIVE – 2 doses | 3·3 | 4·7 | 7·8 | 60·134 | 26·375 | 14 | dominated |
| HIVE – 1 dose | 3·3 | 4·7 | 7·8 | 60·134 | 26·375 | 14 | dominated |
| HR-HIVE – Extended | 3·3 | 4·3 | 7·5 | 60·136 | 26·376 | 14 | dominated |
| HIVE – 2 doses | 3·3 | 4·3 | 7·4 | 60·138 | 26·377 | 14 | dominated |
| ALL – 1 dose | 3·0 | 4·6 | 7·5 | 60·139 | 26·377 | 27 | dominated |
| ALL – 2 doses | 3·0 | 4·2 | 7·0 | 60·140 | 26·378 | 43 | dominated |
| HIVE – Extended | 3·3 | 3·2 | 6·3 | 60·146 | 26·379 | 13 | cost-saving*^,^† |
| ALL – 1 dose plus HIVE - Extended | 3·0 | 3·1 | 6·0 | 60·150 | 26·382 | 26 | 6242 |
| ALL - Extended | 3·0 | 2·7 | 5·5 | 60·156 | 26·383 | 91 | 57 860 |
| ***Cost of identifying high-risk infants: $32*** | | |  |  |  |  |  |
| Standard-of-care | 3·6 | 5·0 | 8·4 | 60·129 | 26·373 | 15 | Reference |
| HR-HIVE – 1 dose | 3·3 | 4·8 | 8·0 | 60·133 | 26·375 | 15 | dominated |
| HR-HIVE – 2 doses | 3·3 | 4·7 | 7·8 | 60·134 | 26·375 | 15 | dominated |
| HIVE – 1 dose | 3·3 | 4·7 | 7·8 | 60·134 | 26·375 | 14 | dominated |
| HR-HIVE – Extended | 3·3 | 4·3 | 7·5 | 60·136 | 26·376 | 14 | dominated |
| HIVE – 2 doses | 3·3 | 4·3 | 7·4 | 60·138 | 26·377 | 14 | dominated |
| ALL – 1 dose | 3·0 | 4·6 | 7·5 | 60·139 | 26·377 | 27 | dominated |
| ALL – 2 doses | 3·0 | 4·2 | 7·0 | 60·140 | 26·378 | 43 | dominated |
| HIVE – Extended | 3·3 | 3·2 | 6·3 | 60·146 | 26·379 | 13 | cost-saving*^,^† |
| ALL – 1 dose plus HIVE - Extended | 3·0 | 3·1 | 6·0 | 60·150 | 26·382 | 26 | 6242 |
| ALL - Extended | 3·0 | 2·7 | 5·5 | 60·156 | 26·383 | 91 | 57 860 |
| **South Africa [CET: ICER ≤ $1131/YLS (20% GDP per capita), ICER ≤ $2828/YLS (50% GDP per capita)]** | | | | | | | |
| ***Cost of identifying high-risk infants: $0*** | |  |  |  |  |  |  |
| Standard-of-care | 2·3 | 2·2 | 4·4 | 68·924 | 28·499 | 112 | Reference |
| HR-HIVE – 1 dose | 2·1 | 2·1 | 4·0 | 68·979 | 28·518 | 105 | dominated |
| HR-HIVE – 2 doses | 2·1 | 2·1 | 4·0 | 68·986 | 28·521 | 105 | dominated |
| HR-HIVE – Extended | 2·1 | 2·0 | 3·9 | 68·995 | 28·523 | 104 | dominated |
| HIVE – 1 dose | 2·0 | 2·0 | 3·8 | 69·011 | 28·529 | 105 | dominated |
| HIVE – 2 doses | 2·0 | 1·8 | 3·6 | 69·045 | 28·541 | 104 | dominated |
| ALL – 1 dose | 1·8 | 1·8 | 3·5 | 69·063 | 28·548 | 116 | dominated |
| HIVE – Extended | 2·0 | 1·5 | 3·3 | 69·095 | 28·557 | 100 | cost-saving |
| ALL – 2 doses | 1·8 | 1·5 | 3·2 | 69·122 | 28·568 | 122 | dominated |
| ALL – 1 dose plus HIVE - Extended | 1·8 | 1·3 | 3·0 | 69·147 | 28·575 | 111 | 606 |
| ALL - Extended | 1·8 | 0·8 | 2·5 | 69·228 | 28·602 | 135 | 882*^,^† |

**Supplementary Table 19.** One-way sensitivity analysis: additional cost of identifying high-risk infants (cont.)

| **Country/strategy** | **Clinical outcomes** | | | **Lifetime efficacy and costs** | | | |
| --- | --- | --- | --- | --- | --- | --- | --- |
|  | **IU/IP cumulative HIV incidence (%)** | **Postnatal cumulative HIV incidence (%)** | **Total cumulative HIV incidence (%)** | **Undiscounted life expectancy (yrs)** | **Discounted life expectancy (yrs)** | **Discounted costs ($)** | **ICER ($/YLS)** |
| **South Africa [CET: ICER ≤ $1131/YLS (20% GDP per capita), ICER ≤ $2828/YLS (50% GDP per capita)]** | | | | | | | |
| ***Cost of identifying high-risk infants: $32*** | |  |  |  |  |  |  |
| Standard-of-care | 2·3 | 2·2 | 4·4 | 68·924 | 28·499 | 112 | Reference |
| HR-HIVE – 1 dose | 2·1 | 2·1 | 4·0 | 68·979 | 28·518 | 115 | dominated |
| HR-HIVE – 2 doses | 2·1 | 2·1 | 4·0 | 68·986 | 28·521 | 115 | dominated |
| HR-HIVE – Extended | 2·1 | 2·0 | 3·9 | 68·995 | 28·523 | 114 | dominated |
| HIVE – 1 dose | 2·0 | 2·0 | 3·8 | 69·011 | 28·529 | 105 | dominated |
| HIVE – 2 doses | 2·0 | 1·8 | 3·6 | 69·045 | 28·541 | 104 | dominated |
| ALL – 1 dose | 1·8 | 1·8 | 3·5 | 69·063 | 28·548 | 116 | dominated |
| HIVE – Extended | 2·0 | 1·5 | 3·3 | 69·095 | 28·557 | 100 | cost-saving |
| ALL – 2 doses | 1·8 | 1·5 | 3·2 | 69·122 | 28·568 | 122 | dominated |
| ALL – 1 dose plus HIVE - Extended | 1·8 | 1·3 | 3·0 | 69·147 | 28·575 | 111 | 606 |
| ALL - Extended | 1·8 | 0·8 | 2·5 | 69·228 | 28·602 | 135 | 882*^,^† |
| **Zimbabwe [CET: ICER ≤ $243/YLS (20% GDP per capita), ICER ≤ $607/YLS (50% GDP per capita)]** | | | | | | | |
| ***Cost of identifying high-risk infants: $0*** | | |  |  |  |  |  |
| Standard-of-care | 4·3 | 5·6 | 9·5 | 68·321 | 28·034 | 72 | Reference |
| HR-HIVE – 1 dose | 3·7 | 5·3 | 8·6 | 68·366 | 28·050 | 67 | dominated |
| HR-HIVE – 2 doses | 3·7 | 5·2 | 8·5 | 68·373 | 28·053 | 66 | dominated |
| HIVE – 1 dose | 3·7 | 5·2 | 8·4 | 68·380 | 28·055 | 67 | dominated |
| HR-HIVE – Extended | 3·7 | 4·9 | 8·2 | 68·387 | 28·057 | 65 | dominated |
| ALL – 1 dose | 3·4 | 4·9 | 7·9 | 68·400 | 28·062 | 79 | dominated |
| HIVE – 2 doses | 3·7 | 4·8 | 8·0 | 68·402 | 28·063 | 66 | dominated |
| ALL – 2 doses | 3·4 | 4·3 | 7·4 | 68·438 | 28·074 | 93 | dominated |
| HIVE – Extended | 3·7 | 3·6 | 6·9 | 68·458 | 28·081 | 61 | cost-saving*^,^† |
| ALL – 1 dose plus HIVE - Extended | 3·4 | 3·4 | 6·5 | 68·478 | 28·088 | 74 | 1731 |
| ALL - Extended | 3·4 | 2·6 | 5·7 | 68·517 | 28·101 | 134 | 4516 |
| ***Cost of identifying high-risk infants: $32*** | |  |  |  |  |  |  |
| Standard-of-care | 4·3 | 5·6 | 9·5 | 68·321 | 28·034 | 72 | Reference |
| HR-HIVE – 1 dose | 3·7 | 5·3 | 8·6 | 68·366 | 28·050 | 71 | dominated |
| HR-HIVE – 2 doses | 3·7 | 5·2 | 8·5 | 68·373 | 28·053 | 70 | dominated |
| HIVE – 1 dose | 3·7 | 5·2 | 8·4 | 68·380 | 28·055 | 67 | dominated |
| HR-HIVE – Extended | 3·7 | 4·9 | 8·2 | 68·387 | 28·057 | 69 | dominated |
| ALL – 1 dose | 3·4 | 4·9 | 7·9 | 68·400 | 28·062 | 79 | dominated |
| HIVE – 2 doses | 3·7 | 4·8 | 8·0 | 68·402 | 28·063 | 66 | dominated |
| ALL – 2 doses | 3·4 | 4·3 | 7·4 | 68·438 | 28·074 | 93 | dominated |
| HIVE – Extended | 3·7 | 3·6 | 6·9 | 68·458 | 28·081 | 61 | cost-saving*^,^† |
| ALL – 1 dose plus HIVE - Extended | 3·4 | 3·4 | 6·5 | 68·478 | 28·088 | 74 | 1731 |
| ALL - Extended | 3·4 | 2·6 | 5·7 | 68·517 | 28·101 | 134 | 4516 |

*bNAb: broadly neutralizing antibody; IU/IP: intrauterine/intrapartum; yr: year; ICER: incremental cost-effectiveness ratio; YLS: years of life saved; CET: cost-effectiveness threshold; HR-HIVE: high-risk HIV-exposed infants; HIVE: all HIV-exposed infants; ALL: all live infants at birth.*

The cost of ascertaining an infant’s HIV exposure risk status includes a maternal viral load test ($24·05), result return ($3·48), and personnel/overhead costs ($4·13).^26,27,149,151^ Pediatric HIV incidence is rounded to the nearest tenth of a percent. IU/IP HIV incidence is calculated based on the number of infants exposed to HIV at birth. Postnatal and total HIV incidence is calculated based on the number of infants ever exposed to HIV through 36 months of life. Undiscounted and discounted life expectancies are rounded to the nearest ten thousandth. Costs are rounded to the nearest dollar and are presented in 2020 USD. Discounted values are discounted at 3% per year. ICERs are rounded to the nearest dollar and are calculated using unrounded discounted life expectancy and discounted costs. The cost-effective bNAb strategy was the strategy that offered the greatest increase in overall population life expectancy while still having an ICER less than the cost-effectiveness threshold when compared to the next best performing, non-dominated strategy. * Indicates the cost-effective strategy using a cost-effectiveness threshold of 20% GDP per capita. † Indicates the cost-effective strategy using a cost-effectiveness threshold of 50% GDP per capita.

# **Supplementary Table 20.** One-way sensitivity analysis: maternal knowledge of acute HIV infection

| **Country/strategy** | **Clinical outcomes** | | | **Lifetime efficacy and costs** | | | |
| --- | --- | --- | --- | --- | --- | --- | --- |
|  | **IU/IP cumulative HIV incidence (%)** | **Postnatal cumulative HIV incidence (%)** | **Total cumulative HIV incidence (%)** | **Undiscounted life expectancy (yrs)** | **Discounted life expectancy (yrs)** | **Discounted costs ($)** | **ICER ($/YLS)** |
| **Côte d’Ivoire [CET: ICER ≤ $465/YLS (20% GDP per capita), ICER ≤ $1163/YLS (50% GDP per capita)]** | | | | | | | |
| ***Low maternal knowledge of acute HIV infection (in pregnancy: 25%; retesting postpartum: 0%/month)*** | | | | | | | |
| Standard-of-care | 3·7 | 5·0 | 8·4 | 60·128 | 26·373 | 15 | Reference |
| HR-HIVE – 1 dose | 3·4 | 4·9 | 8·1 | 60·132 | 26·374 | 15 | dominated |
| HR-HIVE – 2 doses | 3·4 | 4·8 | 7·9 | 60·133 | 26·375 | 15 | dominated |
| HIVE – 1 dose | 3·3 | 4·8 | 7·9 | 60·133 | 26·375 | 14 | dominated |
| HR-HIVE – Extended | 3·4 | 4·4 | 7·6 | 60·135 | 26·375 | 14 | dominated |
| HIVE – 2 doses | 3·3 | 4·4 | 7·5 | 60·136 | 26·376 | 14 | dominated |
| ALL – 1 dose | 3·0 | 4·7 | 7·6 | 60·138 | 26·377 | 27 | dominated |
| ALL – 2 doses | 3·0 | 4·2 | 7·1 | 60·139 | 26·378 | 44 | dominated |
| HIVE – Extended | 3·3 | 3·2 | 6·4 | 60·144 | 26·379 | 13 | cost-saving*^,^† |
| ALL – 1 dose plus HIVE - Extended | 3·0 | 3·2 | 6·1 | 60·149 | 26·381 | 26 | 6012 |
| ALL - Extended | 3·0 | 2·7 | 5·6 | 60·155 | 26·382 | 91 | 55 092 |
| ***High maternal knowledge of acute HIV infection* *(in pregnancy: 95%; retesting postpartum: 15%/month)*** | | | | | | | |
| Standard-of-care | 3·6 | 4·6 | 8·0 | 60·133 | 26·375 | 14 | Reference |
| HR-HIVE – 1 dose | 3·3 | 4·5 | 7·6 | 60·136 | 26·376 | 14 | dominated |
| HR-HIVE – 2 doses | 3·3 | 4·4 | 7·5 | 60·137 | 26·377 | 14 | dominated |
| HIVE – 1 dose | 3·2 | 4·4 | 7·4 | 60·138 | 26·377 | 14 | dominated |
| HR-HIVE – Extended | 3·3 | 4·0 | 7·1 | 60·140 | 26·377 | 14 | dominated |
| HIVE – 2 doses | 3·2 | 4·0 | 7·0 | 60·141 | 26·378 | 13 | dominated |
| ALL – 1 dose | 3·0 | 4·3 | 7·1 | 60·143 | 26·379 | 27 | dominated |
| ALL – 2 doses | 3·0 | 3·8 | 6·7 | 60·143 | 26·379 | 42 | dominated |
| HIVE – Extended | 3·2 | 2·8 | 5·9 | 60·149 | 26·381 | 12 | cost-saving*^,^† |
| ALL – 1 dose plus HIVE - Extended | 3·0 | 2·8 | 5·6 | 60·154 | 26·383 | 26 | 6779 |
| ALL - Extended | 3·0 | 2·4 | 5·3 | 60·159 | 26·384 | 91 | 79 100 |
| **South Africa [CET: ICER ≤ $1131/YLS (20% GDP per capita), ICER ≤ $2828/YLS (50% GDP per capita)]** | | | | | | | |
| ***Low maternal knowledge of acute HIV infection (in pregnancy: 25%; retesting postpartum: 0%/month)*** | | | | | | | |
| Standard-of-care | 2·7 | 2·2 | 4·7 | 68·851 | 28·472 | 118 | Reference |
| HR-HIVE – 1 dose | 2·4 | 2·1 | 4·3 | 68·905 | 28·491 | 121 | dominated |
| HR-HIVE – 2 doses | 2·4 | 2·1 | 4·3 | 68·911 | 28·493 | 121 | dominated |
| HR-HIVE – Extended | 2·4 | 2·0 | 4·3 | 68·919 | 28·495 | 120 | dominated |
| HIVE – 1 dose | 2·3 | 2·0 | 4·1 | 68·937 | 28·502 | 111 | dominated |
| HIVE – 2 doses | 2·3 | 1·8 | 4·0 | 68·970 | 28·513 | 110 | dominated |
| ALL – 1 dose | 2·1 | 1·8 | 3·7 | 69·000 | 28·524 | 120 | dominated |
| HIVE – Extended | 2·3 | 1·5 | 3·7 | 69·019 | 28·529 | 107 | cost-saving |
| ALL – 2 doses | 2·1 | 1·5 | 3·4 | 69·060 | 28·545 | 127 | dominated |
| ALL – 1 dose plus HIVE - Extended | 2·1 | 1·3 | 3·2 | 69·082 | 28·551 | 116 | 405 |
| ALL - Extended | 2·1 | 0·8 | 2·8 | 69·164 | 28·578 | 139 | 875*^,^† |

**Supplementary Table 20.** One-way sensitivity analysis: HIV knowledge of acute infection (cont.)

| **Country/strategy** | **Clinical outcomes** | | | **Lifetime efficacy and costs** | | | |
| --- | --- | --- | --- | --- | --- | --- | --- |
|  | **IU/IP cumulative HIV incidence (%)** | **Postnatal cumulative HIV incidence (%)** | **Total cumulative HIV incidence (%)** | **Undiscounted life expectancy (yrs)** | **Discounted life expectancy (yrs)** | **Discounted costs ($)** | **ICER ($/YLS)** |
| **South Africa [CET: ICER ≤ $1131/YLS (20% GDP per capita), ICER ≤ $2828/YLS (50% GDP per capita)]** | | | | | | | |
| ***High maternal knowledge of acute HIV infection* *(in pregnancy: 95%; retesting postpartum: 15%/month)*** | | | | | | | |
| Standard-of-care | 1·9 | 2·0 | 3·8 | 69·051 | 28·546 | 101 | Reference |
| HR-HIVE – 1 dose | 1·6 | 1·9 | 3·4 | 69·109 | 28·566 | 104 | dominated |
| HR-HIVE – 2 doses | 1·6 | 1·8 | 3·4 | 69·117 | 28·568 | 104 | dominated |
| HR-HIVE – Extended | 1·6 | 1·8 | 3·3 | 69·127 | 28·571 | 104 | dominated |
| HIVE – 1 dose | 1·5 | 1·8 | 3·2 | 69·140 | 28·577 | 94 | dominated |
| ALL – 1 dose | 1·5 | 1·7 | 3·1 | 69·161 | 28·584 | 109 | dominated |
| HIVE – 2 doses | 1·5 | 1·6 | 3·0 | 69·175 | 28·589 | 93 | dominated |
| ALL – 2 doses | 1·5 | 1·4 | 2·8 | 69·217 | 28·603 | 115 | dominated |
| HIVE – Extended | 1·5 | 1·3 | 2·7 | 69·226 | 28·605 | 90 | cost-saving* |
| ALL – 1 dose plus HIVE - Extended | 1·5 | 1·1 | 2·6 | 69·247 | 28·612 | 104 | dominated |
| ALL - Extended | 1·5 | 0·7 | 2·1 | 69·320 | 28·636 | 128 | 1221† |
| **Zimbabwe [CET: ICER ≤ $243/YLS (20% GDP per capita), ICER ≤ $607/YLS (50% GDP per capita)]** | | | | | | | |
| ***Low maternal knowledge of acute HIV infection (in pregnancy: 25%; retesting postpartum: 0%/month)*** | | | | | | | |
| Standard-of-care | 4·9 | 6·6 | 10·9 | 68·213 | 27·994 | 79 | Reference |
| HR-HIVE – 1 dose | 4·5 | 6·4 | 10·3 | 68·244 | 28·005 | 79 | dominated |
| HR-HIVE – 2 doses | 4·5 | 6·3 | 10·2 | 68·249 | 28·007 | 79 | dominated |
| HIVE – 1 dose | 4·4 | 6·3 | 10·1 | 68·258 | 28·010 | 76 | dominated |
| HR-HIVE – Extended | 4·5 | 6·1 | 10·0 | 68·260 | 28·010 | 78 | dominated |
| HIVE – 2 doses | 4·4 | 5·9 | 9·7 | 68·278 | 28·017 | 75 | dominated |
| ALL – 1 dose | 3·9 | 5·6 | 9·1 | 68·310 | 28·029 | 85 | dominated |
| HIVE – Extended | 4·4 | 4·8 | 8·7 | 68·330 | 28·034 | 70 | cost-saving* |
| ALL – 2 doses | 3·9 | 5·0 | 8·5 | 68·351 | 28·042 | 99 | dominated |
| ALL – 1 dose plus HIVE - Extended | 3·9 | 4·2 | 7·7 | 68·383 | 28·053 | 80 | 520† |
| ALL - Extended | 3·9 | 3·1 | 6·6 | 68·438 | 28·072 | 138 | 3081 |
| ***High maternal knowledge of acute HIV infection* *(in pregnancy: 95%; retesting postpartum: 15%/month)*** | | | | | | | |
| Standard-of-care | 4·0 | 5·0 | 8·6 | 68·383 | 28·056 | 67 | Reference |
| HR-HIVE – 1 dose | 3·4 | 4·6 | 7·6 | 68·436 | 28·075 | 65 | dominated |
| HR-HIVE – 2 doses | 3·4 | 4·5 | 7·5 | 68·444 | 28·078 | 64 | dominated |
| HIVE – 1 dose | 3·3 | 4·5 | 7·4 | 68·449 | 28·080 | 61 | dominated |
| ALL – 1 dose | 3·2 | 4·4 | 7·2 | 68·454 | 28·082 | 75 | dominated |
| HR-HIVE – Extended | 3·4 | 4·2 | 7·2 | 68·460 | 28·083 | 63 | dominated |
| HIVE – 2 doses | 3·3 | 4·1 | 7·0 | 68·473 | 28·088 | 60 | dominated |
| ALL – 2 doses | 3·2 | 3·9 | 6·7 | 68·490 | 28·093 | 89 | dominated |
| HIVE – Extended | 3·3 | 2·9 | 5·9 | 68·530 | 28·107 | 55 | cost-saving*^,^† |
| ALL – 1 dose plus HIVE - Extended | 3·2 | 2·8 | 5·7 | 68·534 | 28·108 | 70 | dominated |
| ALL - Extended | 3·2 | 2·3 | 5·2 | 68·561 | 28·117 | 130 | 7013 |

*bNAb: broadly neutralizing antibody; IU/IP: intrauterine/intrapartum; yr: year; ICER: incremental cost-effectiveness ratio; YLS: years of life saved; CET: cost-effectiveness threshold; HR-HIVE: high-risk HIV-exposed infants; HIVE: all HIV-exposed infants; ALL: all live infants at birth.*

Pediatric HIV incidence is rounded to the nearest tenth of a percent. IU/IP HIV incidence is calculated based on the number of infants exposed to HIV at birth. Postnatal and total HIV incidence is calculated based on the number of infants ever exposed to HIV through 36 months of life. Undiscounted and discounted life expectancies are rounded to the nearest ten thousandth. Costs are rounded to the nearest dollar and are presented in 2020 USD. Discounted values are discounted at 3% per year. ICERs are rounded to the nearest dollar and are calculated using unrounded discounted life expectancy and discounted costs. The cost-effective bNAb strategy was the strategy that offered the greatest increase in overall population life expectancy while still having an ICER less than the cost-effectiveness threshold when compared to the next best performing, non-dominated strategy. * Indicates the cost-effective strategy using a cost-effectiveness threshold of 20% GDP per capita. † Indicates the cost-effective strategy using a cost-effectiveness threshold of 50% GDP per capita.

# **Supplementary Table 21.** One-way sensitivity analysis: maternal knowledge of chronic HIV infection

| **Country/strategy** | **Clinical outcomes** | | | **Lifetime efficacy and costs** | | | |
| --- | --- | --- | --- | --- | --- | --- | --- |
|  | **IU/IP cumulative HIV incidence (%)** | **Postnatal cumulative HIV incidence (%)** | **Total cumulative HIV incidence (%)** | **Undiscounted life expectancy (yrs)** | **Discounted life expectancy (yrs)** | **Discounted costs ($)** | **ICER ($/YLS)** |
| **Côte d’Ivoire [CET: ICER ≤ $465/YLS (20% GDP per capita), ICER ≤ $1163/YLS (50% GDP per capita)]** | | | | | | | |
| ***Low maternal knowledge of chronic HIV infection: 56%*** | | | |  |  |  |  |
| Standard-of-care | 11·0 | 8·1 | 18·9 | 60·020 | 26·329 | 27 | Reference |
| HR-HIVE – 1 dose | 10·8 | 8·1 | 18·6 | 60·022 | 26·329 | 27 | dominated |
| HR-HIVE – 2 doses | 10·8 | 8·0 | 18·5 | 60·023 | 26·330 | 27 | dominated |
| HIVE – 1 dose | 10·7 | 8·0 | 18·5 | 60·023 | 26·330 | 27 | dominated |
| HR-HIVE – Extended | 10·8 | 7·7 | 18·3 | 60·025 | 26·330 | 27 | dominated |
| HIVE – 2 doses | 10·7 | 7·7 | 18·3 | 60·025 | 26·331 | 27 | dominated |
| HIVE – Extended | 10·7 | 7·0 | 17·5 | 60·030 | 26·332 | 26 | cost-saving*^,^† |
| ALL – 1 dose | 9·0 | 7·6 | 16·5 | 60·041 | 26·337 | 39 | dominated |
| ALL – 2 doses | 9·0 | 6·9 | 15·7 | 60·044 | 26·339 | 54 | dominated |
| ALL – 1 dose plus HIVE - Extended | 9·0 | 6·7 | 15·5 | 60·048 | 26·340 | 38 | 1587 |
| ALL - Extended | 9·0 | 4·5 | 13·4 | 60·065 | 26·345 | 100 | 11 429 |
| ***High maternal knowledge of chronic HIV infection: 100%*** | | | |  |  |  |  |
| Standard-of-care | 2·4 | 4·2 | 6·6 | 60·149 | 26·381 | 12 | Reference |
| HR-HIVE – 1 dose | 2·1 | 4·1 | 6·2 | 60·153 | 26·383 | 13 | dominated |
| HR-HIVE – 2 doses | 2·1 | 4·0 | 6·0 | 60·154 | 26·383 | 13 | dominated |
| HIVE – 1 dose | 2·0 | 4·0 | 6·0 | 60·155 | 26·384 | 12 | dominated |
| HR-HIVE – Extended | 2·1 | 3·6 | 5·7 | 60·157 | 26·384 | 12 | dominated |
| ALL – 1 dose | 2·0 | 4·0 | 5·9 | 60·157 | 26·385 | 25 | dominated |
| HIVE – 2 doses | 2·0 | 3·6 | 5·6 | 60·158 | 26·385 | 12 | dominated |
| ALL – 2 doses | 2·0 | 3·6 | 5·5 | 60·158 | 26·385 | 41 | dominated |
| HIVE – Extended | 2·0 | 2·4 | 4·4 | 60·166 | 26·388 | 10 | cost-saving*^,^† |
| ALL – 1 dose plus HIVE - Extended | 2·0 | 2·4 | 4·3 | 60·169 | 26·389 | 24 | 11 617 |
| ALL - Extended | 2·0 | 2·2 | 4·2 | 60·173 | 26·390 | 89 | 197 506 |
| **South Africa [CET: ICER ≤ $1131/YLS (20% GDP per capita), ICER ≤ $2828/YLS (50% GDP per capita)]** | | | | | | | |
| ***Low maternal knowledge of chronic HIV infection: 75%*** | | | |  |  |  |  |
| Standard-of-care | 6·9 | 3·3 | 9·8 | 67·760 | 28·071 | 207 | Reference |
| HR-HIVE – 1 dose | 6·7 | 3·2 | 9·5 | 67·807 | 28·087 | 209 | dominated |
| HR-HIVE – 2 doses | 6·7 | 3·1 | 9·5 | 67·812 | 28·089 | 209 | dominated |
| HR-HIVE – Extended | 6·7 | 3·1 | 9·4 | 67·819 | 28·091 | 208 | dominated |
| HIVE – 1 dose | 6·6 | 3·1 | 9·3 | 67·831 | 28·095 | 201 | dominated |
| HIVE – 2 doses | 6·6 | 2·9 | 9·2 | 67·857 | 28·104 | 200 | dominated |
| HIVE – Extended | 6·6 | 2·7 | 8·9 | 67·895 | 28·117 | 198 | dominated |
| ALL – 1 dose | 5·3 | 2·7 | 7·7 | 68·064 | 28·178 | 193 | dominated |
| ALL – 1 dose plus HIVE - Extended | 5·3 | 2·3 | 7·3 | 68·128 | 28·199 | 189 | cost-saving |
| ALL – 2 doses | 5·3 | 2·2 | 7·2 | 68·149 | 28·207 | 196 | dominated |
| ALL - Extended | 5·3 | 1·2 | 6·2 | 68·302 | 28·257 | 203 | 239*^,^† |

**Supplementary Table 21.** One-way sensitivity analysis: maternal knowledge of chronic HIV infection (cont.)

| **Country/strategy** | **Clinical outcomes** | | | **Lifetime efficacy and costs** | | | |
| --- | --- | --- | --- | --- | --- | --- | --- |
|  | **IU/IP cumulative HIV incidence (%)** | **Postnatal cumulative HIV incidence (%)** | **Total cumulative HIV incidence (%)** | **Undiscounted life expectancy (yrs)** | **Discounted life expectancy (yrs)** | **Discounted costs ($)** | **ICER ($/YLS)** |
| **South Africa [CET: ICER ≤ $1131/YLS (20% GDP per capita), ICER ≤ $2828/YLS (50% GDP per capita)]** | | | | | | | |
| ***High maternal knowledge of chronic HIV infection: 100%*** | | | |  |  |  |  |
| Standard-of-care | 2·3 | 2·1 | 4·3 | 68·946 | 28·507 | 110 | Reference |
| HR-HIVE – 1 dose | 2·0 | 2·0 | 3·9 | 69·000 | 28·526 | 114 | dominated |
| HR-HIVE – 2 doses | 2·0 | 2·0 | 3·9 | 69·007 | 28·528 | 113 | dominated |
| HR-HIVE – Extended | 2·0 | 1·9 | 3·8 | 69·016 | 28·531 | 113 | dominated |
| HIVE – 1 dose | 1·9 | 1·9 | 3·7 | 69·034 | 28·538 | 103 | dominated |
| HIVE – 2 doses | 1·9 | 1·7 | 3·5 | 69·068 | 28·549 | 102 | dominated |
| ALL – 1 dose | 1·7 | 1·7 | 3·4 | 69·082 | 28·555 | 115 | dominated |
| HIVE – Extended | 1·9 | 1·4 | 3·2 | 69·118 | 28·566 | 99 | cost-saving |
| ALL – 2 doses | 1·7 | 1·4 | 3·1 | 69·140 | 28·575 | 121 | dominated |
| ALL – 1 dose plus HIVE - Extended | 1·7 | 1·2 | 2·9 | 69·166 | 28·583 | 110 | 674 |
| ALL - Extended | 1·7 | 0·8 | 2·4 | 69·245 | 28·608 | 134 | 909*^,^† |
| **Zimbabwe [CET: ICER ≤ $243/YLS (20% GDP per capita), ICER ≤ $607/YLS (50% GDP per capita)]** | | | | | | | |
| \| ***Low maternal knowledge of chronic HIV infection: 80%*** \| \| --- \| | | | |  |  |  |  |
| Standard-of-care | 7·3 | 6·6 | 13·4 | 68·047 | 27·933 | 94 | Reference |
| HR-HIVE – 1 dose | 6·8 | 6·3 | 12·6 | 68·088 | 27·947 | 93 | dominated |
| HR-HIVE – 2 doses | 6·8 | 6·2 | 12·5 | 68·094 | 27·950 | 93 | dominated |
| HIVE – 1 dose | 6·7 | 6·2 | 12·5 | 68·099 | 27·951 | 90 | dominated |
| HR-HIVE – Extended | 6·8 | 6·0 | 12·3 | 68·106 | 27·954 | 92 | dominated |
| HIVE – 2 doses | 6·7 | 5·9 | 12·1 | 68·117 | 27·958 | 89 | dominated |
| HIVE – Extended | 6·7 | 4·9 | 11·2 | 68·164 | 27·973 | 85 | cost-saving* |
| ALL – 1 dose | 5·8 | 5·8 | 11·2 | 68·159 | 27·973 | 99 | dominated |
| ALL – 2 doses | 5·8 | 5·1 | 10·5 | 68·203 | 27·987 | 112 | dominated |
| ALL – 1 dose plus HIVE - Extended | 5·8 | 4·6 | 9·9 | 68·224 | 27·994 | 94 | 410† |
| ALL - Extended | 5·8 | 3·1 | 8·5 | 68·298 | 28·019 | 150 | 2238 |
| ***High maternal knowledge of chronic HIV infection: 100%*** | | | |  |  |  |  |
| Standard-of-care | 4·0 | 5·3 | 9·1 | 68·348 | 28·044 | 69 | Reference |
| HR-HIVE – 1 dose | 3·4 | 5·0 | 8·2 | 68·397 | 28·062 | 68 | dominated |
| HR-HIVE – 2 doses | 3·4 | 4·9 | 8·1 | 68·404 | 28·064 | 68 | dominated |
| HIVE – 1 dose | 3·4 | 4·9 | 8·0 | 68·408 | 28·065 | 64 | dominated |
| HR-HIVE – Extended | 3·4 | 4·6 | 7·8 | 68·418 | 28·069 | 67 | dominated |
| ALL – 1 dose | 3·2 | 4·6 | 7·6 | 68·424 | 28·071 | 77 | dominated |
| HIVE – 2 doses | 3·4 | 4·5 | 7·6 | 68·430 | 28·073 | 64 | dominated |
| ALL – 2 doses | 3·2 | 4·1 | 7·1 | 68·462 | 28·083 | 91 | dominated |
| HIVE – Extended | 3·4 | 3·4 | 6·5 | 68·487 | 28·091 | 59 | cost-saving*^,^† |
| ALL – 1 dose plus HIVE - Extended | 3·2 | 3·1 | 6·1 | 68·503 | 28·097 | 72 | 2215 |
| ALL - Extended | 3·2 | 2·4 | 5·4 | 68·539 | 28·109 | 132 | 4077 |

*bNAb: broadly neutralizing antibody; IU/IP: intrauterine/intrapartum; yr: year; ICER: incremental cost-effectiveness ratio; YLS: years of life saved; CET: cost-effectiveness threshold; HR-HIVE: high-risk HIV-exposed infants; HIVE: all HIV-exposed infants; ALL: all live infants at birth.*

Pediatric HIV incidence is rounded to the nearest tenth of a percent. IU/IP HIV incidence is calculated based on the number of infants exposed to HIV at birth. Postnatal and total HIV incidence is calculated based on the number of infants ever exposed to HIV through 36 months of life. Undiscounted and discounted life expectancies are rounded to the nearest ten thousandth. Costs are rounded to the nearest dollar and are presented in 2020 USD. Discounted values are discounted at 3% per year. ICERs are rounded to the nearest dollar and are calculated using unrounded discounted life expectancy and discounted costs. The cost-effective bNAb strategy was the strategy that offered the greatest increase in overall population life expectancy while still having an ICER less than the cost-effectiveness threshold when compared to the next best performing, non-dominated strategy. *Indicates the cost-effective strategy using a cost-effectiveness threshold of 20% GDP per capita. †Indicates the cost-effective strategy using a cost-effectiveness threshold of 50% GDP per capita.

# **Supplementary Table 22.** One-way sensitivity analysis: postpartum maternal retention in care

| **Country/strategy** | **Clinical outcomes** | | | **Lifetime efficacy and costs** | | | |
| --- | --- | --- | --- | --- | --- | --- | --- |
|  | **IU/IP cumulative HIV incidence (%)** | **Postnatal cumulative HIV incidence (%)** | **Total cumulative HIV incidence (%)** | **Undiscounted life expectancy (yrs)** | **Discounted life expectancy (yrs)** | **Discounted costs ($)** | **ICER ($/YLS)** |
| **Côte d’Ivoire [CET: ICER ≤ $465/YLS (20% GDP per capita), ICER ≤ $1163/YLS (50% GDP per capita)]** | | | | | | | |
| ***Low postpartum maternal retention in care (50% of mothers with known HIV status in care at 6+ months)*** | | | |  |  |  |  |
| Standard-of-care | 3·6 | 7·1 | 10·4 | 60·114 | 26·368 | 18 | Reference |
| HR-HIVE – 1 dose | 3·3 | 7·0 | 10·0 | 60·118 | 26·369 | 19 | dominated |
| HR-HIVE – 2 doses | 3·3 | 6·8 | 9·8 | 60·119 | 26·370 | 18 | dominated |
| HIVE – 1 dose | 3·3 | 6·8 | 9·8 | 60·120 | 26·370 | 18 | dominated |
| HR-HIVE – Extended | 3·3 | 6·2 | 9·2 | 60·123 | 26·371 | 18 | dominated |
| HIVE – 2 doses | 3·3 | 6·2 | 9·2 | 60·125 | 26·372 | 17 | dominated |
| ALL – 1 dose | 3·0 | 6·8 | 9·5 | 60·125 | 26·372 | 31 | dominated |
| ALL – 2 doses | 3·0 | 6·0 | 8·8 | 60·127 | 26·373 | 47 | dominated |
| HIVE – Extended | 3·3 | 4·3 | 7·4 | 60·138 | 26·377 | 15 | cost-saving*^,^† |
| ALL – 1 dose plus HIVE - Extended | 3·0 | 4·2 | 7·1 | 60·143 | 26·379 | 28 | 6245 |
| ALL - Extended | 3·0 | 3·8 | 6·7 | 60·148 | 26·380 | 93 | 57 575 |
| ***High postpartum maternal retention in care (100% of mothers with known HIV status in care at 6+ months)*** | | | |  |  |  |  |
| Standard-of-care | 3·6 | 4·1 | 7·5 | 60·135 | 26·375 | 13 | Reference |
| HR-HIVE – 1 dose | 3·3 | 4·0 | 7·1 | 60·139 | 26·377 | 13 | dominated |
| HR-HIVE – 2 doses | 3·3 | 3·9 | 7·0 | 60·140 | 26·377 | 13 | dominated |
| HIVE – 1 dose | 3·3 | 3·9 | 6·9 | 60·140 | 26·378 | 13 | dominated |
| HR-HIVE – Extended | 3·3 | 3·6 | 6·7 | 60·142 | 26·378 | 13 | dominated |
| HIVE – 2 doses | 3·3 | 3·5 | 6·6 | 60·143 | 26·379 | 12 | dominated |
| ALL – 1 dose | 3·0 | 3·8 | 6·6 | 60·145 | 26·380 | 26 | dominated |
| ALL – 2 doses | 3·0 | 3·4 | 6·3 | 60·145 | 26·380 | 41 | dominated |
| HIVE – Extended | 3·3 | 2·7 | 5·8 | 60·149 | 26·381 | 12 | cost-saving*^,^† |
| ALL – 1 dose plus HIVE - Extended | 3·0 | 2·6 | 5·5 | 60·154 | 26·383 | 25 | 6264 |
| ALL - Extended | 3·0 | 2·2 | 5·1 | 60·160 | 26·384 | 90 | 57 976 |
| **South Africa [CET: ICER ≤ $1131/YLS (20% GDP per capita), ICER ≤ $2828/YLS (50% GDP per capita)]** | | | | | | | |
| ***Low postpartum maternal retention in care (50% of mothers with known HIV status in care at 6+ months)*** | | | |  |  |  |  |
| Standard-of-care | 2·3 | 2·8 | 5·0 | 68·827 | 28·467 | 125 | Reference |
| HR-HIVE – 1 dose | 2·1 | 2·7 | 4·6 | 68·883 | 28·486 | 128 | dominated |
| HR-HIVE – 2 doses | 2·1 | 2·6 | 4·5 | 68·893 | 28·490 | 127 | dominated |
| HR-HIVE – Extended | 2·1 | 2·5 | 4·4 | 68·908 | 28·494 | 126 | dominated |
| HIVE – 1 dose | 2·0 | 2·6 | 4·4 | 68·919 | 28·499 | 117 | dominated |
| ALL – 1 dose | 1·8 | 2·3 | 4·0 | 68·970 | 28·517 | 128 | dominated |
| HIVE – 2 doses | 2·0 | 2·2 | 4·1 | 68·973 | 28·517 | 114 | dominated |
| HIVE – Extended | 1·8 | 1·9 | 3·6 | 69·050 | 28·544 | 132 | dominated |
| ALL – 2 doses | 2·0 | 1·7 | 3·5 | 69·057 | 28·545 | 106 | cost-saving |
| ALL – 1 dose plus HIVE - Extended | 1·8 | 1·5 | 3·2 | 69·109 | 28·563 | 116 | 575 |
| ALL - Extended | 1·8 | 1·0 | 2·7 | 69·192 | 28·590 | 139 | 847*^,^† |

**Supplementary Table 22.** One-way sensitivity analysis: postpartum maternal retention in care (cont.)

| **Country/strategy** | **Clinical outcomes** | | | **Lifetime efficacy and costs** | | | |
| --- | --- | --- | --- | --- | --- | --- | --- |
|  | **IU/IP cumulative HIV incidence (%)** | **Postnatal cumulative HIV incidence (%)** | **Total cumulative HIV incidence (%)** | **Undiscounted life expectancy (yrs)** | **Discounted life expectancy (yrs)** | **Discounted costs ($)** | **ICER ($/YLS)** |
| **South Africa [CET: ICER ≤ $1131/YLS (20% GDP per capita), ICER ≤ $2828/YLS (50% GDP per capita)]** | | | | | | | |
| ***High postpartum maternal retention in care (100% of mothers with known HIV status in care at 6+ months)*** | | | |  |  |  |  |
| Standard-of-care | 2·3 | 2·0 | 4·2 | 68·956 | 28·510 | 108 | Reference |
| HR-HIVE – 1 dose | 2·1 | 1·9 | 3·8 | 69·011 | 28·529 | 111 | dominated |
| HR-HIVE – 2 doses | 2·1 | 1·9 | 3·8 | 69·017 | 28·531 | 111 | dominated |
| HR-HIVE – Extended | 2·1 | 1·8 | 3·7 | 69·024 | 28·533 | 110 | dominated |
| HIVE – 1 dose | 2·0 | 1·8 | 3·6 | 69·043 | 28·540 | 101 | dominated |
| ALL – 1 dose | 2·0 | 1·6 | 3·5 | 69·070 | 28·549 | 100 | dominated |
| HIVE – 2 doses | 1·8 | 1·6 | 3·3 | 69·094 | 28·558 | 111 | dominated |
| ALL – 2 doses | 2·0 | 1·4 | 3·2 | 69·108 | 28·561 | 99 | cost-saving |
| HIVE – Extended | 1·8 | 1·3 | 3·0 | 69·147 | 28·576 | 118 | dominated |
| ALL – 1 dose plus HIVE - Extended | 1·8 | 1·2 | 2·9 | 69·160 | 28·580 | 109 | 569 |
| ALL - Extended | 1·8 | 0·7 | 2·4 | 69·240 | 28·606 | 133 | 921*^,^† |
| **Zimbabwe [CET: ICER ≤ $243/YLS (20% GDP per capita), ICER ≤ $607/YLS (50% GDP per capita)]** | | | | | | | |
| \| ***Low postpartum maternal retention in care (50% of mothers with known HIV status in care at 6+ months)*** \| \| --- \| | | | |  |  |  |  |
| Standard-of-care | 4·3 | 7·5 | 11·3 | 68·227 | 28·003 | 86 | Reference |
| HR-HIVE – 1 dose | 3·7 | 7·2 | 10·4 | 68·271 | 28·019 | 85 | dominated |
| HR-HIVE – 2 doses | 3·7 | 7·0 | 10·2 | 68·282 | 28·022 | 84 | dominated |
| HIVE – 1 dose | 3·7 | 7·0 | 10·2 | 68·288 | 28·025 | 81 | dominated |
| HR-HIVE – Extended | 3·7 | 6·5 | 9·8 | 68·305 | 28·030 | 81 | dominated |
| ALL – 1 dose | 3·4 | 6·7 | 9·7 | 68·308 | 28·032 | 93 | dominated |
| HIVE – 2 doses | 3·7 | 6·4 | 9·6 | 68·322 | 28·037 | 78 | dominated |
| ALL – 2 doses | 3·4 | 5·9 | 9·0 | 68·358 | 28·048 | 106 | dominated |
| HIVE – Extended | 3·7 | 4·5 | 7·8 | 68·415 | 28·066 | 68 | cost-saving*^,^† |
| ALL – 1 dose plus HIVE - Extended | 3·4 | 4·2 | 7·3 | 68·435 | 28·074 | 81 | 1738 |
| ALL - Extended | 3·4 | 3·4 | 6·5 | 68·474 | 28·087 | 140 | 4438 |
| ***High postpartum maternal retention in care (100% of mothers with known HIV status in care at 6+ months)*** | | | |  |  |  |  |
| Standard-of-care | 4·3 | 4·9 | 8·8 | 68·356 | 28·045 | 66 | Reference |
| HR-HIVE – 1 dose | 3·7 | 4·6 | 7·9 | 68·401 | 28·062 | 65 | dominated |
| HR-HIVE – 2 doses | 3·7 | 4·5 | 7·8 | 68·407 | 28·064 | 65 | dominated |
| HIVE – 1 dose | 3·7 | 4·5 | 7·7 | 68·415 | 28·066 | 61 | dominated |
| HR-HIVE – Extended | 3·7 | 4·3 | 7·6 | 68·418 | 28·067 | 64 | dominated |
| HIVE – 2 doses | 3·7 | 4·1 | 7·4 | 68·433 | 28·073 | 61 | dominated |
| ALL – 1 dose | 3·4 | 4·2 | 7·3 | 68·435 | 28·074 | 74 | dominated |
| ALL – 2 doses | 3·4 | 3·7 | 6·8 | 68·469 | 28·084 | 88 | dominated |
| HIVE – Extended | 3·7 | 3·3 | 6·6 | 68·475 | 28·086 | 59 | cost-saving*^,^† |
| ALL – 1 dose plus HIVE - Extended | 3·4 | 3·0 | 6·1 | 68·495 | 28·093 | 71 | 1724 |
| ALL - Extended | 3·4 | 2·2 | 5·4 | 68·534 | 28·107 | 131 | 4539 |

*bNAb: broadly neutralizing antibody; IU/IP: intrauterine/intrapartum; yr: year; ICER: incremental cost-effectiveness ratio; YLS: years of life saved; CET: cost-effectiveness threshold; HR-HIVE: high-risk HIV-exposed infants; HIVE: all HIV-exposed infants; ALL: all live infants at birth.*

Pediatric HIV incidence is rounded to the nearest tenth of a percent. IU/IP HIV incidence is calculated based on the number of infants exposed to HIV at birth. Postnatal and total HIV incidence is calculated based on the number of infants ever exposed to HIV through 36 months of life. Undiscounted and discounted life expectancies are rounded to the nearest ten thousandth. Costs are rounded to the nearest dollar and are presented in 2020 USD. Discounted values are discounted at 3% per year. ICERs are rounded to the nearest dollar and are calculated using unrounded discounted life expectancy and discounted costs. The cost-effective bNAb strategy was the strategy that offered the greatest increase in overall population life expectancy while still having an ICER less than the cost-effectiveness threshold when compared to the next best performing, non-dominated strategy. *Indicates the cost-effective strategy using a cost-effectiveness threshold of 20% GDP per capita. †Indicates the cost-effective strategy using a cost-effectiveness threshold of 50% GDP per capita.

# **References**

1 Ciaranello AL, Morris BL, Walensky RP, Weinstein MC, Ayaya S, Doherty K, et al. Validation and calibration of a computer simulation model of pediatric HIV infection. PLoS one. 2013 Dec 13;8(12):e83389.

2 Ciaranello AL, Doherty K, Penazzato M, Lindsey JC, Harrison L, Kelly K, et al. Cost-effectiveness of first-line antiretroviral therapy for HIV-infected African children less than 3 years of age. AIDS. 2015 Jun 6;29(10):1247.

3 Francke JA, Penazzato M, Hou T, Abrams EJ, MacLean RL, Myer L, et al. Clinical impact and cost-effectiveness of diagnosing HIV infection during early infancy in South Africa: test timing and frequency. J Infect Dis. 2016 Nov 1;214(9):1319-28.

4 Frank SC, Cohn J, Dunning L, Sacks E, Walensky RP, Mukherjee S, et al. Clinical effect and cost-effectiveness of incorporation of point-of-care assays into early infant HIV diagnosis programmes in Zimbabwe: a modelling study. Lancet HIV. 2019 Mar 1;6(3):e182-90.

5 Walensky RP, Borre ED, Bekker LG, Resch SC, Hyle EP, Wood R, et al. The anticipated clinical and economic effects of 90–90–90 in South Africa. Ann Intern Med. 2016 Sep 6;165(5):325-33.

6 Ciaranello AL, Perez F, Keatinge J, Park JE, Engelsmann B, Maruva M, et al. What will it take to eliminate pediatric HIV? Reaching WHO target rates of mother-to-child HIV transmission in Zimbabwe: a model-based analysis. PLoS Med. 2012 Jan 10;9(1):e1001156.

7 Dugdale CM, Ciaranello AL, Bekker LG, Stern ME, Myer L, Wood R, et al. Risks and benefits of dolutegravir-and efavirenz-based strategies for South African women with HIV of child-bearing potential: a modeling study. Ann Intern Med. 2019 May 7;170(9):614-25.

8 Dunning L, Francke JA, Mallampati D, MacLean RL, Penazzato M, Hou T, et al. The value of confirmatory testing in early infant HIV diagnosis programmes in South Africa: a cost-effectiveness analysis. PLoS Med. 2017 Nov 21;14(11):e1002446.

9 Dunning L, Gandhi AR, Penazzato M, Soeteman DI, Revill P, Frank S, et al. Optimizing infant HIV diagnosis with additional screening at immunization clinics in three sub‐Saharan African settings: a cost‐effectiveness analysis. J Int AIDS Soc. 2021 Jan;24(1):e25651.

10 Flanagan CF, McCann N, Stover J, Freedberg KA, Ciaranello AL. Do not forget the children: a model‐based analysis on the potential impact of COVID‐19‐associated interruptions in paediatric HIV prevention and care. J Int AIDS Soc. 2022 Jan;25(1):e25864.

11 Stanic T, McCann N, Penazzato M, Flanagan C, Essajee S, Freedberg KA, et al. Cost-effectiveness of routine provider-initiated testing and counseling for children with undiagnosed HIV in South Africa. Open Forum Infect Dis. 2022 Jan 1 (Vol. 9, No. 1, p. ofab603).

12 World Health Organization. Updated recommendations on HIV prevention, infant diagnosis, antiretroviral initiation and monitoring [cited 2021 April 23]. Available from: https://www.who.int/publications/i/item/9789240022232

13 Beste S, Essajee S, Siberry G, Hannaford A, Dara J, Sugandhi N, et al. Optimal antiretroviral prophylaxis in infants at high risk of acquiring HIV. Pediatr Infect Dis J. 2018 Feb 1;37(2):169-75.

14 Kumwenda NI, Hoover DR, Mofenson LM, Thigpen MC, Kafulafula G, Li Q, et al. Extended antiretroviral prophylaxis to reduce breast-milk HIV-1 transmission. N Engl J Med. 2008 Jul 10;359(2):119-29.

15 Hudgens MG, Taha TE, Omer SB, Jamieson DJ, Lee H, Mofenson LM, et al. Pooled individual data analysis of 5 randomized trials of infant nevirapine prophylaxis to prevent breast-milk HIV-1 transmission. Clin Infectious Dis. 2013 Jan 1;56(1):131-9.

16 Cunningham C, Capparelli E, McFarland E, Muresan P, Perlowski C, Yin DE, *et al.* Extended safety and PK of anti-HIV monoclonal Ab VRC07-523LS in HIV-exposed infants [cited 2022 October 6]. Available from: https://www.croiconference.org/abstract/extended-safety-and-pk-of-anti-hiv-monoclonal-ab-vrc07-523ls-in-hiv-exposed-infants/

17 McFarland EJ, Cunningham CK, Muresan P, Capparelli EV, Perlowski C, Morgan P, et al. Safety, Tolerability, and Pharmacokinetics of a Long-Acting Broadly Neutralizing Human Immunodeficiency Virus Type 1 (HIV-1). Monoclonal Antibody VRC01LS in HIV-1–Exposed Newborn Infants. J Infect Dis. 2021 Dec 1;224(11):1916-24.

18 Cunningham CK, McFarland EJ, Morrison RL, Capparelli EV, Safrit JT, Mofenson LM, et al. Safety, tolerability, and pharmacokinetics of the broadly neutralizing human immunodeficiency virus (HIV)-1 monoclonal antibody VRC01 in HIV-exposed newborn infants. J Infect Dis. 2020 Jul 23;222(4):628-36.

19 Abrams EJ, Capparelli E, Ruel T, Mirochnick M. Potential of long-acting products to transform the treatment and prevention of human immunodeficiency virus (HIV) in infants, children, and adolescents. Clin Infect Dis. 2022 Dec 1;75:S562-70.

20 Corey L, Gilbert PB, Juraska M, Montefiori DC, Morris L, Karuna ST, et al. Two randomized trials of neutralizing antibodies to prevent HIV-1 acquisition. N Engl J Med. 2021 Mar 18;384(11):1003-14.

21 Lorenzi JCC, Mendoza P, Cohen YZ, Nogueira L, Lavine C, Sapiente J, et al. Neutralizing activity of broadly neutralizing anti-HIV-1 antibodies against primary African isolates. J Virol. 2021 Feb 10;95(5):e01909-20.

22 Hessell AJ, Jaworski JP, Epson E, Matsuda K, Pandey S, Kahl C, et al. Early short-term treatment with neutralizing human monoclonal antibodies halts SHIV infection in infant macaques. Nat Med. 2016 Apr;22(4):362-8.

23 Shapiro MB, Cheever T, Malherbe DC, Pandey S, Reed J, Yang ES, et al. Single-dose bNAb cocktail or abbreviated ART post-exposure regimens achieve tight SHIV control without adaptive immunity. Nat Commun. 2020 Jan 7;11(1):70.

24 Anderson DJ Politch JA, Zeitlin L, Hiatt A, Kadasia K, Mayer KH, et al. Systemic and topical use of monoclonal antibodies to prevent the sexual transmission of HIV. AIDS. 2017 Jul 7;31(11):1505.

25 COVAX Working Group on delivery costs. Costs of delivering COVID-19 vaccine in 92 AMC countries [cited 2021 April 23]. Available from: https://www.who.int/publications/m/item/costs-of-delivering-covid-19-vaccine-in-92-amc-countries

26 Mvundura M, Lorenson K, Chweya A, Kigadye R, Bartholomew K, Makame M, et al. Estimating the costs of the vaccine supply chain and service delivery for selected districts in Kenya and Tanzania. Vaccine. 2015 May 28;33(23):2697-703.

27 Cunnama L, Abrams EJ, Myer L, Phillips TK, Dugdale CM, Ciaranello AL, et al. Provider‐and patient‐level costs associated with providing antiretroviral therapy during the postpartum phase to women living with HIV in South Africa: a cost comparison of three postpartum models of care. Trop Med Int Health. 2020 Dec;25(12):1553-67.

28 Neumann P, Sanders G, Russell L, Siegel J, Ganiats T. Cost-Effectiveness in Health and Medicine, Second Edition, 2nd ed. Oxford and New York: Oxford University Press, 2016.

29 Woods B, Revill P, Sculpher M, Claxton K. Country-level cost-effectiveness thresholds: initial estimates and the need for further research. Value Health. 2016 Dec 1;19(8):929-35.

30 Robinson LA, Hammitt JK, Chang AY, Resch S. Understanding and improving the one and three times GDP per capita cost-effectiveness thresholds. Health Policy Plan. 2017 Feb 1;32(1):141-5.

31 Meyer-Rath G, Van Rensburg C, Larson B, Jamieson L, Rosen S. Revealed willingness-to-pay versus standard cost-effectiveness thresholds: Evidence from the South African HIV Investment Case. PLoS One. 2017 Oct 26;12(10):e0186496.

32 Marseille E, Larson B, Kazi DS, Kahn JG, Rosen S. Thresholds for the cost–effectiveness of interventions: alternative approaches. Bull World Health Organ. 2014 Dec 15;93:118-24.

33 Ochalek J, Lomas J, Claxton K. Estimating health opportunity costs in low-income and middle-income countries: a novel approach and evidence from cross-country data. BMJ Global Health. 2018 Nov 1;3(6):e000964.

34 Jit M. Informing global cost-effectiveness thresholds using country investment decisions: human papillomavirus vaccine introductions in 2006-2018. Value Health. 2021 Jan 1;24(1):61-6.

35 Edoka IP, Stacey NK. Estimating a cost-effectiveness threshold for health care decision-making in South Africa. Health Policy Plan. 2020 Jun;35(5):546-55.

36 Dugdale CM, Ufio O, Alba C, Permar SR, Stranix‐Chibanda L, Cunningham CK, et al. Cost‐effectiveness of broadly neutralizing antibody prophylaxis for HIV‐exposed infants in sub‐Saharan African settings. J Int AIDS Soc. 2023 Jan;26(1):e26052.

37 The World Bank. GDP per capita (current US$) - South Africa, Zimbabwe, Cote d’Ivoire [cited 2020 September 9]. Available from: https://data.worldbank.org/indicator/NY.GDP.PCAP.CD?end=2019&locations=ZA-ZW-CI&start=2019&view=bar

38 Mandala J, Kasonde P, Badru T, Dirks R, Torpey K. HIV Retesting of HIV-negative pregnant women in the context of prevention of mother-to-child transmission of HIV in primary health centers in rural Zambia: what did we learn? J Int Assoc Provid AIDS Care. 2019 Jan 25;18:2325958218823530.

39 Rogers AJ, Akama E, Weke E, Blackburn J, Owino G, Bukusi EA, et al. Implementation of repeat HIV testing during pregnancy in southwestern Kenya: progress and missed opportunities. J Int AIDS Soc. 2017 Dec;20(4):e25036.

40 Heemelaar S, Habets N, Makukula Z, van Roosmalen J, van den Akker T. Repeat HIV testing during pregnancy and delivery: missed opportunities in a rural district hospital in Zambia. Trop Med Int Health. 2015 Mar;20(3):277-83.

41 de Beer S, Kalk E, Kroon M, Boulle A, Osler M, Euvrard J, et al. A longitudinal analysis of the completeness of maternal HIV testing, including repeat testing in Cape Town, South Africa. J Int AIDS Soc. 2020 Jan;23(1):e25441.

42 Ministère du Plan et du Développement. La Situation des Femmes et des Enfants en Côte d’Ivoire - Enquête à Indicateurs Multiples 2016 - MICS5 [cited 2022 October 6]. Available from: https://mics-surveys-prod.s3.amazonaws.com/MICS5/West%20and%20Central%20Africa/C%C3%B4te%20d%27Ivoire/2016/Final/Cote%20d%27Ivoire%202016%20MICS_French.pdf

43 South Africa National Department of Health. South Africa Demographic and Health Survey 2016 [cited 2022 October 6]. Available from: https://dhsprogram.com/pubs/pdf/FR337/FR337.pdf

44 Zimbabwe National Statistics Agency (ZIMSTAT), UNICEF. Zimbabwe Multiple Indicator Cluster Survey 2019, Survey Findings Report [cited 2020 August 4]. Available from: https://mics-surveys-prod.s3.amazonaws.com/MICS6/Eastern%20and%20Southern%20Africa/Zimbabwe/2019/Survey%20findings/Zimbabwe%202019%20MICS%20Survey%20Findings%20Report-31012020_English.pdf

45 Dinh T-H, Delaney KP, Goga A, Jackson D, Lombard C, Woldesenbet S, et al. Impact of maternal HIV seroconversion during pregnancy on early mother to child transmission of HIV (MTCT) measured at 4-8 weeks postpartum in South Africa 2011-2012: a national population-based evaluation. PLoS One. 2015 May 5;10(5):e0125525.

46 Mbizvo MT, Kasule J, Mahomed K, Nathoo K. HIV-1 seroconversion incidence following pregnancy and delivery among women seronegative at recruitment in Harare, Zimbabwe. Cent Afr J Med. 2001;47:115-8

47 Morrison CS, Wang J, Van Der Pol B, Padian N, Salata RA, Richardson BA. Pregnancy and the risk of HIV-1 acquisition among women in Uganda and Zimbabwe. AIDS. 2007 May 11;21(8):1027-34.

48 Teasdale CA, Abrams EJ, Chiasson MA, Justman J, Blanchard K, Jones HE. Incidence of sexually transmitted infections during pregnancy. PLoS One. 2018 May 24;13(5):e0197696.

49 South Africa National Department of Health. National Antenatal Sentinel HIV Survey Key Findings [cited 2022 October 6]. Available from: https://www.nicd.ac.za/wp-content/uploads/2019/07/Antenatal_survey-report_24July19.pdf

50 McCoy S, Koyuncu A, Kang-Dufour M, Mushavi A, Mahomva A, Padian N, *et al.* Approaching eMTCT in Zimbabwe: Expansion of PMTCT services and declining MTCT, 2012-2018 [cited 2022 October 6]. Availabe from: http://programme.ias2019.org/Abstract/Abstract/1921

51 Moyo F, Mazanderani AH, Murray T, Sherman GG, Kufa T. Achieving maternal viral load suppression for elimination of mother-to-child transmission of HIV in South Africa. AIDS. 2021 Feb 2;35(2):307-16.

52 Zimbabwe Ministry of Health and Child Care (MOHCC). Zimbabwe Population-Based HIV Impact Assessment (ZIMPHIA) 2020: Summary Sheet [cited 2020 December 28]. Available from: https://phia.icap.columbia.edu/wp-content/uploads/2020/11/ZIMPHIA-2020-Summary-Sheet_Web.pdf

53 Zimbabwe Ministry of Health and Child Care (MOHCC). Zimbabwe Population-Based HIV Impact Assessment (ZIMPHIA) 2015-2016: Final Report [cited 2022 October 6]. Available from: https://phia.icap.columbia.edu/wp-content/uploads/2019/08/ZIMPHIA-Final-Report_integrated_Web-1.pdf

54 Joint United Nations Programme on HIV/AIDS (UNAIDS). UNAIDS Data 2021 [cited 2022 May 10]. Available from: https://www.unaids.org/en/resources/documents/2021/2021_unaids_data

55 Ministère de Santé et de l’Hygiène Publique (MSHP). Côte d’Ivoire Population-Based HIV Impact Assessment (CIPHIA) 2017-2018: Final Report [cited 2022 October 6]. Available from: https://phia.icap.columbia.edu/wp-content/uploads/2021/05/CIPHIA-Final-Report_En.pdf

56 Elizabeth Glaser Pediatric AIDS Foundation. The Elizabeth Glaser Pediatric AIDS Foundation - Zimbabwe Annual Report, January - December 2018. Elizabeth Glaser Pediatric AIDS Foundation, 2019 [cited 2020 August 4]. Available from: https://www.pedaids.org/resource/the-elizabeth-glaser-pediatric-aids-foundation-zimbabwe-annual-report/

57 Chetty T Vandormael A, Thorne C, Coutsoudis A. Incident HIV during pregnancy and early postpartum period: a population-based cohort study in a rural area in KwaZulu-Natal, South Africa. BMC Pregnancy Childbirth. 2017 Dec;17(1):248.

58 Fatti G, Shaikh N, Jackson D, Goga A, Nachega JB, Eley B, et al. Low HIV incidence in pregnant and postpartum women receiving a community-based combination HIV prevention intervention in a high HIV incidence setting in South Africa. PLoS One. 2017 Jul 27;12(7):e0181691.

59 le Roux SM, Abrams EJ, Nguyen KK, Myer L. HIV incidence during breastfeeding and mother-to-child transmission in Cape Town, South Africa. AIDS. 2019 Jul 1;33(8):1399-401.

60 Joint United Nations Programme on HIV/AIDS (UNAIDS). UNAIDS Data 2020 [cited 2021 August 9]. Available from: https://www.unaids.org/en/resources/documents/2020/unaids-data

61 Desmonde S, Bangali M, Amorissani-Folquet M, Lenaud S, Karcher S, Lohoues-Kouacou M, *et al.* Effectiveness of a web-based information system to improve HIV early diagnosis and hepatitis B immunization coverages in Abidjan, Cote d’Ivoire. The DEPISTNEO project [cited 2022 October 6]. Available from: https://academicmedicaleducation.com/meeting/international-workshop-hiv-pediatrics-2019/abstract/effectiveness-web-based-information

62 Joint United Nations Programme on HIV/AIDS (UNAIDS). AIDSinfo 2021 [cited 2021 December 9]. Available from: https://aidsinfo.unaids.org/

63 Elizabeth Glaser Pediatric AIDS Foundation. Cote d’Ivoire Annual Report 2018 [cited 2020 August 4]. Available from: https://www.pedaids.org/resource/cote-divoire-annual-report-2018/

64 ICAP at Columbia University. Cote d’Ivoire Population-Based HIV Impact Assessment (CIPHIA) 2017-2018: Summary Sheet [cited 2022 October 6]. Available from: https://phia.icap.columbia.edu/wp-content/uploads/2018/08/CIPHIA_Cote-DIvoire-SS_FINAL.pdf

65 Dinh T, Mushavi A, Balachandra S, Shiraishi RW, Nyakura J, Munemo E, *et al.* Impact of option B+ and maternal HIV RNA viral load on mother-to-child HIV transmission: Findings from an 18-month prospective cohort study of a nationally representative sample of mother-infant pairs, Zimbabwe 2016-2017 [cited 2022 October 6]. Available from: http://programme.aids2018.org/Abstract/Abstract/6374

66 Nguyen K, Abrams EJ, Brittain K, Phillips TK, Ronan A, Zerbe A, *et al*. Breastfeeding cessation, maternal adherence to antiretroviral therapy and HIV viremia in the early postpartum period: a prospective cohort study [abstract P_100]. 8^th^ International Workshop on HIV and Pediatrics.

67 Watt MH, Cichowitz C, Kisigo G, Minja L, Knettel BA, Knippler ET, et al. Predictors of postpartum HIV care engagement for women enrolled in prevention of mother-to-child transmission (PMTCT) programs in Tanzania. AIDS Care. 2019 Jun 3;31(6):687-98.

68 Myer L, Phillips TK, Zerbe A, Brittain K, Lesosky M, Hsiao Ny, et al. Integration of postpartum healthcare services for HIV-infected women and their infants in South Africa: a randomised controlled trial. PLoS Med. 2018 Mar 30;15(3):e1002547.

69 Harrington BJ, Pence BW, Maliwichi M, Jumbe AN, Gondwe NA, Wallie SD, et al. Probable antenatal depression at antiretroviral initiation and postpartum viral suppression and engagement in Option B+. AIDS. 2018 Nov 11;32(18):2827.

70 Luoga E, Vanobberghen F, Bircher R, Nyuri A, Ntamatungiro AJ, Mnzava D, et al. Brief report: no HIV transmission from virally suppressed mothers during breastfeeding in rural Tanzania. J Acquir Immune Defic Syndr. 2018 Sep 1;79(1):e17-20.

71 Gill MM, Hoffman HJ, Ndatimana D, Mugwaneza P, Guay L, Ndayisaba GF, et al. 24-month HIV-free survival among infants born to HIV-positive women enrolled in Option B+ program in Kigali, Rwanda: The Kabeho Study. Medicine (Baltimore). 2017 Dec;96(51).

72 Hosseinipour M, Nelson JA, Trapence C, Rutstein SE, Kasende F, Kayoyo V, et al. Viral suppression and HIV drug resistance at 6 months among women in Malawi's Option B+ program: results From the PURE Malawi study. J Acquir Immune Defic Sydr. 2017 Jun 6;75(Suppl 2):S149.

73 Onoya D, Sineke T, Brennan AT, Long L, Fox MP. Timing of pregnancy, postpartum risk of virologic failure and loss to follow-up among HIV-positive women. AIDS. 2017 Jul 7;31(11):1593.

74 Davis NL, Miller WC, Hudgens MG, Chasela CS, Sichali D, Kayira D, et al. Maternal and breast milk viral load: impacts of adherence on peri-partum HIV infections Averted-the ban study. J Acquir Immune Defic Syndr. 2016 Dec 12;73(5):572.

75 Stover J, Glaubius R, Kassanjee R, Dugdale CM. Updates to the Spectrum/AIM model for the UNAIDS 2020 HIV estimates. J Int AIDS Soc. 2021 Sep;24:e25778.

76 UNICEF. Countdown to 2030 - Countdown Country Dashboards [cited 2022 October 6]. Available from: https://www.countdown2030.org/landing_page

77 Ciaranello A, Lu Z, Ayaya S, Losina E, Musick B, Vreeman R, et al. Incidence of World Health Organization stage 3 and 4 events, tuberculosis and mortality in untreated, HIV-infected children enrolling in care before 1 year of age: an IeDEA (International Epidemiologic Databases To Evaluate AIDS) east Africa regional analysis. Pediatr Infect Dis J. 2014 Jun 1;33(6):623-9.

78 West NS, Schwartz SR, Yende N, Schwartz SJ, Parmley L, Gadarowski MB, et al. Infant feeding by South African mothers living with HIV: implications for future training of health care workers and the need for consistent counseling. Int Breastfeed J. 2019 Dec;14(1):1-7.

79 Patel MR, Mushavi A, Balachandra S, Shambira G, Nyakura J, Mugurungi O, et al. HIV-exposed Uninfected Infant Morbidity and Mortality within a Nationally Representative Prospective Cohort of Mother-Infant Pairs in Zimbabwe, 2013–2014. AIDS. 2020 Jul 7;34(9):1339.

80 World Health Organization. Consolidated guidelines on the use of antiretroviral drugs for the treating and preventing HIV infection: recommendations for a public health approach, 2nd ed [cited 2020 July 23]. Available from: https://apps.who.int/iris/handle/10665/208825

81 UNAIDS Reference Group on Estimates, Modelling, and Projections. Modelling Paediatric HIV and the need for ART [cited 2021 April 21]. Available from: http://epidem.org/modelling-paediatric-hiv-and-the-need-for-art-october-2020.

82 Stover J, Glaubius R, Mofenson L, Dugdale CM, Davies MA, Patten G, et al. Updates to the Spectrum/AIM model for estimating key HIV indicators at national and subnational levels. AIDS. 2019 Dec 12;33(Suppl 3):S227.

83 Komtenza B, Satyanarayana S, Takarinda KC, Mukungunugwa SH, Mugurungi O, Chonzi P, et al. Identifying high or low risk of mother to child transmission of HIV: How Harare City, Zimbabwe is doing? PLoS One. 2019 Mar 13;14(3):e0212848.

84 Moyo F, Mazanderani AH, Barron P, Bhardwaj S, Goga AE, Pillay Y, et al. Introduction of routine HIV birth testing in the South African National Consolidated Guidelines. Pediatr Infect Dis J. 2018 Jun 1;37(6):559-63.

85 Kalk E, Kroon M, Boulle A, Osler M, Euvrard J, Stinson K, et al. Neonatal and infant diagnostic HIV‐PCR uptake and associations during three sequential policy periods in Cape Town, South Africa: a longitudinal analysis. J Int AIDS Soc. 2018 Nov;21(11):e25212.

86 Phelanyane F, Boulle A, and Kalk E. Prevention of mother-to-child-transmission (PMTCT) of HIV in Khayelitsha, South Africa: A contemporary review of the service 20 years later [cited 2020 July 28]. Available from: https://www.aids2020.org/wp-content/uploads/2020/09/AIDS2020_Abstracts.pdf

87 Tait CL, Peters RP, McIntyre JA, Mnyani CN, Chersich MF, Gray G, et al. Implementation of a PMTCT programme in a high HIV prevalence setting in Johannesburg, South Africa: 2002–2015. South Afr J HIV Med. 2020 Jan 1;21(1):1-7.

88 Spooner E, Govender K, Reddy T, Ramjee G, Mbadi N, Singh S, et al. Point-of-care HIV testing best practice for early infant diagnosis: an implementation study. BMC Public Health. 2019 Dec;19(1):1-4.

89 Coulon J, Isaacs Z, Bisschoff C, Van Zyl R, Van der Linde L, Wilson L, *et al*. HIV testing at birth: Are we getting it right? South Afr J HIV Med. 2019 Jan 1;20(1):1-5.

90 Onoya D, Jinga N, Nattey C, Mongwenyana C, Mngadi S, Sherman GG. Motivational interviewing retention counseling and child HIV testing in South Africa [cited 2020 July 24]. Available from: https://www.croiconference.org/wp-content/uploads/sites/2/resources/2020/ebook/croi2020-boston-abstract-ebook.pdf

91 Massyn N, Pillay Y, Padarath A. District Health Barometer 2017/18. Durban, South Africa: Health Systems Trust, 2019 [cited 2020 August 11]. Available from: https://www.hst.org.za/publications/Pages/DHB20172018.aspx

92 Mallampati D, Ford N, Hanaford A, Sugandhi N, Penazzato M. Performance of virological testing for early infant diagnosis: a systematic review. J Acquir Immune Defic Syndr. 2017 Jul 1;75(3):308-14.

93 Bianchi F, Nzima V, Chadambuka A, Mataka A, Gcinile N, Ndayisaba G, *et al.* Comparing conventional to point-of-care (POC) early infant diagnosis (EID): Pre and post intervention data from a multi-country evaluation (Abstract #TUSA1302) [cited 2020 October 14]. 9^th^ International AIDS Society Conference on HIV Science, 2017.

94 Mazanderani A, Moyo F, Sherman GG. Missed diagnostic opportunities within South Africa’s early infant diagnosis program, 2010–2015. PloS One. 2017 May 11;12(5):e0177173.

95 Bianchi F, Cohn J, Sacks E, Bailey R, Lemaire JF, Machekano R, et al. Evaluation of a routine point-of-care intervention for early infant diagnosis of HIV: an observational study in eight African countries. Lancet HIV. 2019 Jun 1;6(6):e373-81.

96 Ngoma K, Moyo F, Haeri Mazanderani A, Feucht UD, Bhardwaj S, Goosen M, et al. Monitoring diagnosis, retention in care and viral load suppression in children testing HIV polymerase chain reaction-positive in two districts in South Africa. S Afr Med J. 2019 Sep 1;109(9):686-92.

97 Smith ER, Sheahan AD, Heyderman RS, Miller WC, Wheeler S, Hudgens M, et al. Performance of HIV rapid tests among breastfeeding, Malawian infants. Pediatr Infect Dis J. 2017 Apr 1;36(4):405-11.

98 Buchanan AM, Nadjm B, Amos B, Mtove G, Sifuna D, Cunningham CK, et al. Utility of rapid antibody tests to exclude HIV-1 infection among infants and children aged< 18 months in a low-resource setting. J Clin Virol. 2012 Nov 1;55(3):244-9.

99 Desmond AC, Moodley D, Conolly CA, Castel SA, Coovadia HM. Evaluation of adherence measures of antiretroviral prophylaxis in HIV exposed infants in the first 6 weeks of life. BMC Pediatr. 2015 Dec;15(1):1-8.

100 World Health Organization. Immunization coverage survey data [cited 2021 December 13]. Available from: https://data.unicef.org/wp-content/uploads/2021/07/Immunization-coverage-data.xls

101 Coovadia HM, Brown ER, Fowler MG, Chipato T, Moodley D, Manji K, et al. Efficacy and safety of an extended nevirapine regimen in infant children of breastfeeding mothers with HIV-1 infection for prevention of postnatal HIV-1 transmission (HPTN 046): a randomised, double-blind, placebo-controlled trial. Lancet. 2012 Jan 21;379(9812):221-8.

102 Chasela CS, Hudgens MG, Jamieson DJ, Kayira D, Hosseinipour MC, Kourtis AP, et al. Maternal or infant antiretroviral drugs to reduce HIV-1 transmission. N Engl J Med. 2010 Jun 17;362(24):2271-81.

103 Flynn PM, Taha TE, Cababasay M, Fowler MG, Mofenson LM, Owor M, et al. Prevention of HIV-1 transmission through breastfeeding: efficacy and safety of maternal antiretroviral therapy versus infant nevirapine prophylaxis for duration of breastfeeding in HIV-1-infected women with high CD4 cell count (IMPAACT PROMISE): a randomized, open label, clinical trial. J Acquir Immune Defic Syndr. 2018 Apr 4;77(4):383.

104 Mellors JW, Munoz A, Giorgi JV, Margolick JB, Tassoni CJ, Gupta P, et al. Plasma viral load and CD4+ lymphocytes as prognostic markers of HIV-1 infection. Ann Intern Med. 1997 Jun 15;126(12):946-54.

105 Holmes CB, Wood R, Badri M, Zilber S, Wang B, Maartens G, et al. CD4 decline and incidence of opportunistic infections in Cape Town, South Africa: implications for prophylaxis and treatment. J Acquir Immune Defic Syndr. 2006 Aug 1;42(4):464-9.

106 Anglaret X, Chêne G, Attia A, Toure S, Lafont S, Combe P, et al. Early chemoprophylaxis with trimethoprim-sulphamethoxazole for HIV-1-infected adults in Abidjan, Côte d'Ivoire: a randomised trial. Lancet. 1999 May 1;353(9163):1463-8.

107 Marston M, Becquet R, Zaba B, Moulton LH, Gray G, Coovadia H, et al. Net survival of perinatally and postnatally HIV-infected children: a pooled analysis of individual data from sub-Saharan Africa. Int J Epidemiol. 2011 Apr 1;40(2):385-96.

108 Becquet R, Marston M, Dabis F, Moulton LH, Gray G, Coovadia HM, et al. Children who acquire HIV infection perinatally are at higher risk of early death than those acquiring infection through breastmilk: a meta-analysis. PloS One. 2012 Feb 23;7(2):e28510.

109 United Nations, Department of Economic and Social Affairs, Population Division. World Population Prospects 2019, Online Edition. Rev. 1. 2019 [cited 2021 July 27]. Available from: https://population.un.org/wpp/Download/Standard/CSV/.

110 World Health Organization. Global Health Estimates 2020: Deaths by Cause, Age, Sex, by Country and by Region, 2000-2019 [cited 2021 July 27]. Available from: https://www.who.int/data/maternal-newborn-child-adolescent-ageing/advisory-groups/gama/gama-advisory-group-members

111 Yazdanpanah Y, Losina E, Anglaret X, Goldie SJ, Walensky RP, Weinstein MC, et al. Clinical impact and cost-effectiveness of co-trimoxazole prophylaxis in patients with HIV/AIDS in Côte d’Ivoire: a trial-based analysis. AIDS. 2005 Aug 12;19(12):1299-308.

112 Amani-Bosse C, Dahourou DL, Malateste K, Amorissani‐Folquet M, Coulibaly M, Dattez S, Emieme A, et al Virological response and resistances over 12 months among HIV‐infected children less than two years receiving first‐line lopinavir/ritonavir‐based antiretroviral therapy in Cote d’Ivoire and Burkina Faso: the MONOD ANRS 12206 cohort. J Int AIDS Soc. 2017;20(1):21362.

113 Mulenga V, Musiime V, Kekitiinwa A, Cook AD, Abongomera G, Kenny J, et al. Abacavir, zidovudine, or stavudine as paediatric tablets for African HIV-infected children (CHAPAS-3): an open-label, parallel-group, randomised controlled trial. Lancet Infect Dis. 2016 Feb 1;16(2):169-79.

114 Ferrand RA, Simms V, Dauya E, Bandason T, Mchugh G, Mujuru H, et al. The effect of community-based support for caregivers on the risk of virological failure in children and adolescents with HIV in Harare, Zimbabwe (ZENITH): an open-label, randomised controlled trial. Lancet Child Adolesc Health. 2017 Nov 1;1(3):175-83.

115 Jiamsakul A, Kariminia A, Althoff KN, Cesar C, Cortes CP, Davies MA, et al. HIV viral load suppression in adults and children receiving antiretroviral therapy–results from the IeDEA collaboration. J Acquir Defic Syndr. 2017 Nov 11;76(3):319.

116 Kityo C, Boerma RS, Sigaloff KC, Kaudha E, Calis JC, Musiime V, et al. Pretreatment HIV drug resistance results in virological failure and accumulation of additional resistance mutations in Ugandan children. J Antimicrob Chemother. 2017 Sep 1;72(9):2587-95.

117 Teasdale CA, Sogaula N, Yuengling KA, Wang C, Mutiti A, Arpadi S, et al. HIV viral suppression and longevity among a cohort of children initiating antiretroviral therapy in Eastern Cape, South Africa. J Int AIDS Soc. 2018 Aug;21(8):e25168.

118 Moyo S, Ncube RT, Shewade HD, Ngwenya S, Ndebele W, Takarinda KC, et al. Children and adolescents on anti-retroviral therapy in Bulawayo, Zimbabwe: How many are virally suppressed by month six? F1000Res. 2020;9.

119 Boerma RS, Bunupuradah T, Dow D, Fokam J, Kariminia A, Lehman D, et al. Multicentre analysis of second‐line antiretroviral treatment in HIV‐infected children: adolescents at high risk of failure. J Int AIDS Soc. 2017;20(1):21930.

120 Venter WDF, Moorhouse M, Sokhela S, Fairlie L, Mashabane N, Masenya M, et al. Dolutegravir plus two different prodrugs of tenofovir to treat HIV. N Engl J Med. 2019 Aug 29;381(9):803-15.

121 NAMSAL ANRS 12313 Study Group, Kouanfack C, Mpoudi-Etame M, Bassega P, Eymard-Duvernay S, Leroy s, et al. Dolutegravir-based or low-dose efavirenz–based regimen for the treatment of HIV-1. N Engl J Med. 2019 Aug 29;381(9):816-26.

122 Stockdale AJ, Saunders MJ, Boyd MA, Bonnett LJ, Johnston V, Wandeler G, et al. Effectiveness of protease inhibitor/nucleos (t) ide reverse transcriptase inhibitor–based second-line antiretroviral therapy for the treatment of human immunodeficiency virus type 1 infection in sub-Saharan Africa: a systematic review and meta-analysis. Clin Infect Dis. 2018 Jun 1;66(12):1846-57.

123 Venter WDF, Sokhela S, Simmons B, Moorhouse M, Fairlie L, Mashabane N, et al. Dolutegravir with emtricitabine and tenofovir alafenamide or tenofovir disoproxil fumarate versus efavirenz, emtricitabine, and tenofovir disoproxil fumarate for initial treatment of HIV-1 infection (ADVANCE): week 96 results from a randomised, phase 3, non-inferiority trial. Lancet HIV. 2020 Oct 1;7(10):e666-76.

124 Calmy A, Sanchez TT, Kouanfack C, Mpoudi-Etame M, Leroy S, Perrineau S, et al. Dolutegravir-based and low-dose efavirenz-based regimen for the initial treatment of HIV-1 infection (NAMSAL): week 96 results from a two-group, multicentre, randomised, open label, phase 3 non-inferiority trial in Cameroon. Lancet HIV. 2020 Oct 1;7(10):e677-87.

125 Paton NI, Kityo C, Hoppe A, Reid A, Kambugu A, Lugemwa A, et al. Assessment of second-line antiretroviral regimens for HIV therapy in Africa. N Engl J Med. 2014 Jul 17;371(3):234-47.

126 Ford N, Orrell C, Shubber Z, Apollo T, Vojnov L. HIV viral resuppression following an elevated viral load: a systematic review and meta‐analysis. J Int AIDS Soc. 2019 Nov;22(11):e25415.

127 Pepperrell T, Venter WD, McCann K, Bosch B, Tibbatts M, Woods J, et al. Participants on dolutegravir resuppress human immunodeficiency virus RNA after virologic failure: updated data from the ADVANCE Trial. Clin Infect Dis. 2021 Aug 15;73(4):e1008-10.

128 Eholie SP, Moh R, Benalycherif A, Gabillard D, Ello F, Messou E, et al. Implementation of an intensive adherence intervention in patients with second-line antiretroviral therapy failure in four west African countries with little access to genotypic resistance testing: a prospective cohort study. Lancet HIV. 2019 Nov 1;6(11):e750-9.

129 Violari A, Lindsey JC, Hughes MD, Mujuru HA, Barlow-Mosha L, Kamthunzi P, et al. Nevirapine versus ritonavir-boosted lopinavir for HIV-infected children. N Engl J Med. 2012 Jun 21;366(25):2380-9.

130 Palumbo P, Lindsey JC, Hughes MD, Cotton MF, Bobat R, Meyers T, et al. Antiretroviral treatment for children with peripartum nevirapine exposure. N Engl J Med. 2010 Oct 14;363(16):1510-20.

131 Walmsley SL, Antela A, Clumeck N, Duiculescu D, Eberhard A, Gutiérrez F, et al. Dolutegravir plus abacavir–lamivudine for the treatment of HIV-1 infection. N Engl J Med. 2013 Nov 7;369(19):1807-18.

132 Losina E, Yazdanpanah Y, Deuffic-Burban S, Wang B, Wolf LL, Messou E, et al. The independent effect of highly active antiretroviral therapy on severe opportunistic disease incidence and mortality in HIV-infected adults in Côte d'Ivoire. Antiviral Therapy. 2007 May;12(4):543-51.

133 Carlucci JG, Liu Y, Clouse K, Vermund SH. Attrition of HIV-positive children from HIV services in low-and middle-income countries: a systematic review and meta-analysis. AIDS. 2019 Dec 12;33(15):2375.

134 Makurumidze R, Mutasa-Apollo T, Decroo T, Choto RC, Takarinda KC, Dzangare J, et al. Retention and predictors of attrition among patients who started antiretroviral therapy in Zimbabwe’s national antiretroviral therapy programme between 2012 and 2015. PLoS One. 2020 Jan 7;15(1):e0222309.

135 Fenner L, Brinkhof MW, Keiser O, Weigel R, Cornell M, Moultrie H, et al. Early mortality and loss to follow-up in HIV-infected children starting antiretroviral therapy in Southern Africa. J Acquir Immune Defic Syndr. 2010 Aug 8;54(5):524.

136 Sengayi M, Dwane N, Marinda E, Sipambo N, Fairlie L, Moultrie H. Predictors of loss to follow-up among children in the first and second years of antiretroviral treatment in Johannesburg, South Africa. Glob Health Action. 2013 Dec 1;6(1):19248.

137 Chhagan MK, Kauchali S, Van den Broeck J. Clinical and contextual determinants of anthropometric failure at baseline and longitudinal improvements after starting antiretroviral treatment among South African children. Trop Med Int Health. 2012 Sep;17(9):1092-9.

138 Janssen N, Ndirangu J, Newell ML, Bland RM. Successful paediatric HIV treatment in rural primary care in Africa. Arch Dis Child. 2010 Jun 1;95(6):414-21.

139 Zanoni BC, Phungula T, Zanoni HM, France H, Feeney ME. Risk factors associated with increased mortality among HIV infected children initiating antiretroviral therapy (ART) in South Africa. PloS One. 2011 Jul 29;6(7):e22706.

140 Meyer-Rath G, Brennan A, Long L, Ndibongo B, Technau K, Moultrie H, et al. Cost and outcomes of paediatric antiretroviral treatment in South Africa. AIDS. 2013 Jan 14;27(2):243-50.

141 Chandiwana N, Sawry S, Chersich M, Kachingwe E, Makhathini B, Fairlie L. High loss to follow-up of children on antiretroviral treatment in a primary care HIV clinic in Johannesburg, South Africa. Medicine (Baltimore). 2018 Jul;97(29).

142 Kalawan V, Naidoo K, Archary M. Impact of routine birth early infant diagnosis on neonatal HIV treatment cascade in eThekwini district, South Africa. South Afr J HIV Med. 2020;21(1).

143 Bernard C, Balestre E, Coffie PA, Eholie SP, Messou E, Kwaghe V, et al. Aging with HIV: what effect on mortality and loss to follow-up in the course of antiretroviral therapy? The IeDEA West Africa Cohort Collaboration. HIV AIDS (Auckl). 2018 Nov 16:239-52.

144 Matare T, Shewade HD, Ncube RT, Masunda K, Mukeredzi I, Takarinda KC, et al. Anti-retroviral therapy after “Treat All” in Harare, Zimbabwe: What are the changes in uptake, time to initiation and retention? F1000Res. 2020;9.

145 Makurumidze R, Buyze J, Decroo T, Lynen L, de Rooij M, Mataranyika T, et al. Patient-mix, programmatic characteristics, retention and predictors of attrition among patients starting antiretroviral therapy (ART) before and after the implementation of HIV “Treat All” in Zimbabwe. PLoS One. 2020 Oct 19;15(10):e0240865.

146 Dorward J, Sookrajh Y, Gate K, Khubone T, Mtshaka N, Mlisana K, et al. HIV treatment outcomes among people with initiation CD4 counts> 500 cells/µL after implementation of Treat All in South African public clinics: a retrospective cohort study. J Int AIDS Soc. 2020 Apr;23(4):e25479.

147 Hirasen K, Fox MP, Hendrickson CJ, Sineke T, Onoya D. HIV treatment outcomes among patients initiated on antiretroviral therapy pre and post-universal test and treat guidelines in South Africa. Ther Clin Risk Manag. 2020 Mar 4;16:169-80.

148 Ambia J, Kabudula C, Risher K, Xavier Gómez‐Olivé F, Rice BD, Etoori D, et al. Outcomes of patients lost to follow‐up after antiretroviral therapy initiation in rural north‐eastern South Africa. Trop Med Int Health. 2019 Jun;24(6):747-56.

149 The Global Fund. HIV Viral Load and Early Infant Diagnosis Selection and Procurement Information Tool [cited 2020 August 5]. Available from: <https://www.theglobalfund.org/media/5765/psm_viralloadearlyinfantdiagnosis_content_en.pdf?u=637298970370000000>

150 National Health Laboratory Service. NHLS State Price List 2019/20. South African National Health Laboratory Service, 2019.

151 Bassett IV, Giddy J, Nkera J, Wang B, Losina E, Lu Z, et al. Routine voluntary HIV testing in Durban, South Africa: the experience from an outpatient department. J Acquir Immune Defic Syndr. 2007 Oct 10;46(2):181.

152 The Global Fund. Price list [cited 2020 October 27]. Available from: https://public.tableau.com/profile/the.global.fund#!/vizhome/PQRPricelist_English/PriceList

153 Doherty K, Essajee S, Penazzato M, Holmes C, Resch S, Ciaranello A. Estimating age-based antiretroviral therapy costs for HIV-infected children in resource-limited settings based on World Health Organization weight-based dosing recommendations. BMC Health Serv Res. 2014 Dec;14(1):1-2.

154 The Global Fund. Pooled procurement mechanism reference pricing: ARVs (version: quarter 3 2020). Available from: https://www.theglobalfund.org/media/5813/ppm_arvreferencepricing_table_en.pdf.

155 Desmonde S, Avit D, Petit J, Amorissani Folquet M, Eboua FT, Amani Bosse C, et al. Costs of care of HIV-infected children initiating lopinavir/ritonavir-based antiretroviral therapy before the age of two in Cote d’Ivoire. PLos One. 2016 Dec 9;11(12):e0166466.

156 Menzies NA, Berruti AA, Berzon R, Filler S, Ferris R, Ellerbrock TV, et al. The cost of providing comprehensive HIV treatment in PEPFAR-supported programs. AIDS. 2011 Sep 9;25(14):1753.

157 Magure T, Manenji A, Gboun M, *et al.* Zimbabwe National AIDS Spending Assessment: Consolidated Report 2011 and 2012. National AIDS Council of Zimbabwe and UNAIDS, 2012.

158 Massyn N, Barron P, Day C, Padarath A. District Health Barometer 2018/19. Durban, South Africa: Health Systems Trust, 2020 [cited 2020 August 17]. Available from: https://www.hst.org.za/publications/Pages/DISTRICT-HEALTH-BAROMETER-201819.aspx.

159 The Global Fund. Pooled Procurement Mechanism Reference Pricing: Advanced HIV disease products [cited 2020 August 20]. Available from: https://www.theglobalfund.org/media/9274/ppm_advancedhivproductsreferencepricing_table_en.pdf?u=637319004622070000

160 Clinton Health Access Initiative. Unitaid and CHAI announce agreement with Omega Diagnostics to increase access to new, instrument-free CD4 test for people living with HIV in over 130 low- and middle-income countries [cited 2020 August 4]. Available from: https://www.clintonhealthaccess.org/unitaid-and-chai-announce-agreement-with-omega-diagnostics-to-increase-access-to-new-portable-cd4-testing-device-for-people-living-with-hiv-in-over-130-low-and-middle-income-countries/

161 Tibuyile Sigudia (Central Medical Stores Swaziland). Personal communication. 2016.

162 Thomas LS. Costing of HIV/AIDS services at a tertiary level hospital in Gauteng Province; 2007. Available from: http://hdl.handle.net/10539/2008.

163 Cleary S, Chitha W, Jikwana S, Okorafor OA, Boulle A. South African Health Review 2005: Financing. Durban, South Africa: Health Systems Trust, 2005. Available from: https://www.hst.org.za/publications/South%20African%20Health%20Reviews/sahr05.pdf

164 World Health Organization. WHO-CHOICE: Country-specific unit costs; 2011 [cited 2021 August 18]. Available from: https://www.who.int/teams/health-systems-governance-and-financing/economic-analysis/costing-and-technical-efficiency/quantities-and-unit-prices-(cost-inputs)/econometric-estimation-of-who-choice-country-specific-costs-for-inpatient-and-outpatient-health-service-delivery.

165 Zimbabwe National Statistics Agency. Demographic and Health Survey 2015; 2016. Available from: https://dhsprogram.com/pubs/pdf/FR322/FR322.pdf.

166 Zunza M, Esser M, Slogrove A, Bettinger JA, Machekano R, Cotton MF, et al. Cotton. Early breastfeeding cessation among HIV-infected and HIV-uninfected women in Western Cape Province, South Africa. AIDS Behav. 2018 Jul;22:114-20.

167 Nielsen-Saines K, Watts DH, Veloso VG, Bryson YJ, Joao EC, Pilotto JH, et al. Three postpartum antiretroviral regimens to prevent intrapartum HIV infection. N Engl J Med. 2012 Jun 21;366(25):2368-79.

168 Lallemant M, Amzal B, Sripan P, Urien S, Cressey TR, Ngo-Giang-Huong N, et al. Perinatal antiretroviral intensification to prevent intrapartum HIV transmission when antenatal antiretroviral therapy is initiated less than 8 weeks before delivery. J Acquir Immune Defic Syndr. 2020 Jul 7;84(3):313.
